# Estimating the burden of diseases attributable to lead exposure in the North Africa and Middle East region, 1990–2019: a systematic analysis for the Global Burden of Disease study 2019

**DOI:** 10.1186/s12940-022-00914-3

**Published:** 2022-10-29

**Authors:** Malihe Rezaee, Zahra Esfahani, Seyed Aria Nejadghaderi, Mohsen Abbasi-Kangevari, Sahar Saeedi Moghaddam, Ali Ghanbari, Azin Ghamari, Ali Golestani, Elmira Foroutan Mehr, Ameneh Kazemi, Rosa Haghshenas, Mahsa Moradi, Farzad Kompani, Negar Rezaei, Bagher Larijani

**Affiliations:** 1 Non-Communicable Diseases Research Center, Endocrinology and Metabolism Population Sciences Institute, Tehran University of Medical Sciences, Tehran, Iran; 2 Tehran Heart Center, Cardiovascular Diseases Research Institute, Tehran University of Medical Sciences, Tehran, Iran; 3 School of Medicine, Shahid Beheshti University of Medical Sciences, Tehran, Iran; 4 Department of Biostatistics, University of Social Welfare and Rehabilitation Sciences, Tehran, Iran; 5 Department of Environmental Health Engineering, School of Public Health, Tehran University of Medical Sciences, Tehran, Iran; 6 Division of Hematology and Oncology, Children’s Medical Center, Pediatrics Center of Excellence, Tehran University of Medical Sciences, Tehran, Iran; 7 Endocrinology and Metabolism Research Center, Endocrinology and Metabolism Clinical Sciences Institute, Tehran University of Medical Sciences, Tehran, Iran

**Keywords:** Lead exposure, Disability-adjusted life years, Death, Global Burden of Disease, North Africa, Middle East

## Abstract

**Background:**

Lead exposure (LE) and its attributable deaths and disability-adjusted life years (DALYs) have declined in the recent decade; however, it remains one of the leading public health concerns, particularly in regions with low socio-demographic index (SDI) such as the North Africa and Middle East (NAME) region. Hence, we aimed to describe the attributable burden of the LE in this region.

**Methods:**

Data on deaths, DALYs, years of life lost (YLLs), and years lived with disability (YLDs) attributable to LE in the NAME region and its 21 countries from 1990 to 2019 were extracted from the Global Burden of Disease (GBD) 2019 study.

**Results:**

In 2019, the age-standardized death and DALY rates attributable to LE were 23.4 (95% uncertainty interval: 15.1 to 33.3) and 489.3 (320.5 to 669.6) per 100,000 in the region, respectively, both of which were higher among men than women. The overall age-standardized death and DALY rates showed 27.7% and 36.8% decreases, respectively, between 1990 and 2019. In this period, Bahrain, the United Arab Emirates, and Turkey had the highest decreases in the age-standardized death and DALY rates, while Afghanistan, Egypt, and Yemen had the lowest ones. Countries within high SDI quintile had lower attributable burden to LE compared with the low SDI quintile. Cardiovascular diseases and chronic kidney diseases accounted for the 414.2 (258.6 to 580.6) and 28.7 (17.7 to 41.7) LE attributable DALYs per 100,000 in 2019, respectively. The attributable YLDs was 46.4 (20.7 to 82.1) per 100,000 in 2019, which shows a 25.7% reduction (-30.8 to -22.5%) over 1990–2019.

**Conclusions:**

The overall LE and its attributed burden by cause have decreased in the region from 1990–2019. Nevertheless, the application of cost-effective and long-term programs for decreasing LE and its consequences in NAME is needed.

**Supplementary Information:**

The online version contains supplementary material available at 10.1186/s12940-022-00914-3.

## Background

Lead is an abundant, highly toxic heavy metal known as one of the oldest environmental and occupational pollutants worldwide. The primary sources of environmental and occupational lead contamination are industrial activities such as metal mining, smelting, recycling, and manufacturing of many products, particularly lead-acid batteries. However, leaded paint and leaded aviation fuel such as gasoline have remained as the remarkable sources in some countries [1–3]. Despite significant reductions in environmental sources of lead in recent decades, low-level lead exposure (LE) remains a significant global public health concern in many countries [4]. Based on the Global Burden of Disease (GBD) reports of global burden of 87 risk factors in 204 countries and terrirtories, the global burden of LE in terms of disability-adjusted life years (DALYs) has declined nearly 1% annually between 1990 and 2019, and the overall trend was not increasing [5]. Moreover, the pooled mean blood lead levels in 44 low- and middle-income countries (LMICs) ranged from 1.66 µg/dL to 9.30 µg/dL in children and from 0.39 µg/dL to 11.36 µg/dL in adults [6]. The US Centers for Disease Control and Prevention (CDC) set a blood lead reference value of 3.5 µg/dL for children and a surveillance case definition of 5 µg/dL for adult [7]. The World Health Organization (WHO) has estimated that in 2019, LE accounted for nearly a million deaths and 21.7 million DALYs worldwide, imposing its highest burden in LMICs [8].

The brain is the most susceptible organ to LE-related damages and LE, even at low levels, has deleterious effects on intellectual and neuropsychological development [9]. It has also been reported that every 10 μg/dL increment in blood lead level was associated with mean reduction in intelligence quotient (IQ) score of two points [10]. A study demonestrated that the ability of decision making was impaired in individuals with blood lead level up to 40 μg/dL [11]. Besides, the adverse effects of increased blood lead levels on memory function and human language-related capabilities were reported [12]. In addition, peripheral motor neuropathy is associated with chronic high-level LE [13]. LE is a well-recognized risk factor for morbidity and mortality attributable to cardiovascular diseases (CVDs), including hypertension, atherosclerosis, ischemic heart disease, peripheral arterial disease, left-ventricular hypertrophy, and stroke [14–17]. Also, the relationship between CVDs and LE may result from lead toxicity-induced oxidative stress [18]. Renal tubular damage and nephropathy were reported following exposure to high lead levels and chronic low-level LE, respectively [19, 20]. Moreover, LE could be associated with cancers, osteoporosis or osteomalacia development, hematological abnormalities, and impaired reproductive function [21].

The burden of disease attributable to LE is often unrecognized and unconsidered in regions with low socioeconomic status. In this study, we aimed to describe the burden attributable to LE from 1990 to 2019 in countries of the North Africa and Middle East (NAME) region based on the latest estimated data from the GBD 2019 study.

## Methods

We used the GBD 2019 data on deaths, DALYs, years of life lost (YLLs), and years lived with disability (YLDs) attributable to LE in the NAME region and its 21 countries and territories. The NAME region is one of the 21 GBD regions, which include the following countries: Afghanistan, Algeria, Bahrain, Egypt, Iran (Islamic Republic of), Iraq, Jordan, Kuwait, Lebanon, Libya, Morocco, Oman, Palestine, Qatar, Saudi Arabia, Sudan, the Syrian Arab Republic, Tunisia, Turkey, the United Arab Emirates, and Yemen. The GBD project is conducted by the Institute for Health Metrics and Evaluation and aims to measure the global, regional, and national burden of diseases and injuries and the attributable burden of risk factors. In the GBD 2019 project, data on the burden of 369 diseases and injuries and 87 risk factors in 204 countries and territories located in 21 GBD regions or seven super-regions have been reported. The details on the methodology have been provided elsewhere [5, 22], also available at https://vizhub.healthdata.org/gbd-compare and http://ghdx.healthdata.org/gbd-results-tool.

### Definitions

According to the currently known pathways of attributable health loss, LE is classified in two ways, acute and chronic LE. Acute LE, measured in micrograms per deciliter of blood (µg/dL), has been linked to intelligence quality decline in children. Chronic LE, measured in micrograms of lead per gram of bone (µg/g), is linked to elevated systolic blood pressure (SBP) and CVDs. LE and residential radon are level 3 risk factors in the category of other environmental risks (Additional file 1) [5].

The socio-demographic index (SDI) is a multifactor measure of socioeconomic development that includes lag-distributed income per capita, educational attainment for those above the age of 15, and the total fertility rate for people under the age of 25. The SDI scale runs from 0 to 1, with 0 being the least developed and one being the most developed. Countries are categorized into five quintiles based on SDI level: low, low-middle, middle, high-middle, and high SDI. The age classification was in 14 age groups which were < 20, five-year intervals from 20–24 to 75–79 years, and ≥ 80 years old.

### Data sources

LE data were derived from literature reports and surveys on the blood lead levels. These studies resulted from literature review of the last updated GBD 2017, which included 3,183 usable data points from 554 studies between 1970 to 2017. Blood lead levels were generated from investigations in blood samples which were taken and analyzed using various procedures. Calculating a cumulative blood lead index for cohorts using predicted blood lead over their lifespan was used to assess the second pathway of burden, bone lead [5].

### Data processing and modeling strategy

In GBD 2013, the modelling strategy changed from age-integrating Bayesian hierarchal modelling (DisMod-MR) to a spatiotemporal Gaussian process regression (ST-GPR) methodology. The ST-GPR modeling approach was modified for GBD 2019, which applies to a wide range of risk variables. Covariates developed over time and location relevant to this research were utilized to predict blood lead in country-years with insufficient data. The SDI, urbanicity, the total number of two- and four-wheeled vehicles per capita, and a covariate indicating whether leaded gasoline had been phased out in a specific country-year were shown to have the predictive potential for blood LE. From 1970 to 2019, ST-GPR was used to calculate blood lead mean and standard deviation for all age groups, sexes, and GBD regions. To establish blood LE distributions, the ST-GPR mean and standard deviation estimations for blood lead were combined with the global distribution shape. In the end, 11 separate probability distributions were included in the distribution [5].

### Threshold lead level

Blood lead was assumed to develop linearly from 2.0 μg/dL in 1920 to 1970 based on a cohort study to compute blood lead across the lifetime. The theoretical minimum-risk exposure level (TMREL) was calculated at 2.0 μg/dL in prior GBD iterations. This limit was determined by a literature review that found no consistent statistically significant estimates of increased relative risks at lower blood lead levels. For GBD 2019, we continued to employ a TMREL of 2.0 μg/dL. While most of the worldwide exposure is thought to be substantially over this threshold, average blood LEs in a number of nations have recently decreased below 2.0 μg/dL. This is in line with pre-industrial blood lead levels in humans, which have been estimated to be as low as 0.018 μg/dL. Blood lead relative risks were previously derived using a pooled analysis from 2005, which was initially used in GBD 2010. Those relative risks were then revised for GBD 2017 based on a 2013 re-analysis of the 2005 paper, yielding slightly altered relative risk estimates unique to exposure at 24 months of age. Since bone lead causes an elevation in SBP, all of the health risks associated with exposure to bone lead were adjusted through SBP. As a result, the relative risks associated with bone LE were identical to those associated with SBP outcomes [5].

### Estimated relevant risk

CVDs including rheumatic heart disease, ischemic heart disease, stroke (ischemic stroke, intracerebral hemorrhage, and subarachnoid hemorrhage), hypertensive heart disease, cardiomyopathy and myocarditis, atrial fibrillation and flutter, aortic aneurysm, peripheral artery disease, endocarditis, non-rheumatic valvular heart disease, and other cardiovascular and circulatory diseases; chronic kidney diseases (CKD) including CKD due to hypertension, diabetes mellitus types 1 one 2, glomerulonephritis, and other and unspecified causes, in addition to idiopathic developmental intellectual disability (IDID) are all associated with the LE (Additional file 1) [5].

### Statistical analysis

We calculated the population attributable fraction (PAF) for bone LE and its associated outcomes using the predefined GBD formula [5, 22]. This equation generated 1000 drawings of the exposure and relative risk models. By multiplying the PAFs with the expected number of fatalities or DALYs for each nation, age, sex, year, and disease, the deaths and DALYs attributed to LE were computed for each country, age, sex, year, and disease. In GBD 2019, the Cause of Death Ensemble model (CODEm) was used to estimate the number of deaths. CODEm creates several different models in order to find the one that best fits all of the available data and variables. YLDs were computed by multiplying the severity-specific disability weights by the prevalence of each severity category for each disease. The number of deaths in each age group was multiplied by the remaining life expectancy of that age group, which was obtained from the GBD standard life table, to create YLLs for each disease. Finally, the YLLs and YLDs were summed to calculate DALYs for each disease [5, 22].

All of the estimates are presented as counts or rates per 100,000, with 95% uncertainty intervals (UIs). The UIs were determined by repeating each computational step 1,000 times and factoring in uncertainty from several sources (e.g., input data and measurement error). The 25th and 975th values of the ordered drawings were used to create the UIs. We also evaluated the burden attributable to LE in different SDI quintiles. The statistical analyses were conducted using R software, version 3.5.2.

## Results

**Fig. 1:**
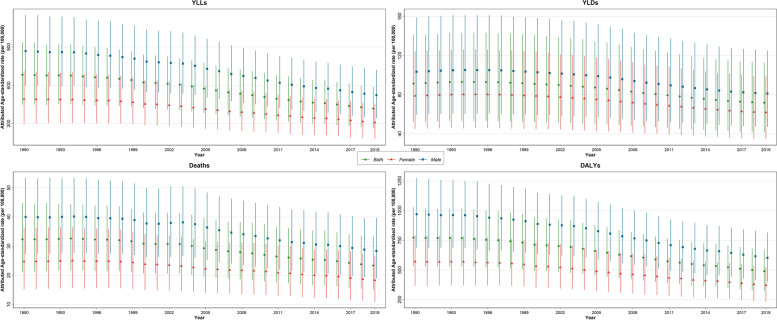
Time trend of age-standardized rate of deaths, disability-adjusted life years (DALYs), years of life lost (YLLs), and years lived with disability (YLDs) attributable to lead exposure in North Africa and Middle East from 1990 to 2019, by sex

In 2019, there were 83.6 thousand deaths (95% UI: 53.2 to 118.1) attributable to LE in the region, with an age-standardized death rate of 23.4 (15.1 to 33.3) per 100,000 (Table 1). There were 52.1 (34.5 to 72.1) thousand deaths in men and 31.6 (18.7 to 46.0) thousand deaths in women in 2019 (Table 1). The age-standardized death rates in 2019 were 28.3 (19.0 to 39.6) and 18.3 (10.9 to 26.7) per 100,000 among men and women, respectively (Table 1). Over 1990–2019, there was a plateau up to early 2000s in terms of age-standardized death rates, while it was decreased thereafter and overall, there was 27.7% decrease between 1990 and 2019 (-35.7% to -19.9%) (Table 1, Fig. 1).

**Table 1:** All-age numbers and age-standardized rates of deaths, disability-adjusted life years (DALYs), years of life lost (YLLs), and years lived with disability (YLDs) of all causes, cardiovascular diseases, chronic kidney diseases, and idiopathic developmental intellectual disability attributable to lead exposure by sex in 1990 and 2019 and overall percent change over 1990–2019 in North Africa and Middle East

Cause	Measure	Age, metric	Year						% Change (1990 to 2019)		
			1990			2019					
			Both	Female	Male	Both	Female	Male	Both	Female	Male
All causes	Deaths	All ages number	48,576 (31,942 to 66,345)	17,962 (11,028 to 25,943)	30,614 (20,980 to 40,617)	83,649 (53,222 to 118,090)	31,595 (18,657 to 45,968)	52,054 (34,450 to 72,130)	72.2 (50.6 to 94.3)	75.9 (51.6 to 98.6)	70 (48.7 to 93)
		Age-standardized rate (per 100,000)	32 (21.2 to 44.7)	24.7 (15.1 to 36.3)	40 (0 to 53.4)	23.4 (15.1 to 33.3)	18.3 (10.9 to 26.7)	28.3 (19 to 39.6)	-27.7 (-35.7 to -19.9)	-26 (-35.2 to -17.3)	-29.1 (-36.7 to -20.8)
	DALYs	All ages number	1,499,300 (1,051,985 to 1,991,356)	540,327 (350,435 to 745,097)	958,974 (684,635 to 1,249,646)	2,143,156 (1,395,425 to 2,956,053)	783,050 (490,057 to 1,115,655)	1,360,106 (904,288 to 1,848,977)	42.9 (24.5 to 61)	44.9 (25.1 to 63.8)	41.8 (23.7 to 60.8)
		Age-standardized rate (per 100,000)	774 (530.7 to 1029.4)	570.9 (364 to 792)	970.4 (0 to 1277.2)	489.3 (320.5 to 669.6)	370.9 (231.5 to 524.8)	603 (403 to 816.1)	-36.8 (-44 to -29.6)	-35 (-43.3 to -27.7)	-37.9 (-44.9 to -30.5)
	YLLs	All ages number	1,208,215 (796,282 to 1,642,371)	418,536 (252,168 to 601,138)	789,678 (541,797 to 1,054,175)	1,745,549 (1,077,970 to 2,474,354)	616,632 (351,943 to 917,908)	1,128,917 (727,933 to 1,580,746)	44.5 (22.7 to 66.5)	47.3 (23.2 to 70)	43 (21.1 to 64.6)
		Age-standardized rate (per 100,000)	682 (448.9 to 933.9)	492.1 (301 to 708.8)	867 (0 to 1148.9)	417.5 (263.3 to 587.2)	309 (180.1 to 452)	521.9 (343.7 to 719.8)	-38.8 (-47 to -30.7)	-37.2 (-46.7 to -28.7)	-39.8 (-47.7 to -31.8)
	YLDs	All ages number	291,086 (156,470 to 470,608)	121,790 (63,726 to 199,842)	169,296 (91,239 to 272,205)	397,607 (217,577 to 636,484)	166,418 (90,399 to 271,164)	231,189 (127,512 to 369,176)	36.6 (28.4 to 49.9)	36.6 (27.6 to 51)	36.6 (28 to 49.9)
		Age-standardized rate (per 100,000)	91 (51.9 to 141.1)	78.8 (45 to 124.2)	103.4 (0 to 159.2)	71.8 (41.6 to 112.6)	61.9 (34.7 to 98.7)	81.1 (47 to 125)	-21.5 (-25 to -17.5)	-21.4 (-25.5 to -17.8)	-21.6 (-25.5 to -17.5)
CVDs	Deaths	All ages number	46,310 (30,539 to 63,295)	17,047 (10,430 to 24,685)	29,264 (20,040 to 38,958)	78,639 (49,885 to 111,506)	29,529 (17,372 to 43,196)	49,111 (32,267 to 68,634)	69.8 (48.1 to 92.3)	73.2 (48.9 to 97.7)	67.8 (46.1 to 91.2)
		Age-standardized rate (per 100,000)	31 (20 to 42.5)	23.4 (14.3 to 34.4)	38 (0 to 51.2)	21.9 (14 to 31.4)	17.1 (10.2 to 25)	26.6 (17.6 to 37.2)	-28.7 (-36.6 to -20.5)	-27.1 (-36.1 to -18.1)	-30 (-37.6 to -21.5)
	DALYs	All ages number	1,203,514 (792,192 to 1,636,161)	417,815 (251,251 to 598,772)	785,699 (536,437 to 1,050,240)	1,739,574 (1,071,014 to 2,468,693)	614,305 (348,097 to 918,787)	1,125,269 (714,798 to 1,580,411)	44.5 (23.3 to 65.7)	47 (23.3 to 69.3)	43.2 (21.9 to 64.8)
		Age-standardized rate (per 100,000)	677 (445 to 921.6)	489.4 (299.5 to 704.8)	859 (0 to 1143.8)	414.2 (258.6 to 580.6)	306.4 (178.2 to 449.8)	517.8 (337.9 to 719)	-38.8 (-46.9 to -30.9)	-37.4 (-46.7 to -29)	-39.7 (-47.6 to -31.9)
	YLLs	All ages number	1,157,744 (763,354 to 1,583,670)	398,382 (240,254 to 573,827)	759,362 (520,673 to 1,019,796)	1,650,968 (1,017,507 to 2,359,734)	577,871 (327,807 to 861,602)	1,073,097 (690,884 to 1,508,458)	42.6 (21.4 to 64.3)	45.1 (20.6 to 68)	41.3 (19.8 to 63.3)
		Age-standardized rate (per 100,000)	652 (429 to 894.7)	467.6 (285.8 to 675.9)	830.4 (0 to 1105.9)	393.5 (246.2 to 553.7)	289.1 (167.3 to 424.2)	493.9 (321.3 to 687.3)	-39.6 (-47.8 to -31.4)	-38.2 (-47.7 to -29.4)	-40.5 (-48.6 to -32.5)
	YLDs	All ages number	45,770 (26,420 to 69,901)	19,432 (9,963 to 30,905)	26,338 (16,143 to 38,846)	88,606 (47,738 to 140,414)	36,434 (17,496 to 61,098)	52,172 (29,957 to 79,849)	93.6 (75.5 to 107.2)	87.5 (66.4 to 102.6)	98.1 (79.8 to 112.3)
		Age-standardized rate (per 100,000)	25 (14.6 to 38)	21.7 (11.5 to 34.2)	28.6 (0 to 41.9)	20.6 (11.4 to 32.2)	17.3 (8.6 to 28.5)	23.9 (14.2 to 36.3)	-18.1 (-24.3 to -13.5)	-20.5 (-28.1 to -15.2)	-16.5 (-22.5 to -11.8)
CKDs	Deaths	All ages number	2,266 (1,431 to 3,310)	916 (533 to 1,422)	1,350 (880 to 2,009)	5,010 (3,157 to 7,279)	2,067 (1,156 to 3,114)	2,944 (1,940 to 4,264)	121.1 (82 to 164)	125.7 (71.7 to 169.9)	118 (74.3 to 176.6)
		Age-standardized rate (per 100,000)	2 (1 to 2.4)	1.3 (0.7 to 2)	2 (0 to 3)	1.5 (0.9 to 2.1)	1.2 (0.7 to 1.8)	1.7 (1.2 to 2.5)	-9.6 (-26.4 to 8.2)	-6.5 (-30.4 to 11.8)	-12.8 (-30.8 to 10.1)
	DALYs	All ages number	56,954 (36,279 to 81,987)	23,309 (13,376 to 36,048)	33,645 (22,258 to 48,701)	113,721 (68,001 to 168,320)	46,735 (24,208 to 72,554)	66,986 (42,336 to 98,574)	99.7 (64.9 to 133.4)	100.5 (54.8 to 136.4)	99.1 (62.6 to 144)
		Age-standardized rate (per 100,000)	34 (21.9 to 49.2)	28.1 (16.3 to 43)	40.3 (0 to 58.8)	28.7 (17.7 to 41.7)	23.9 (13 to 36.3)	33.5 (21.8 to 48.7)	-15.9 (-30.2 to -2.2)	-15 (-34.3 to -0.3)	-17 (-32.5 to 2.3)
	YLLs	All ages number	50,470 (31,753 to 73,350)	20,154 (11,533 to 31,127)	30,316 (19,984 to 43,954)	94,581 (56,799 to 141,152)	38,761 (20,426 to 60,766)	55,820 (35,499 to 82,649)	87.4 (52.2 to 125)	92.3 (43.9 to 133.2)	84.1 (45.9 to 134)
		Age-standardized rate (per 100,000)	31 (19.3 to 44.2)	24.4 (14.2 to 37.2)	36.6 (0 to 54)	24 (14.8 to 34.9)	19.9 (10.9 to 30.5)	28 (18.1 to 40.9)	-21.3 (-35.9 to -5.6)	-18.6 (-38.4 to -2.3)	-23.4 (-39.1 to -2.6)
	YLDs	All ages number	6,484 (3,533 to 10,676)	3,155 (1,574 to 5,338)	3,329 (1,911 to 5,218)	19,140 (9,908 to 32,496)	7,974 (3,765 to 14,386)	11,166 (6,123 to 18,499)	195.2 (165.1 to 223.2)	152.8 (124.4 to 176.1)	235.4 (197.4 to 271.9)
		Age-standardized rate (per 100,000)	4 (2.1 to 6)	3.7 (1.9 to 6.1)	3.7 (0 to 5.8)	4.7 (2.6 to 7.8)	4 (2 to 6.9)	5.5 (3 to 8.8)	27.8 (16.4 to 39.3)	9 (-1.5 to 18.1)	46.1 (31.5 to 60.8)
IDID	YLDs	All ages number	238,832 (108,495 to 412,312)	99,204 (43,668 to 173,964)	139,629 (64,540 to 239,163)	289,861 (129,611 to 513,759)	122,010 (53,947 to 219,279)	167,851 (72,972 to 295,680)	21.4 (13 to 26.7)	23 (15.1 to 28.7)	20.2 (10.4 to 26.5)
		Age-standardized rate (per 100,000)	63 (28.3 to 107.9)	53.4 (23.5 to 93.7)	71.1 (0 to 122)	46.4 (20.7 to 82.1)	40.6 (18 to 73)	51.7 (22.5 to 91.1)	-25.7 (-30.8 to -22.5)	-23.9 (-28.9 to -20.4)	-27.2 (-33 to -23.6)

Data in parenthesis are 95% uncertainty intervals

*CVDs* Cardiovascular Diseases, *CKDs* Chronic Kidney Diseases, *IDID* Idiopathic Developmental Intellectual Disability, *DALYs* Disability-Adjusted Life Years, *YLLs* Years of Life Lost, *YLDs* Years Lived with Disability

During 2019, LE caused 2.1 million DALYs (1.4 to 3.0), with 1.4 (0.9 to 1.8) million DALYs in men and 783.1 (490.1 to 1115.7) thousand DALYs in women (Table 1). It was attributable to 489.3 (320.5 to 669.6) age-standardized DALY rate per 100,000 (603.0 [403.0 to 816.1] and 370.9 [231.5 to 524.8] among men and women, respectively). Between 1990 and 2019, the age-standardized DALY rate associated with LE (per 100,000) decreased by 36.8% (-44.0% to -29.6%) (Table 1, Fig. 1).

**Fig. 2:**
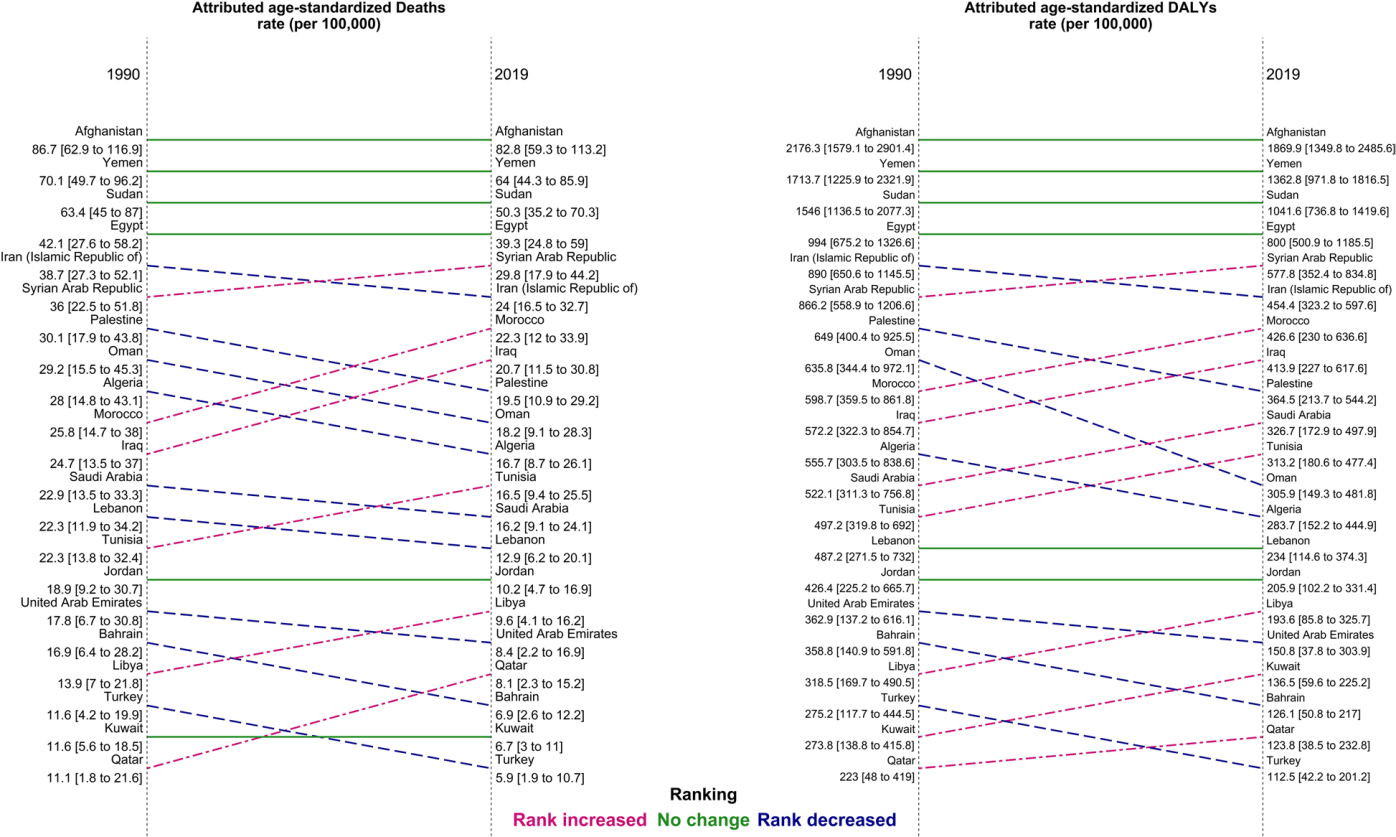
Comparison the ranking of age-standardized rate of deaths and disability-adjusted life years (DALYs) attributable to lead exposure in North Africa and Middle East countries for both sexes between 1990 and 2019

The age-standardized death rates attributable to LE were 7.8 times higher in the country with the highest death rate than that with the lowest one in 1990, while the difference increased to 14.0 times in 2019. In 2019, Afghanistan (82.8 [59.3 to 113.2]), Yemen (64.0 [44.3 to 85.9]) and Sudan (50.3 [35.2 to 70.3]) had the three highest age-standardized death rates attributable to LE per 100,000. On the other hand, the lowest rates were found in Turkey (5.9 [1.9 to 10.7]), Kuwait (6.7 [3.0 to 11.0]) and Bahrain (6.9 [2.6 to 12.2]) (Fig. 2 and Table S1). All countries witnessed a decrease in the age-standardized death rate between 1990 and 2019. Moreover, between 1990 and 2019, Bahrain (-59.3% [-66.7% to -49.0%]), the United Arab Emirates (-52.8% [-69.7% to -39.0%]), and Turkey (-49.4% [-61.3% to -37.4%]) had the highest decrease in age-standardized death rates attributable to LE, while Afghanistan (-4.5% [-25.0% to 16.8%]), Egypt (-6.6% [-26.1% to 15.5%]), and Yemen (-8.8% [-28.1% to 18.8%]) had the lowest ones (Table S1). Afghanistan had the highest age-standardized death rates for men (91.4 [65.4 to 121.3]) and women (74.9 [52.5 to 104.3]) per 100,000. The lowest rates for men were in Turkey (7.1 [2.6 to 12.5]), while Kuwait had the lowest one for women (3.7 [1.3 to 6.9]) (Additional file 4: Figure S1 and Additional file 5: Figure S2).

The age-standardized DALY rates attributable to LE were 9.8 times higher in the country with the highest DALY rate than that with the lowest one in 1990, while the difference increased to 16.6 times in 2019. Afghanistan (1869.9 [1349.8 to 2485.6]), Yemen (1362.8 [971.8 to 1816.5]) and Sudan (1041.6 [736.8 to 1419.6]) had the three highest age-standardized DALY rates. In contrast, the lowest rates were represented in Turkey (112.5 [42.2 to 201.2]), Qatar (123.8 [38.5 to 232.8]), and Bahrain (126.1 [50.8 to 217.0]) (Fig. 2, Table S1). The age-standardized DALY rates decreased in all 21 countries in the NAME region between 1990 and 2019. Furthermore, in this time, the percent change in age-standardized DALY rates had the highest decrease in Bahrain (-64.9% [-71.4% to -56.3%]), Turkey (-59.1% [-68.9% to -50.2%]), and the United Arab Emirates (-58.4% [-74.8% to -45.0%]), while Afghanistan (-14.1% [-33.2% to 7.5%]), Egypt (-19.5% [-36.6% to -0.2%]), and Yemen (-20.5% [-37.5% to 3.7%]) had the lowest ones (Table S1). By sex, Afghanistan had the highest age-standardized DALY rates for men (2089.8 [1510.0 to 2721.3]) and women (1669.0 [1179.0 to 2278.3]) per 100,000. The lowest rates for men were in Qatar (121.1 [41.3 to 224.0]), while Kuwait had the lowest one for women (73.9 [27.3 to 130.5]) (Figures S1, S2). The geographical distribution of age-standardized YLLs, YLDs, death, and DALY rates were higher in countries located in the northeast of North Africa and east of Middle East in 1990 and 2019 for both sexes (Figure S3). There was a similar geographical distribution pattern of the measures in NAME in 1990 and 2019 for men (Figure S4) and women (Figure S5). The time trend of age-standardized death and DALY rates from 1990 and 2019 was decreasing in almost all countries, except for Afghanistan and Egypt which showed a peak in early 2000s (Figure S6).

**Fig. 3:**
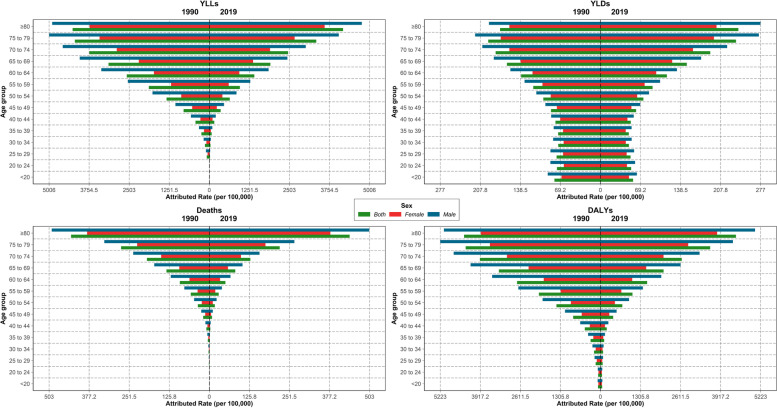
Rate of deaths, disability-adjusted life years (DALYs), years of life lost (YLLs), and years lived with disability (YLDs) attributable to lead exposure in North Africa and Middle East in 1990 and 2019, by sex and age

In 1990, the highest death and DALY rates attributable to LE were in the 80 + age group. Similarly, the attributable death and DALY rates (per 100,000) increased with advancing age up to 80 + age group in both sexes in 2019 in the region. Men had higher attributable death and DALYs in all age groups in 1990 and 2019 than women (Fig. 3). Although most countries in the NAME had similar age and sex patterns in terms of attributable death and DALY rates, countries like Qatar had higher attributable death and DALY rates in women than men (Additional file 2).

**Fig. 4:**
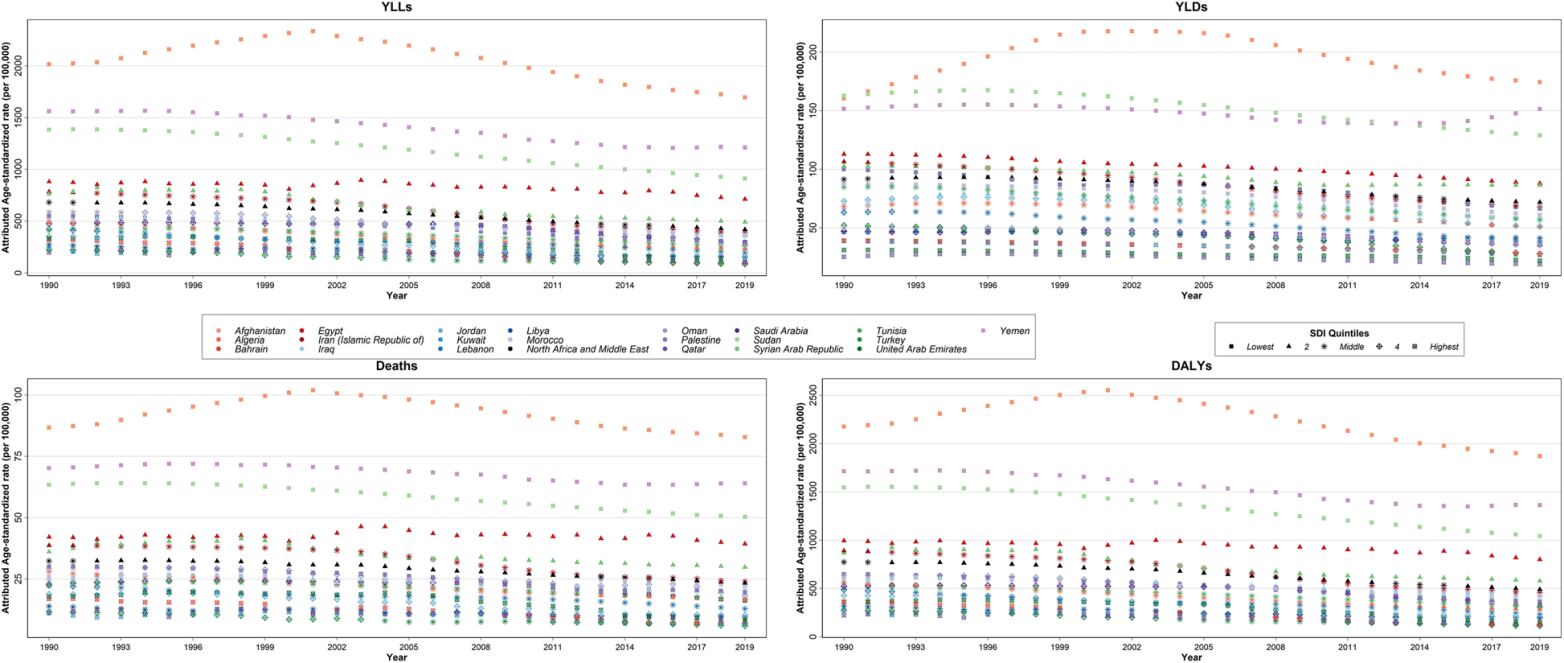
Time trend of age-standardized rate of deaths, disability-adjusted life years (DALYs), years of life lost (YLLs), and years lived with disability (YLDs) attributable to lead exposure in North Africa and Middle East region and its 21 countries from 1990 to 2019, by sociodemographic index (SDI) quintiles

The high SDI countries had lower attributable burden compared with low SDI quintile (Figure S7). Also, the attributable death and DALY rates had a decreasing trend in countries of all of the SDI quintiles over 1990–2019 (Fig. 4).

**Fig. 5:**
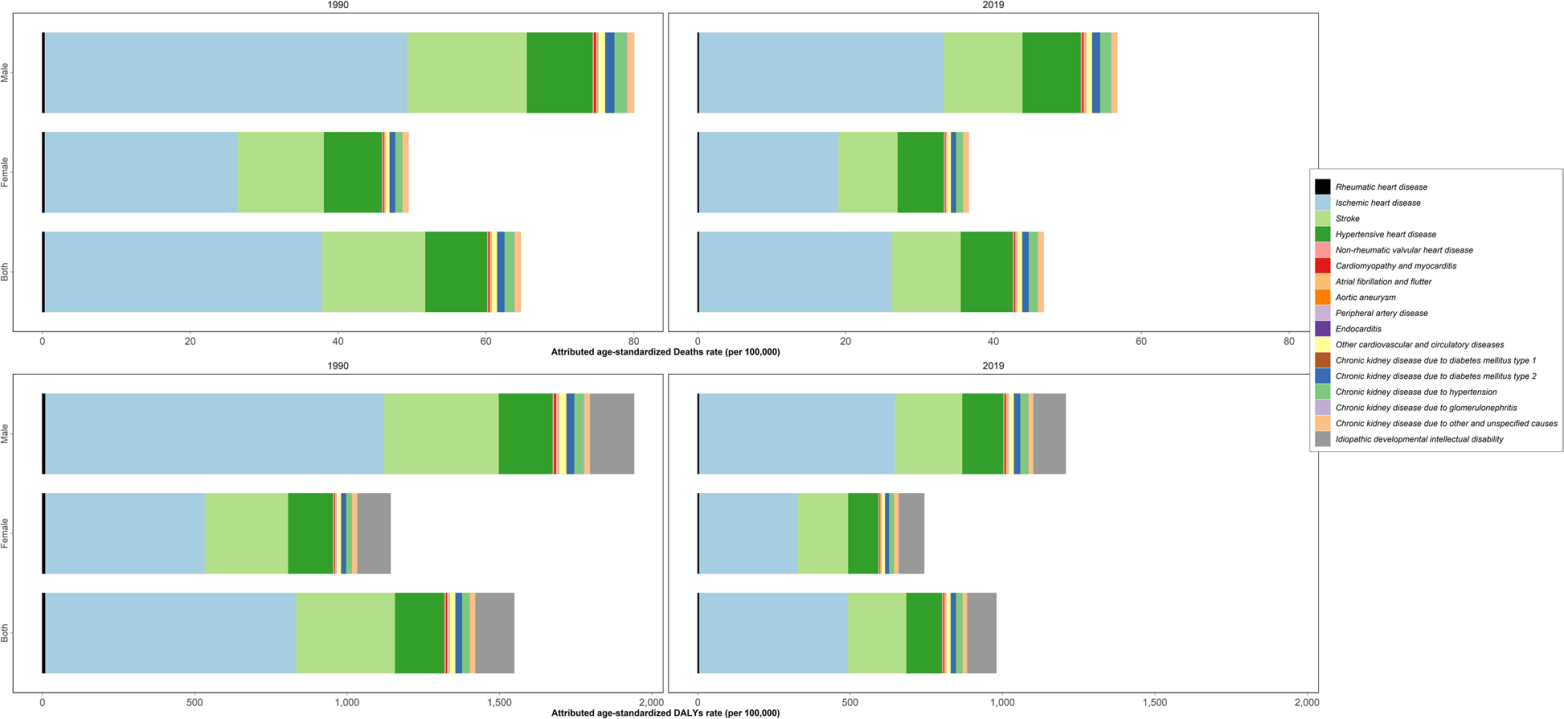
Age-standardized rate of deaths and disability-adjusted life years (DALYs) of underlying causes attributable to lead exposure in North Africa and Middle East in 1990 and 2019

The highest attributable age-standardized death rate of LE came from CVDs (21.9 [14.0 to 31.4]), followed by CKDs (1.5 [0.9 to 2.1]) per 100,000 (Table 1). Moreover, CVDs (414.2 [258.6 to 580.6]) and CKDs (28.7 [17.7 to 41.7]) accounted for the attributable DALY rates to LE in 2019 (Table 1). The attributable age-standardized YLDs rate for IDID decreased from 63.0 (28.3 to 107.9) in 1990 to 46.4 (20.7 to 82.1) per 100,000, representing 25.7% (-30.8 to -22.5%) decrease over 1990–2019 (Table 1). The highest LE-attributable age-standardized death rates were due to ischemic heart disease, stroke, and hypertensive heart disease. Additionally, the highest attributable age-standardized DALY rates were due to ischemic heart disease, stroke, hypertensive heart disease, and IDID (Fig. 5).

## Discussion

There was a 36.8% reduction in the age-standardized DALY rate attributable to LE in the NAME region between 1990 and 2019. The attributed age-standardized DALY rates in 2019 ranged from 112.5 to 1869.9, with Afghanistan, Yemen, and Sudan responsible for the highest rates. In contrast, the lowest ones were found in Turkey, Qatar, and Bahrain. Also, Bahrain, the United Arab Emirates, and Turkey had the highest decrease in age-standardized death and DALY rates attributable to LE, while Afghanistan, Egypt, and Yemen had the lowest ones over 1990–2019. Moreover, the difference between the country with the highest DALYs and that with the lowest one increased from 9.8 times in 1990 to 16.6 times in 2019. The geographical distribution of burdens attributable to LE was higher in countries located in the northeast of North Africa and east of Middle East in 1990 and 2019 for both sexes. Consistently, it has been revealed that high SDI countries had lower attributable burden compared with counties within the low SDI quintile.

The high levels of LE and its adverse impacts on human health have become a significant public health concern in the 1990s. Therefore, increasing attention was being accorded to reducing LE [23]. Although the results of the GBD 2019 study showed that the overall LE declined approximately 1% per year [5], the reduction in LE occurred slower in LMICs and developing countries in comparison to high-income countries, thereby LE still remains higher in LMICs [3]. The largest burden of LE was born by LMICs, and 1.20% of world gross domestic product (GDP) in 2011 was related to these countries [24]. It is estimated that in 2019, LE accounted for 900,000 deaths and 21.7 million DALYs worldwide, with the highest burden in LMICs [25]. Bret Ericson et al. aimed to evaluate the blood lead concentration in LMICs. Results of this study show that mean blood lead levels had a broad range from 1.66 µg/dL in Ethiopia to 9.30 µg/dL in Palestine in children, as well as from 0.39 µg/dL in Sudan to 11.36 µg/dL in Pakistan in adults. Furthermore, most people had blood lead concentration exceeding 5 µg/dL [6], which is higher than reference value of 3.5 µg/dL for children and 5 µg/dL for adults used by the CDC [7]. Additionally, a study found that pregnant women living in particular sites, known as Toxic Sites Identification Program (TSIP), in LMICs were highly at risk of LE leading to blood lead levels above the standard value, that their fetuses were put at risk for neurologic and other sequelae [26].

The DALY rates showed a positive association with advancing age up to 80 + in both sexes, and it was more significant in men in all age groups, both in 1990 and 2019. It could imply that the elderly population are more susceptible to ischemic heart disease, stroke, hypertensive heart disease, impaired renal function, and decline in cognitive function, which are associated with the high attributable DALYs and deaths to LE [27]. However, young children are particularly vulnerable to profound and permanent adverse toxic effects of lead on the brain and nervous system development. Lead also causes long-term harm in adults, including an increased risk of high blood pressure and kidney damage [8]. Also, the attributable age-standardized death rate and DALY were higher among men than women, which may refer to occupational differences between men and women [28].

This study found that the highest attributable age-standardized death and DALY rates of LE came from CVDs, particularly ischemic heart disease, stroke, and hypertensive heart disease, followed by CKDs in 2019. The attributable age-standardized YLD rate for IDID remains 46.4, which in GBD 2019, IDID was the only cause of mental disorders estimated for the lead exposure. The overall estimations of GBD 2019 demonstrated that LE accounted for 62.5%, 8.2%, 7.2%, and 5.65% of the global burden of IDID, hypertensive heart disease, ischemic heart disease, and stroke, respectively [25]. Previously, the burden of mental retardation and cardiovascular adverse outcomes resulting from LE were estimated almost 1% of the global burden of disease, with the highest rate in developing countries [29]. A large proportion, approximately 62.8%, of estimated DALYs for IDID based on GBD 2017 was attributable to LE in India [30].

It should be noted that there was no low dose threshold for appearing adverse effects, as the decrements in IQ and increased risk of cardiovascular morbidity and mortality are even seen at a level as low as one µg/dL [15, 16, 31, 32]. A study on TSIP in seven Asian countries showed that diminished intelligence ranging from 4.94 to 14.96 IQ scores could occur due to elevated LE, even in blood lead levels below 10 µg/dL [33]. Another international study concluded that blood lead levels < 7.5 µg/dL could cause intellectual deficits in children [9]. A systematic review study revealed the positive relationship between LE and coronary heart disease, stroke, and peripheral arterial disease that could be observed at blood lead levels < 5 µg/dL [14]. Besides, it has been reported that the odds of CVDs and diabetic kidney disease and blood lead levels moved in the same direction in middle-aged and elderly diabetic adults [34]. The greater blood lead levels are correlated with mortality and morbidity from CKDs, which is apparent in the range of blood lead levels below 10 μg/dl [35]. Environmental exposure to lead, even at a low level, could accelerate progressive renal insufficiency in CKD patients [36, 37]. Furthermore, it should be noted that the DALYs attributable to LE are more considerable in low–middle SDI regions than in global and high SDI regions [5].

Since CKD, CVDs, and mental disorders, account for a large proportion of DALYs [33, 38–40] and impose a high economic burden [41], particularly in the NAME region and LMICs, the preventive strategies against related risk factors, such as LE, seem to be cost-effective to reduce the disease burden. Also, these countries have the highest burden of diseases attributable to LE [42]. Besides, it has been reported that the application of governmental actions and public health efforts for regulation of lead content and control of lead sources successfully contributed to the reduction in LE, in the United States in 2019 [43]. Due to the difference in sources, causes, and patterns of LE between high-income countries and LMICs, the LE reduction programs developed in high-income countries for more universal sources of lead, for example, paint and gasoline, are not fully translatable to the exposure context in LMICs [42, 44–46]. Informal acid battery manufacture and recycling, mining, metal processing, and electronic waste were reported as the primary sources of LE in LMICs [6, 47, 48]. A recent study conducted in Iran demonstrated that the average drinking water concentration of lead was 2.5 μg/L, much lower than the standard values. Also, the LE was responsible for 0.2% of the attributable burden of disease resulting from heavy metals in drinking water [49].

Additionally, inadequate introduced policies, regulations, and observation on the informality of many industries and improper disposal of contaminants could be noted as reasons of higher LE in LMICs, which can put the population at risk of several adverse health outcomes [47, 50]. Nevertheless, the LMICs are at higher risk of LE, and limited exposure data from each country are available. As it has been reported that adequate data about blood lead levels are available from only 44 countries of the 137 countries classified as LMICs by the World Bank [6]. Also, there is limited data concerning the best method for LE reduction in the region, it has been reported that a mixed approach, focusing on education and decreased poverty, should be considered [42]. It should be nothed that dangerous LE could be generated by rapid industrialization; therefore, national and international efforts in combination with local government programs are required to design the proper preventive measures against LE [3, 51].

Regarding the strengths, this study provides the most comprehensive and up-to-date information about the current situation and trends of the attributable burden to LE and its attruibutable diseases, including CKD, CVDs, and mental disorders, based on the data from GBD 2019 in the NAME region during 1990–2019. Moreover, like other GBD studies, the major limitations of the present study resulted from GBD methodologies in data collection and usage of the the complex modelling strategies, so we cannot manipulate them. The availability and quality of primary data, which are foundation of the GBD analysis is the main limitation of GBD estimates, particularly in regions with countries that have poor completeness rates of the data sources. Where data are not available, the results depend on the out-of-sample predictive validity of the modeling efforts. In addition, drawbacks of exposure measurement of the GBD 2019 study, such as heterogeneous patterns of data availability and less reliable methods of data collection in different regions over time, apply to our results [5, 22].

## Conclusions

Age-standardized DALY and death rates attributed to LE decreased 36.8% and 27.7%, respectively, over 1990–2019 in the region, and both are reversely associated with SDI. Also, we found that the highest LE attributable age-standardized DALYs were from ischemic heart disease, stroke, hypertensive heart disease, and IDID. Given the higher LE in the NAME region and adverse health impacts of lead, even at low amounts of exposure, urgent attention and measures are required to control and reduce the source of LE and its attributable burden. In addition, the lack of reliable exposure data from each country of the NAME region underscores the need for more studies to determine the exact burden of disease attributed to LE in this region.

## Supplementary Information

**Additional file 1. **Global burden of disease classification for levels of environmental risk factors, their attributable causes, and the different causes attributable to lead exposure.

**Additional file 2. **Rate of deaths, disability-adjusted life years (DALYs), years of life lost (YLLs), and years lived with disability (YLDs) attributable to lead exposure in 21 countries of North Africa and Middle East region in 1990 and 2019, by sex and age.

**Additional file 3: Table S1.** All-age numbers and age-standardized rates of deaths, disability-adjusted-life-years (DALYs), years of life lost (YLLs), and years lived with disability (YLDs) attributable to lead exposure in 1990 and 2019 and overall percent change over 1990-2019 in North Africa and Middle East, by country.

**Additional file 4: Figure S1.** Ranking of the age-standardized rate of deaths and disability-adjusted life years (DALYs) attributable to lead exposure in North Africa and Middle East countries for men between 1990 and 2019.

**Additional file 5: Figure S2.** Ranking of the age-standardized rate of deaths and disability-adjusted life years (DALYs) attributable to lead exposure in North Africa and Middle East countries for women between 1990 and 2019.

**Additional file 6: Figure S3.** Geographical distribution of age-standardized rate of deaths, disability-adjusted life years (DALYs), years of life lost (YLLs), and years lived with disability (YLDs) attributable to lead exposure among both sexes in North Africa and Middle East countries in 1990 and 2019.

**Additional file 7: Figure S4. **Geographical distribution of age-standardized rate of deaths, disability-adjusted life years (DALYs), years of life lost (YLLs), and years lived with disability (YLDs) attributable to lead exposure among men in North Africa and Middle East countries in 1990 and 2019.

**Additional file 8: Figure S5.** Geographical distribution of age-standardized rate of deaths, disability-adjusted life years (DALYs), years of life lost (YLLs), and years lived with disability (YLDs) attributable to lead exposure among women in North Africa and Middle East countries in 1990 and 2019.

**Additional file 9: Figure S6.** Time trend of age-standardized rate of deaths, disability-adjusted life years (DALYs), years of life lost (YLLs), and years lived with disability (YLDs) attributable to lead exposure in North Africa and Middle East region and 21 countries from 1990 to 2019.

**Additional file 10: Figure S7.** Age-standardized rate of deaths, disability-adjusted life years (DALYs), years of life lost (YLLs), and years lived with disability (YLDs) attributable to lead exposure in 21 countries of North Africa and Middle East region in 1990 and 2019, by sociodemographic index (SDI) quintiles.

## Data Availability

The database used during the current study is available in http://ghdx.healthdata.org/gbd-results-tool.

## Abbreviations

LE: Lead exposure
DALYs: Disability-adjusted life years
SDI: Socio-demographic index
LMICs: Low- and middle-income countries
NAME: North Africa and Middle East
GBD: Global Burden of Disease
YLDs: Years lived with disability
YLLs: Years of life lost
CDC: Centers for Disease Control and Prevention
WHO: World Health Organization
CVDs: Cardiovascular diseases
SBP: Systolic blood pressure
DisMod-MR: Age-integrating Bayesian hierarchal modelling
ST-GPR: Spatiotemporal Gaussian process regression
TMREL: Theoretical minimum-risk exposure level
CKD: Chronic kidney disease
IDID: Idiopathic developmental intellectual disability
PAF: Population attributable fraction
CODEm: Cause of Death Ensemble model
UI: Uncertainty interval
TSIP: Toxic Sites Identification Program
IQ: Intelligence quotient
