# Supplementary material for: Estimating the burden of diseases attributable to lead exposure in the North Africa and Middle East region, 1990–2019: a systematic analysis for the Global Burden of Disease study 2019
Source: Environ Health. 2022 Oct 29;21:105. doi: 10.1186/s12940-022-00914-3 (PMC9617306; doi:10.1186/s12940-022-00914-3)
Supplement: Supplementary file 2 — Additional file 2. Rate of deaths, disability-adjusted life years (DALYs), years of life lost (YLLs), and years lived with disability (YLDs) attributable to lead exposure in 21 countries of North Africa and Middle East region in 1990 and 2019, by sex and age. [file 12940_2022_914_MOESM2_ESM.pdf]

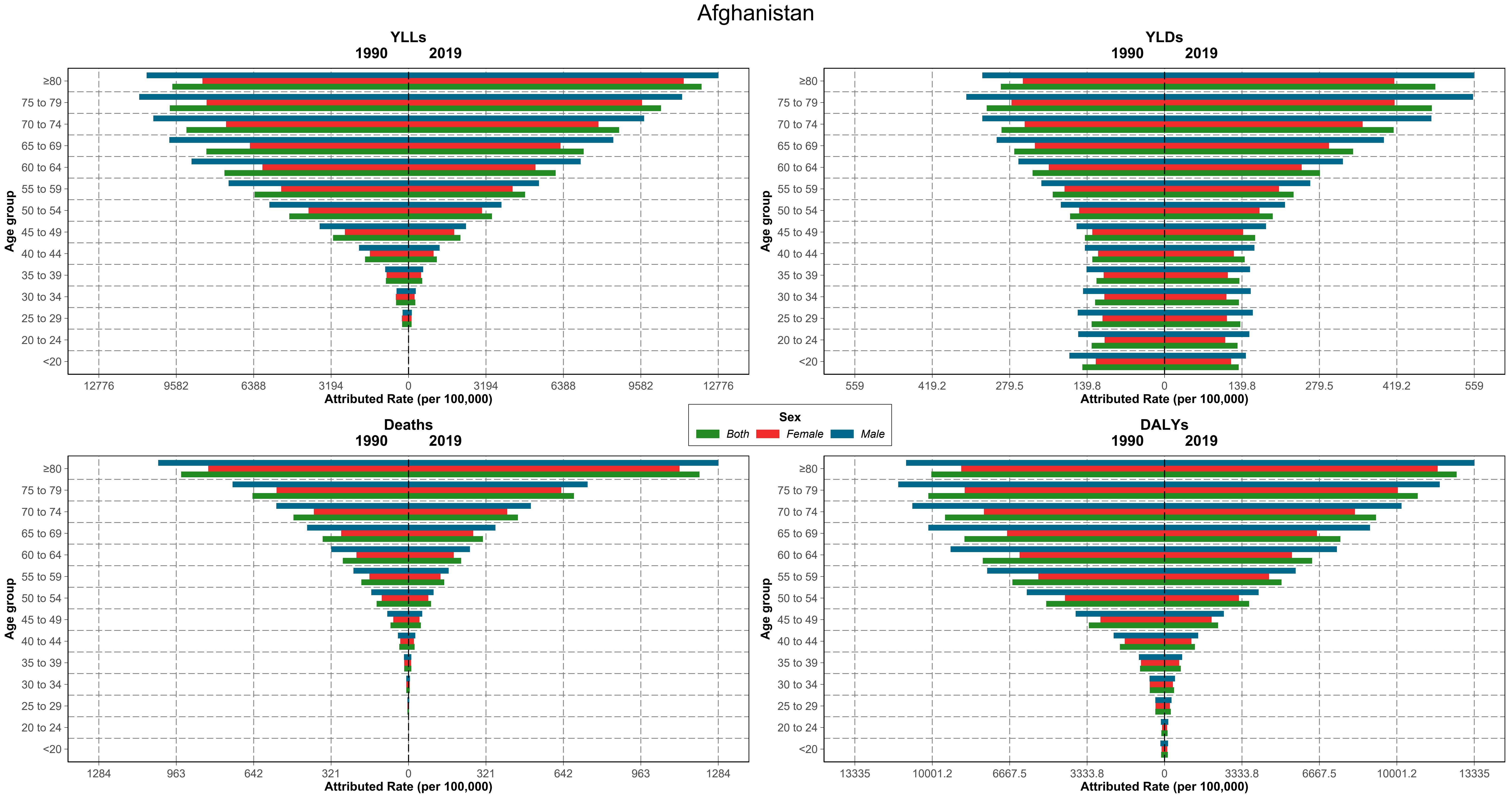
Afghanistan
YLLs
1990
2019
≥80
75 to 79
70 to 74
65 to 69
60 to 64
55 to 59
50 to 54
45 to 49
40 to 44
35 to 39
30 to 34
25 to 29
20 to 24
<20
Age group
12776
9582
6388
3194
0
3194
6388
9582
12776
Attributed Rate (per 100,000)
YLDs
1990
2019
559
419.2
279.5
139.8
0
139.8
279.5
419.2
559
Attributed Rate (per 100,000)
Sex
Both
Female
Male
Deaths
1990
2019
1284
963
642
321
0
321
642
963
1284
Attributed Rate (per 100,000)
DALYs
1990
2019
13335
10001.2
6667.5
3333.8
0
3333.8
6667.5
10001.2
13335
Attributed Rate (per 100,000)

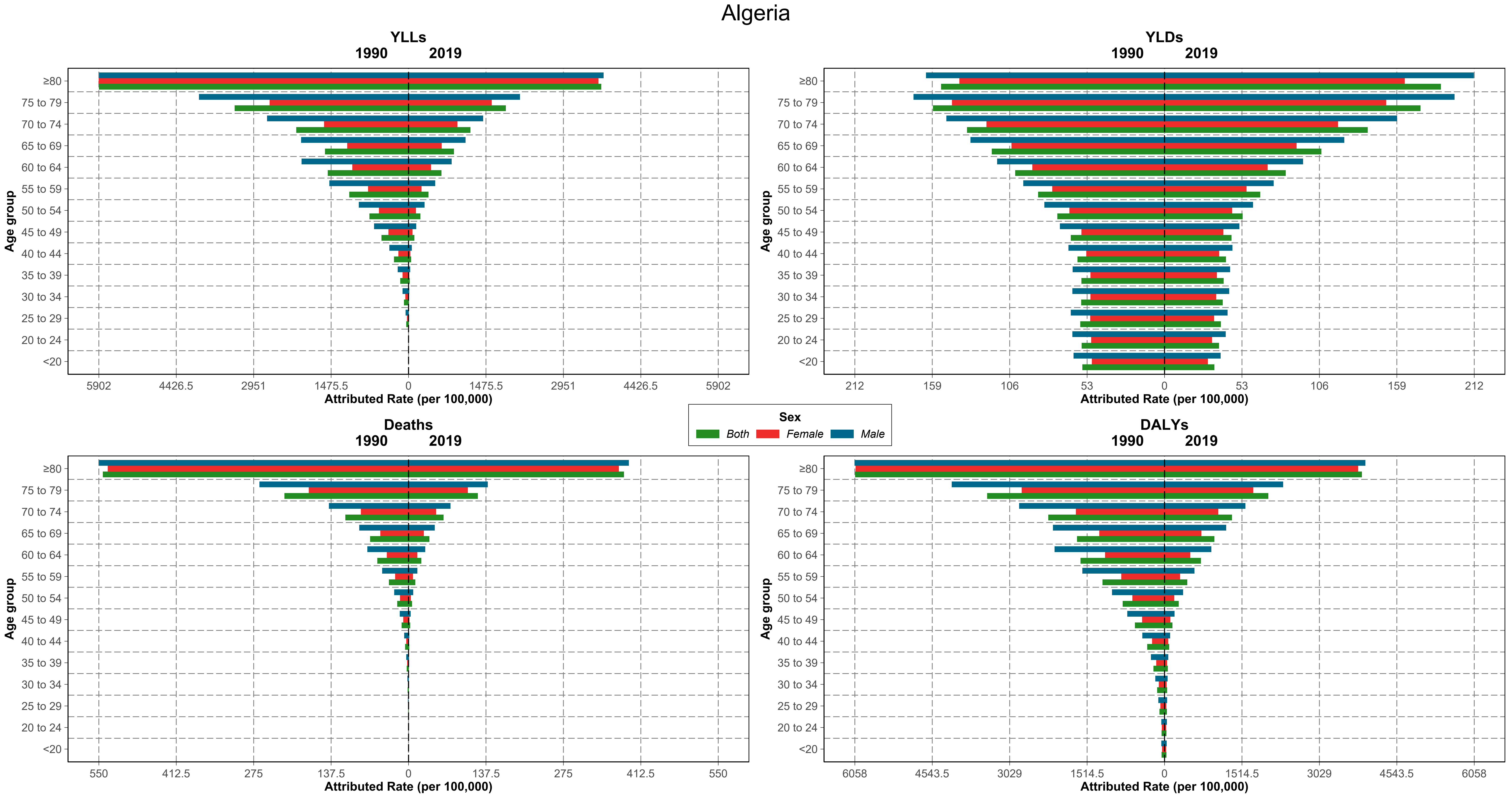

Algeria
YLLs
1990
2019
YLDs
1990
2019
Deaths
1990
2019
DALYs
1990
2019
Sex
Both
Female
Male
Age group
≥80
75 to 79
70 to 74
65 to 69
60 to 64
55 to 59
50 to 54
45 to 49
40 to 44
35 to 39
30 to 34
25 to 29
20 to 24
<20
Attributed Rate (per 100,000)
5902 4426.5 2951 1475.5 0 1475.5 2951 4426.5 5902
212 159 106 53 0 53 106 159 212
550 412.5 275 137.5 0 137.5 275 412.5 550
6058 4543.5 3029 1514.5 0 1514.5 3029 4543.5 6058

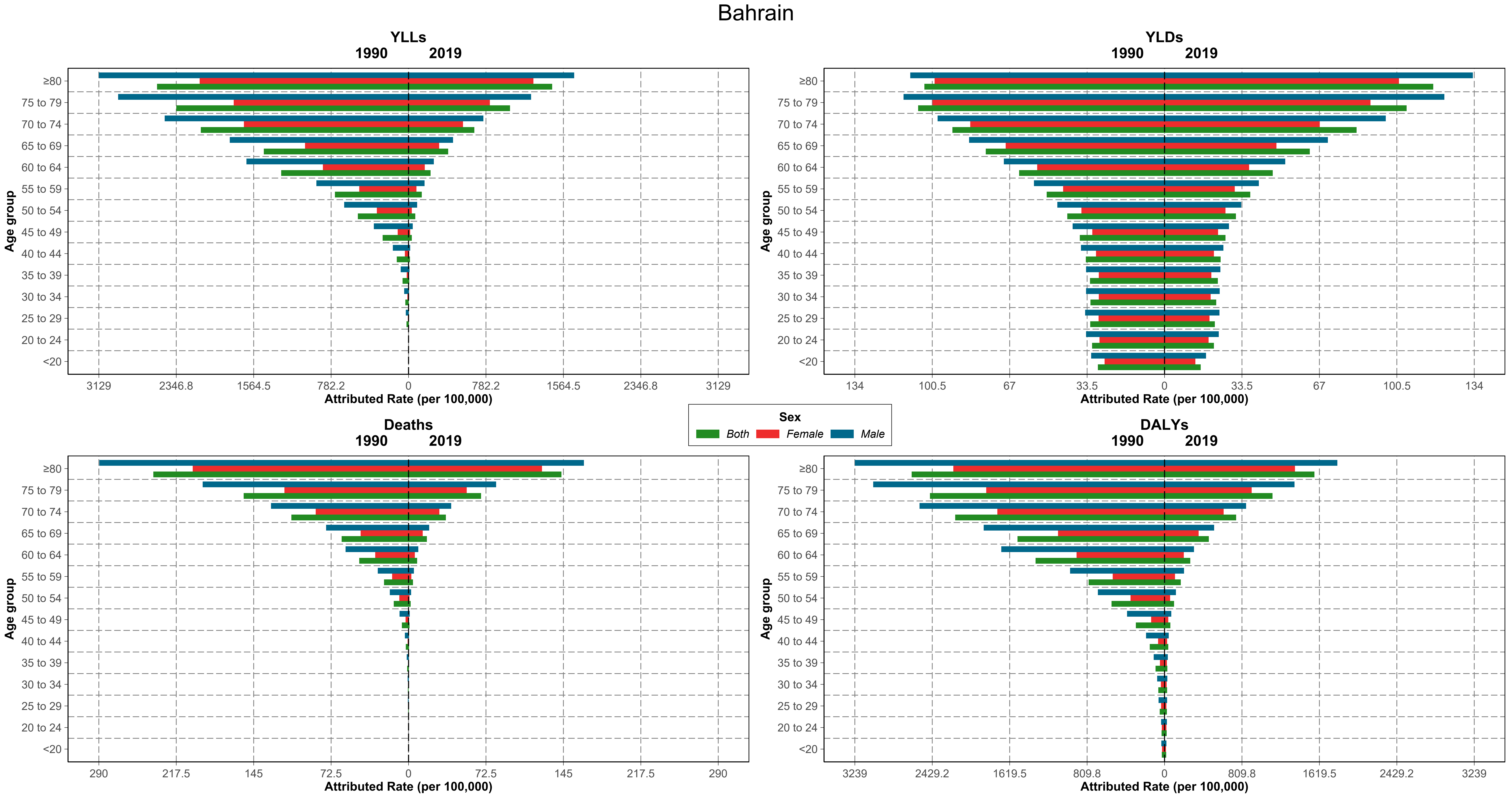

Bahrain
YLLs
1990
2019
YLDs
Deaths
DALYs
Age group
≥80
75 to 79
70 to 74
65 to 69
60 to 64
55 to 59
50 to 54
45 to 49
40 to 44
35 to 39
30 to 34
25 to 29
20 to 24
<20
3129 2346.8 1564.5 782.2 0 782.2 1564.5 2346.8 3129
134 100.5 67 33.5 0 33.5 67 100.5 134
290 217.5 145 72.5 0 72.5 145 217.5 290
3239 2429.2 1619.5 809.8 0 809.8 1619.5 2429.2 3239
Attributed Rate (per 100,000)
Sex
Both
Female
Male

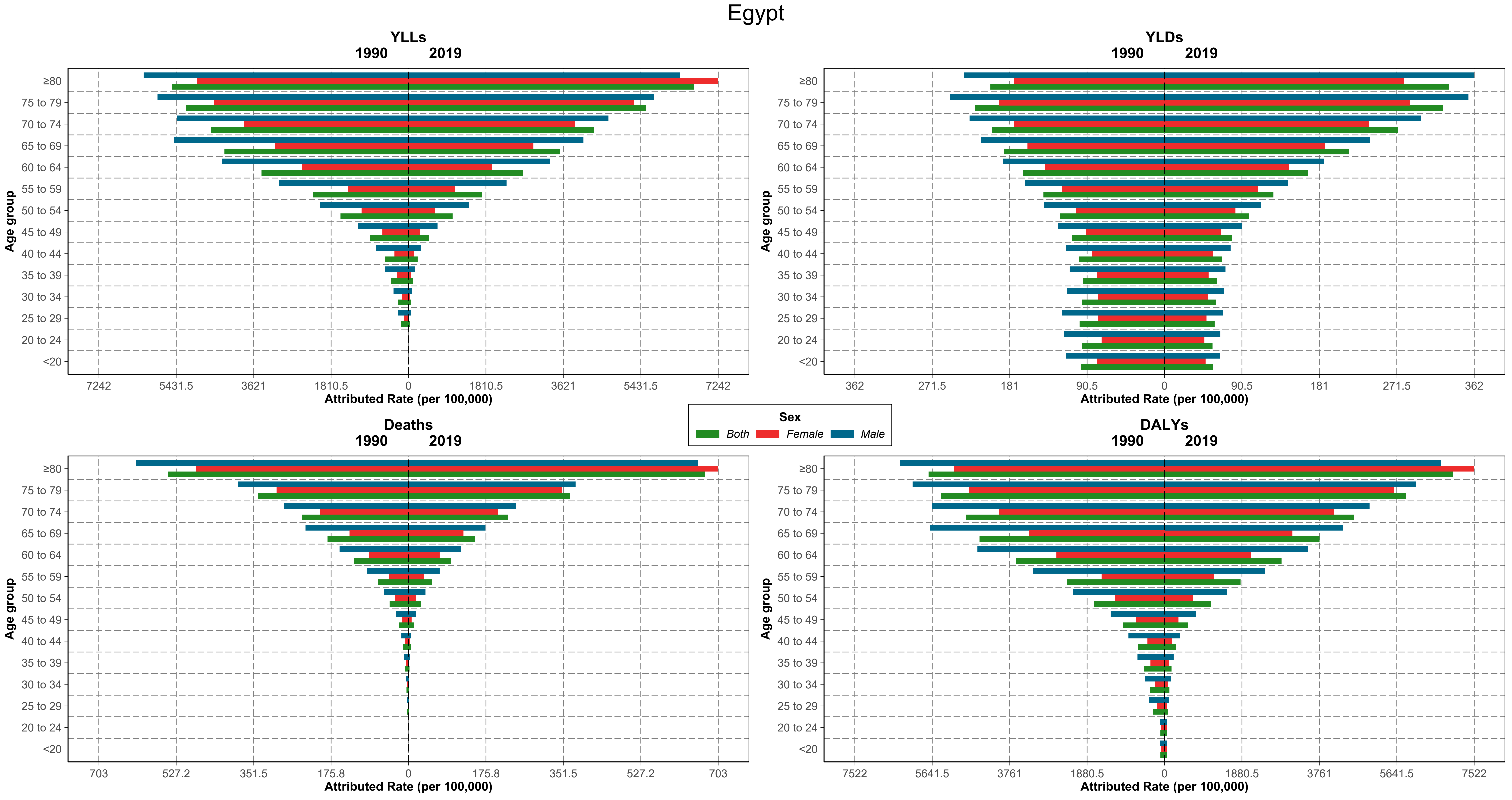

Egypt
YLLs
1990
2019
YLDs
1990
2019
Deaths
1990
2019
DALYs
1990
2019
Age group
≥80
75 to 79
70 to 74
65 to 69
60 to 64
55 to 59
50 to 54
45 to 49
40 to 44
35 to 39
30 to 34
25 to 29
20 to 24
<20
7242 5431.5 3621 1810.5 0 1810.5 3621 5431.5 7242
362 271.5 181 90.5 0 90.5 181 271.5 362
703 527.2 351.5 175.8 0 175.8 351.5 527.2 703
7522 5641.5 3761 1880.5 0 1880.5 3761 5641.5 7522
Attributed Rate (per 100,000)
Sex
Both
Female
Male

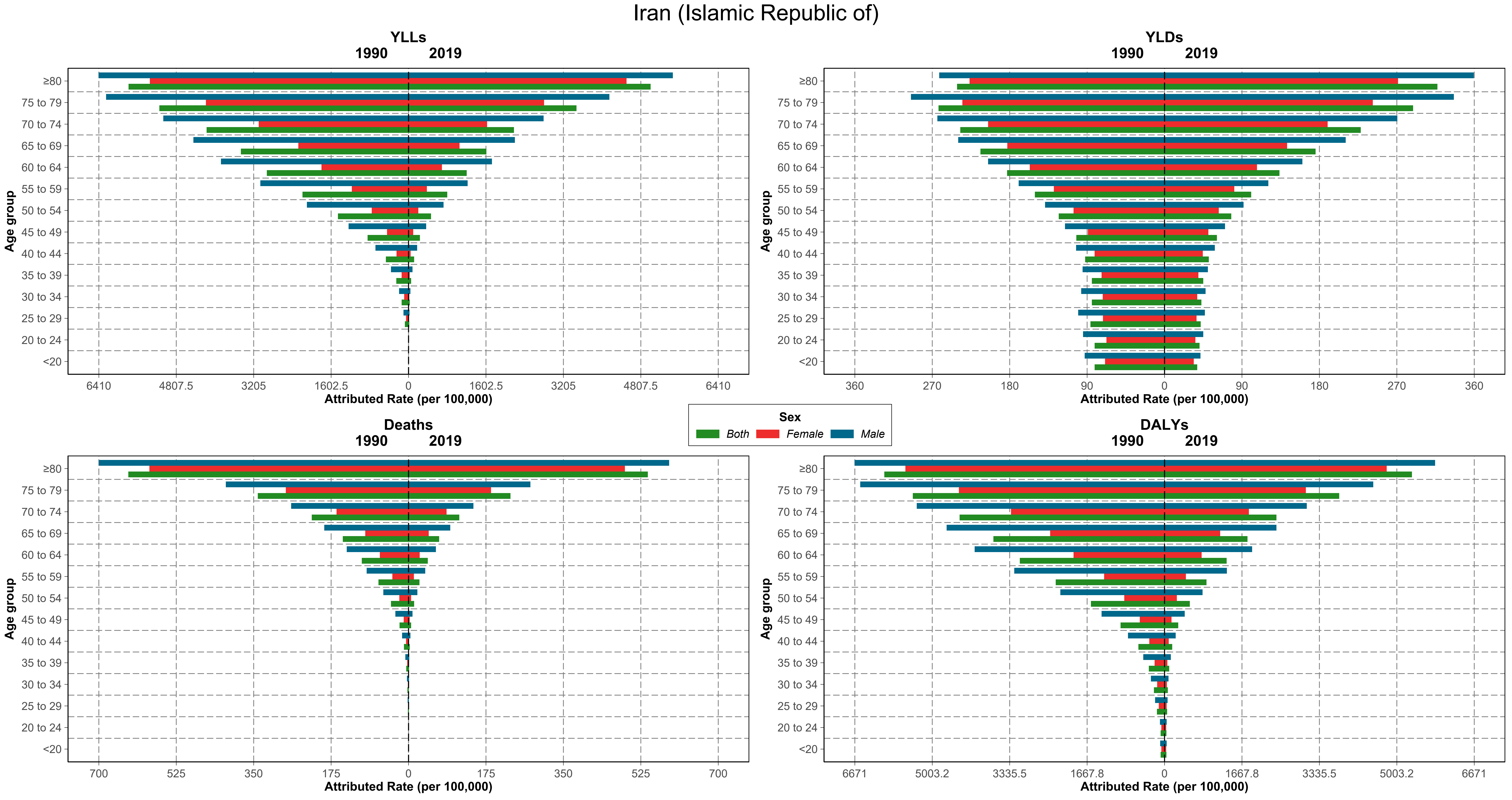
Iran (Islamic Republic of)
YLLs
1990
2019
YLDs
1990
2019
Deaths
1990
2019
DALYs
1990
2019
Age group
≥80
75 to 79
70 to 74
65 to 69
60 to 64
55 to 59
50 to 54
45 to 49
40 to 44
35 to 39
30 to 34
25 to 29
20 to 24
<20
6410
4807.5
3205
1602.5
0
1602.5
3205
4807.5
6410
360
270
180
90
0
90
180
270
360
700
525
350
175
0
175
350
525
700
6671
5003.2
3335.5
1667.8
0
1667.8
3335.5
5003.2
6671
Attributed Rate (per 100,000)
Sex
Both
Female
Male

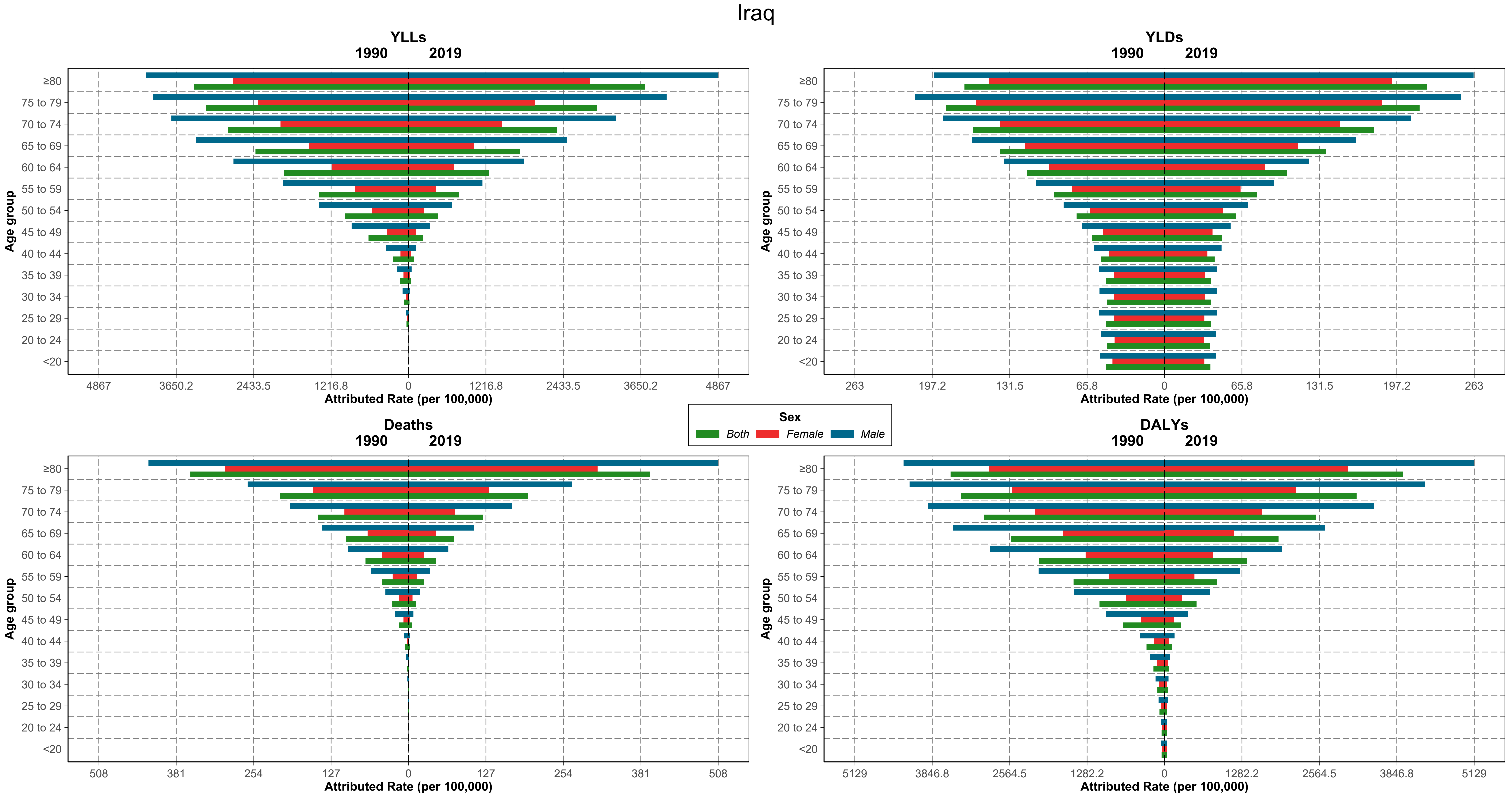
Iraq
YLLs
1990
2019
YLDs
1990
2019
Deaths
1990
2019
DALYs
1990
2019
Age group
≥80
75 to 79
70 to 74
65 to 69
60 to 64
55 to 59
50 to 54
45 to 49
40 to 44
35 to 39
30 to 34
25 to 29
20 to 24
<20
4867 3650.2 2433.5 1216.8 0 1216.8 2433.5 3650.2 4867
263 197.2 131.5 65.8 0 65.8 131.5 197.2 263
508 381 254 127 0 127 254 381 508
5129 3846.8 2564.5 1282.2 0 1282.2 2564.5 3846.8 5129
Attributed Rate (per 100,000)
Sex
Both
Female
Male

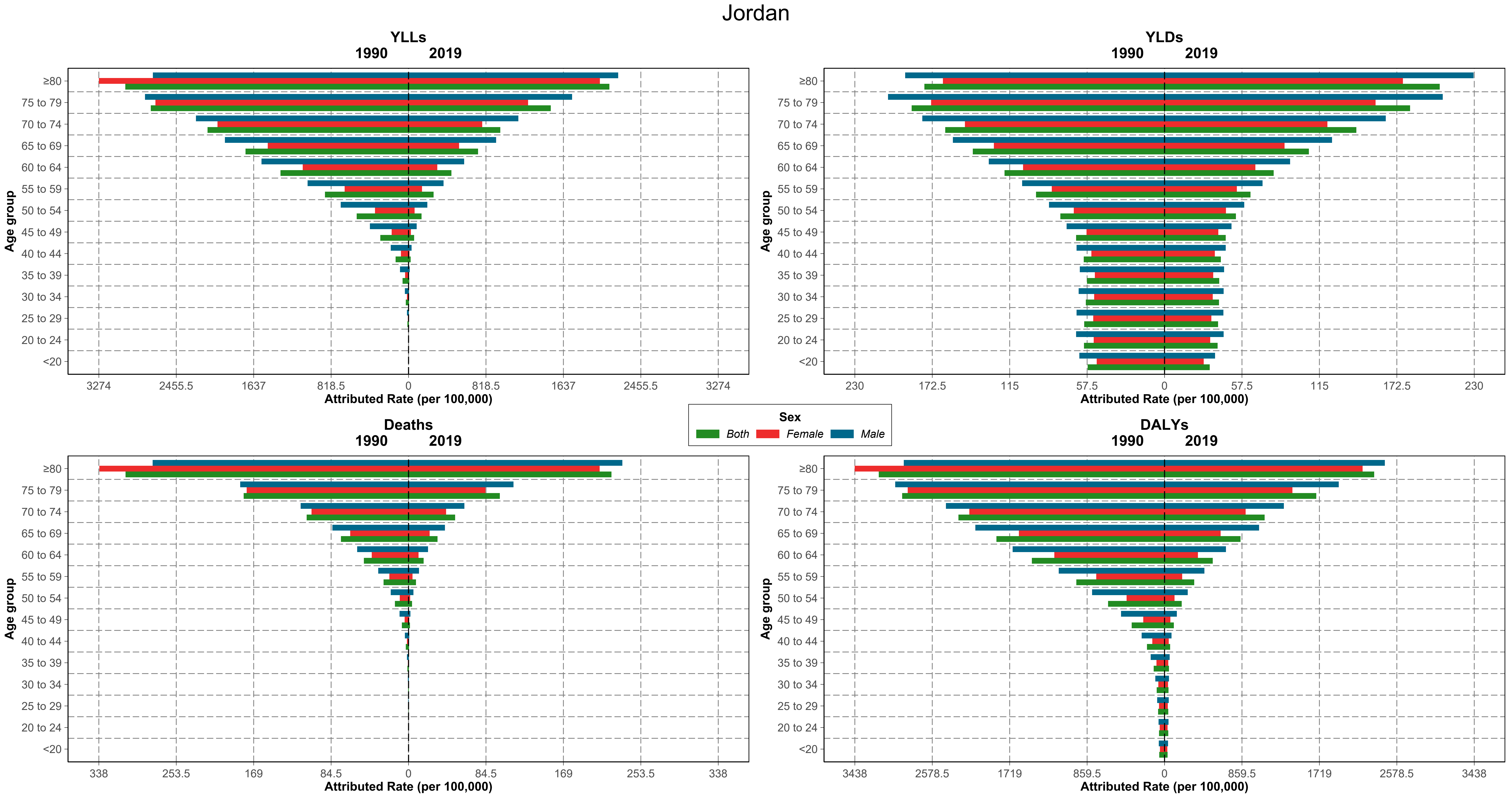

Jordan
YLLs
1990
2019
≥80
75 to 79
70 to 74
65 to 69
60 to 64
55 to 59
50 to 54
45 to 49
40 to 44
35 to 39
30 to 34
25 to 29
20 to 24
<20
Age group
3274
2455.5
1637
818.5
0
818.5
1637
2455.5
3274
Attributed Rate (per 100,000)
YLDs
1990
2019
230
172.5
115
57.5
0
57.5
115
172.5
230
Attributed Rate (per 100,000)
Sex
Both
Female
Male
Deaths
1990
2019
338
253.5
169
84.5
0
84.5
169
253.5
338
Attributed Rate (per 100,000)
DALYs
1990
2019
3438
2578.5
1719
859.5
0
859.5
1719
2578.5
3438
Attributed Rate (per 100,000)

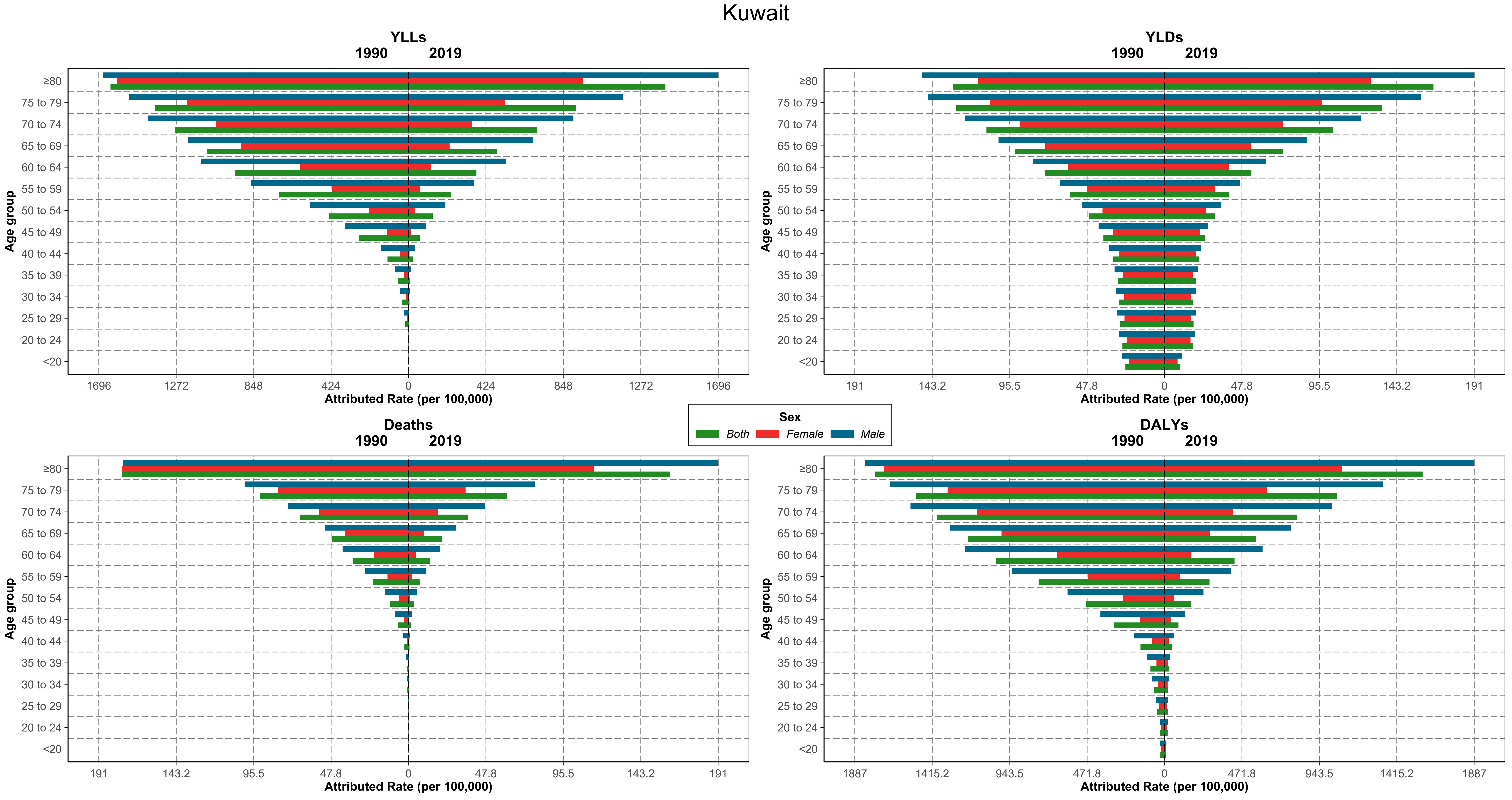
Kuwait
YLLs
1990
2019
YLDs
Deaths
DALYs
≥80
75 to 79
70 to 74
65 to 69
60 to 64
55 to 59
50 to 54
45 to 49
40 to 44
35 to 39
30 to 34
25 to 29
20 to 24
<20
Age group
1696
1272
848
424
0
191
143.2
95.5
47.8
1887
1415.2
943.5
471.8
Attributed Rate (per 100,000)
Sex
Both
Female
Male

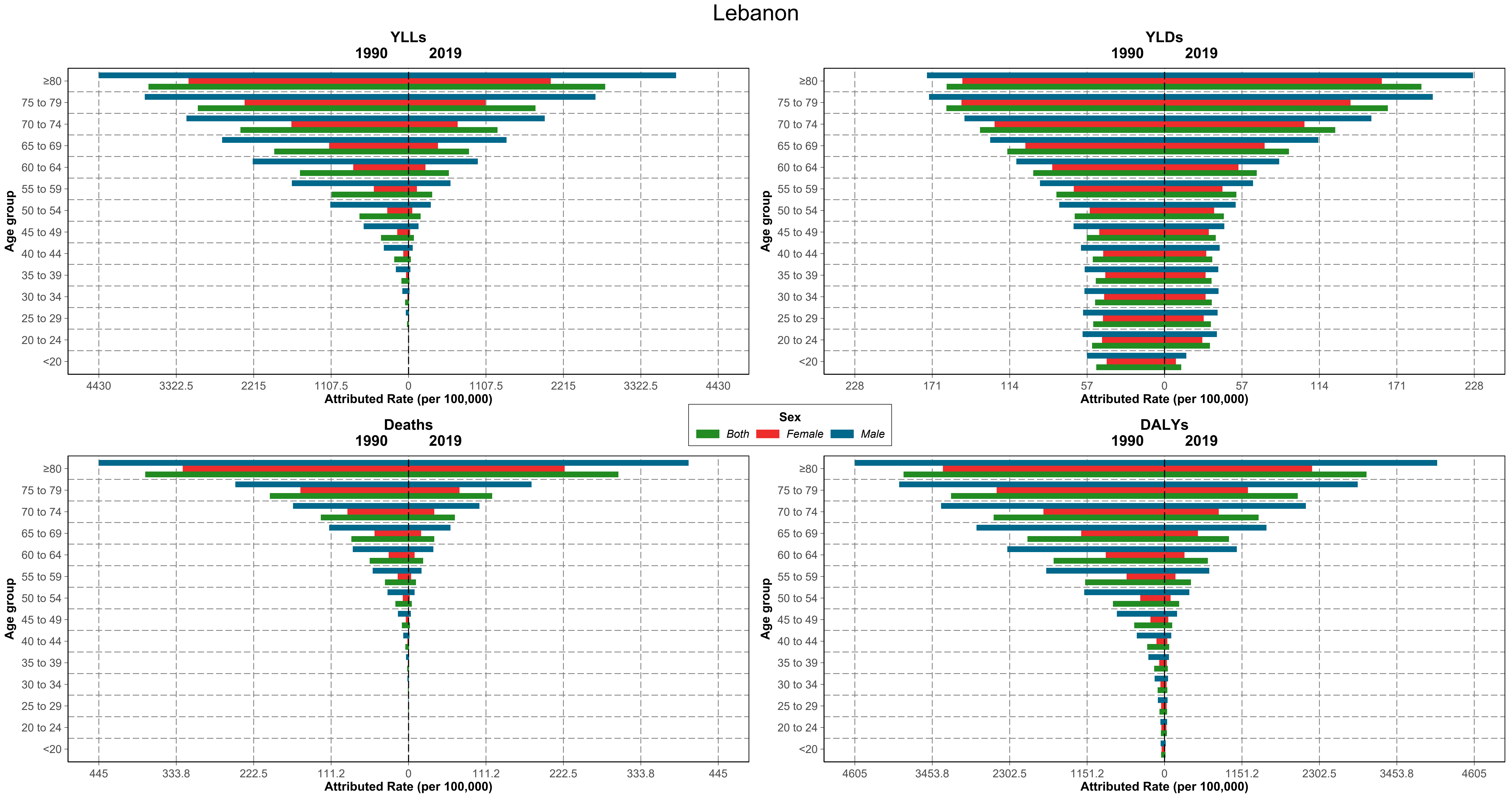
Lebanon
YLLs
1990
2019
≥80
75 to 79
70 to 74
65 to 69
60 to 64
55 to 59
50 to 54
45 to 49
40 to 44
35 to 39
30 to 34
25 to 29
20 to 24
<20
Age group
4430
3322.5
2215
1107.5
0
1107.5
2215
3322.5
4430
Attributed Rate (per 100,000)
YLDs
1990
2019
228
171
114
57
0
57
114
171
228
Attributed Rate (per 100,000)
Sex
Both
Female
Male
Deaths
1990
2019
445
333.8
222.5
111.2
0
111.2
222.5
333.8
445
Attributed Rate (per 100,000)
DALYs
1990
2019
4605
3453.8
2302.5
1151.2
0
1151.2
2302.5
3453.8
4605
Attributed Rate (per 100,000)

Libya

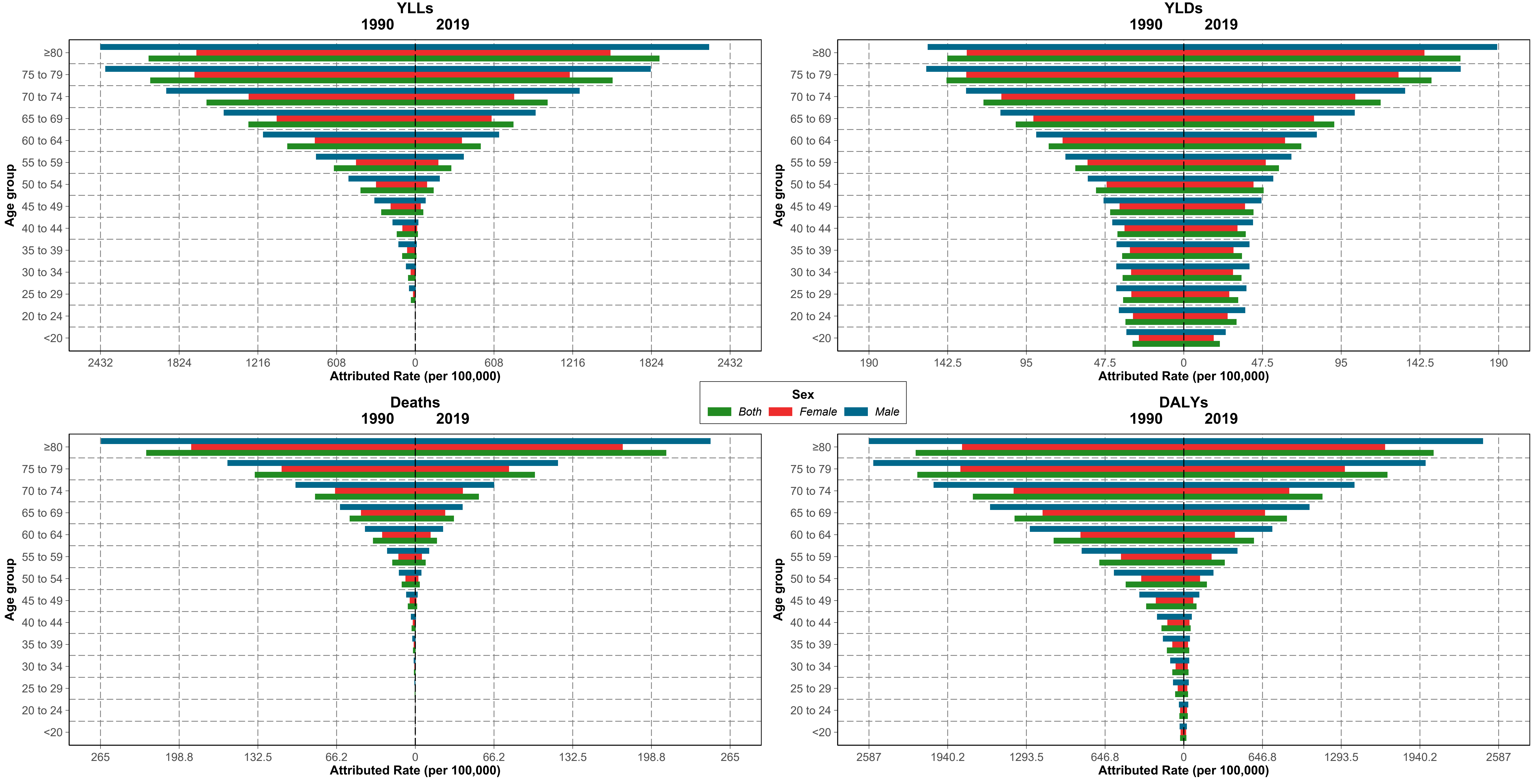
YLLs
1990
2019
YLDs
1990
2019
Deaths
1990
2019
DALYs
1990
2019
Age group
≥80
75 to 79
70 to 74
65 to 69
60 to 64
55 to 59
50 to 54
45 to 49
40 to 44
35 to 39
30 to 34
25 to 29
20 to 24
<20
2432
1824
1216
608
0
608
1216
1824
2432
190
142.5
95
47.5
0
47.5
95
142.5
190
265
198.8
132.5
66.2
0
66.2
132.5
198.8
265
2587
1940.2
1293.5
646.8
0
646.8
1293.5
1940.2
2587
Attributed Rate (per 100,000)
Sex
Both
Female
Male

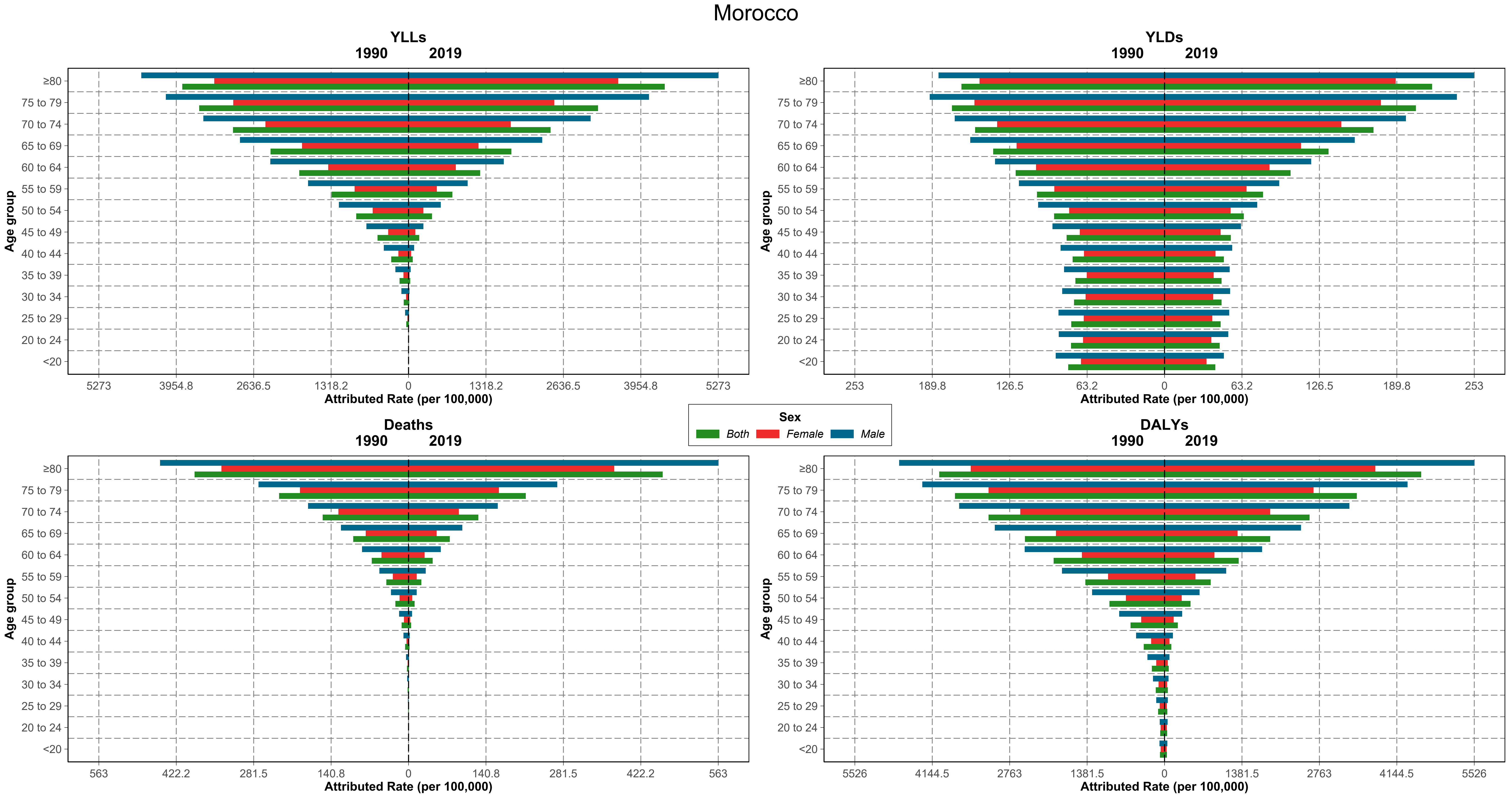
Morocco
YLLs
1990
2019
YLDs
1990
2019
Deaths
1990
2019
DALYs
1990
2019
Age group
≥80
75 to 79
70 to 74
65 to 69
60 to 64
55 to 59
50 to 54
45 to 49
40 to 44
35 to 39
30 to 34
25 to 29
20 to 24
<20
5273 3954.8 2636.5 1318.2 0 1318.2 2636.5 3954.8 5273
253 189.8 126.5 63.2 0 63.2 126.5 189.8 253
563 422.2 281.5 140.8 0 140.8 281.5 422.2 563
5526 4144.5 2763 1381.5 0 1381.5 2763 4144.5 5526
Attributed Rate (per 100,000)
Sex
Both
Female
Male

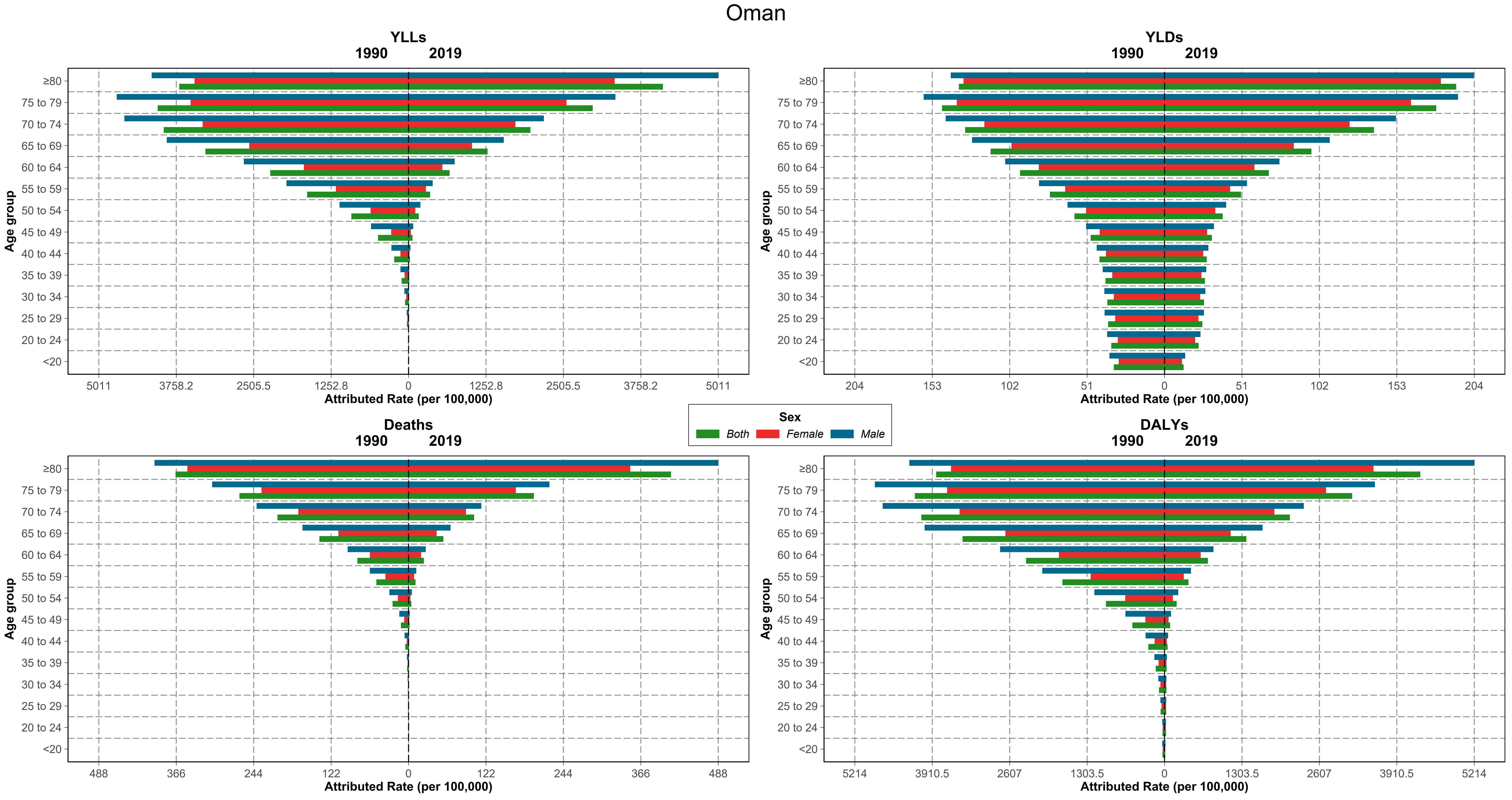

Oman
YLLs
1990
2019
YLDs
1990
2019
Deaths
1990
2019
DALYs
1990
2019
Age group
≥80
75 to 79
70 to 74
65 to 69
60 to 64
55 to 59
50 to 54
45 to 49
40 to 44
35 to 39
30 to 34
25 to 29
20 to 24
<20
5011 3758.2 2505.5 1252.8 0 1252.8 2505.5 3758.2 5011
204 153 102 51 0 51 102 153 204
488 366 244 122 0 122 244 366 488
5214 3910.5 2607 1303.5 0 1303.5 2607 3910.5 5214
Attributed Rate (per 100,000)
Sex
Both
Female
Male

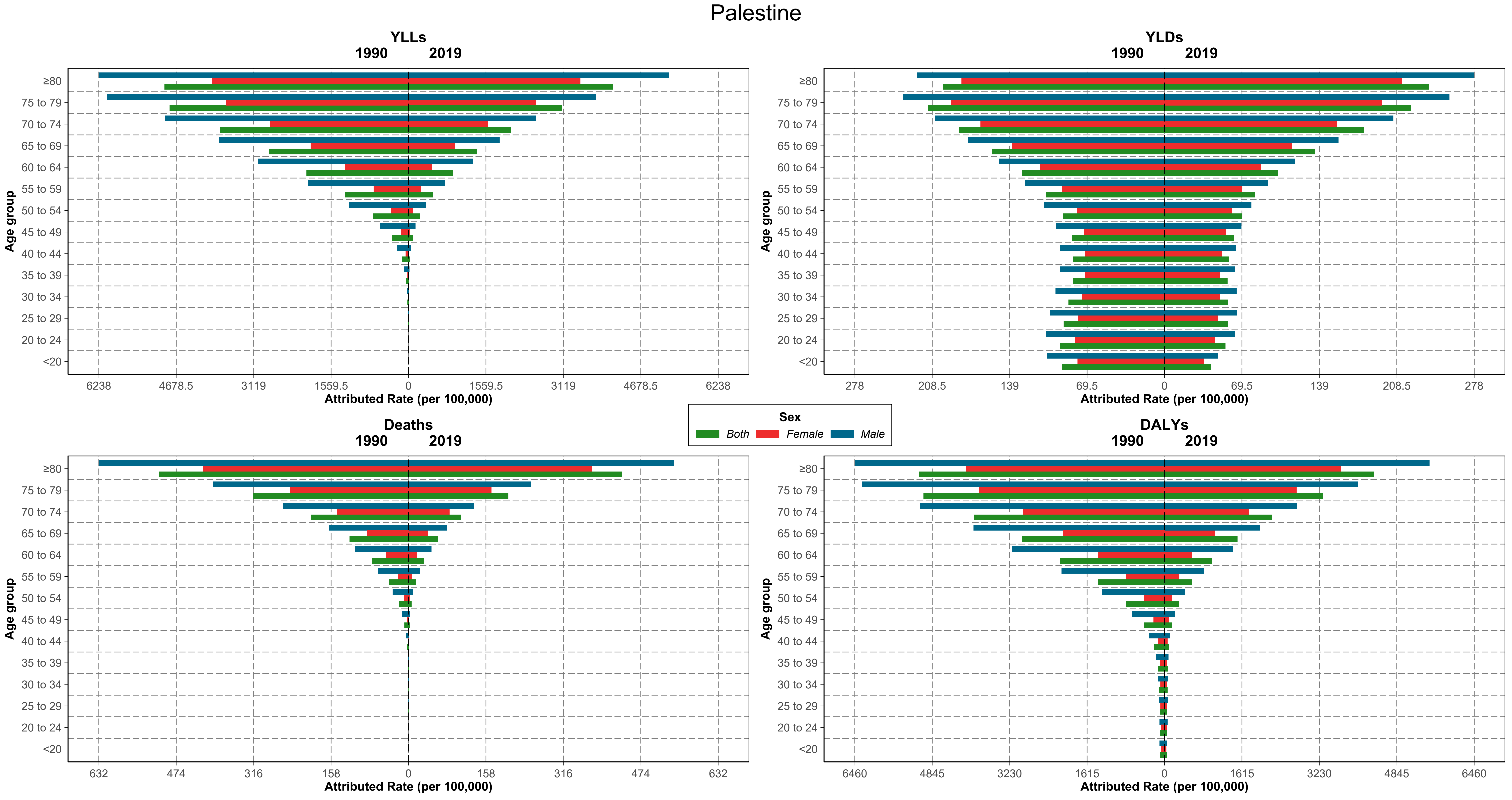

Palestine
YLLs
1990
2019
Age group
≥80
75 to 79
70 to 74
65 to 69
60 to 64
55 to 59
50 to 54
45 to 49
40 to 44
35 to 39
30 to 34
25 to 29
20 to 24
<20
6238
4678.5
3119
1559.5
0
1559.5
3119
4678.5
6238
Attributed Rate (per 100,000)
YLDs
1990
2019
278
208.5
139
69.5
0
69.5
139
208.5
278
Attributed Rate (per 100,000)
Sex
Both
Female
Male
Deaths
1990
2019
632
474
316
158
0
158
316
474
632
Attributed Rate (per 100,000)
DALYs
1990
2019
6460
4845
3230
1615
0
1615
3230
4845
6460
Attributed Rate (per 100,000)

Qatar

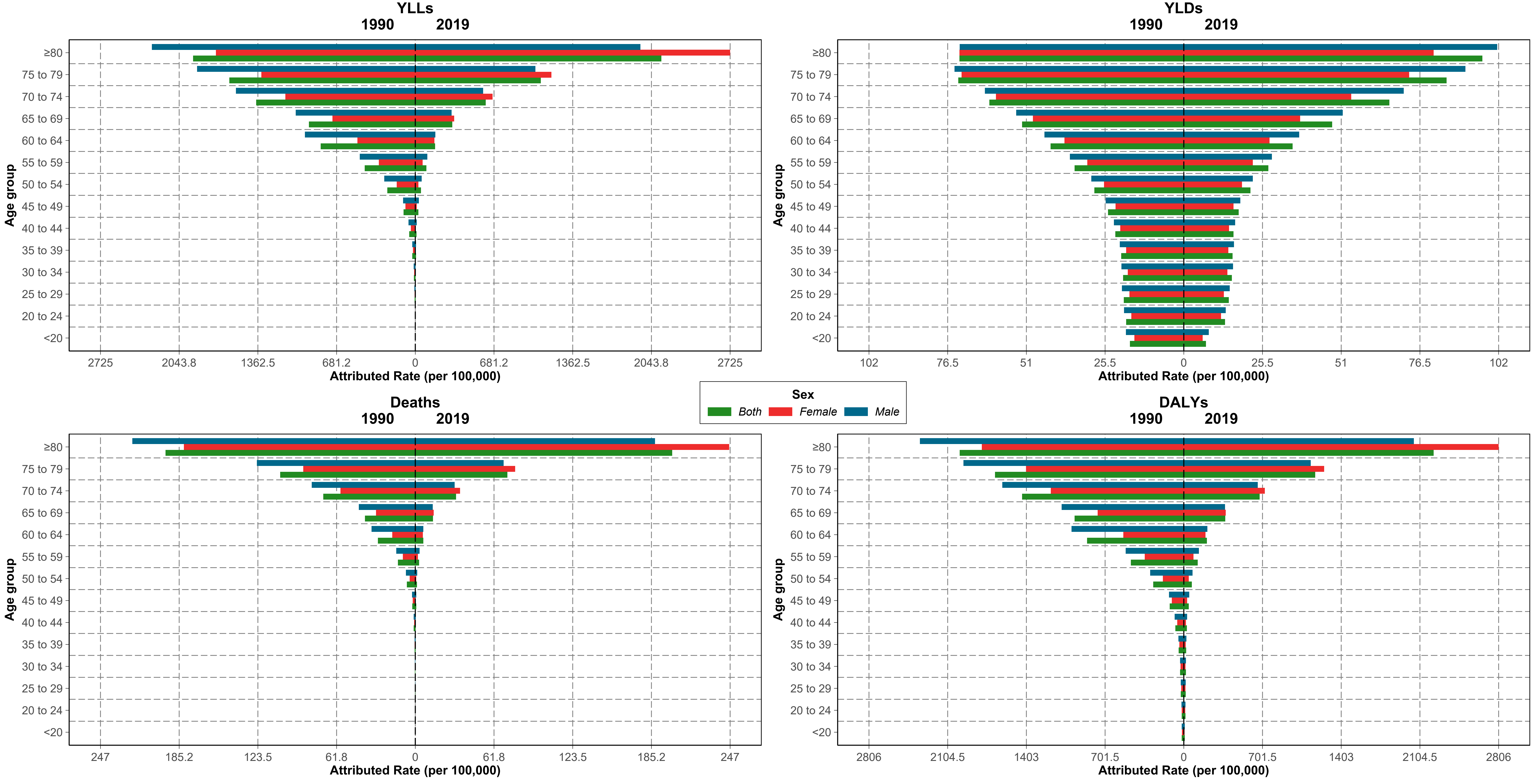
YLLs
1990
2019
≥80
75 to 79
70 to 74
65 to 69
60 to 64
55 to 59
50 to 54
45 to 49
40 to 44
35 to 39
30 to 34
25 to 29
20 to 24
<20
Age group
2725
2043.8
1362.5
681.2
0
681.2
1362.5
2043.8
2725
Attributed Rate (per 100,000)
YLDs
102
76.5
51
25.5
0
25.5
51
76.5
102
Deaths
247
185.2
123.5
61.8
0
61.8
123.5
185.2
247
DALYs
2806
2104.5
1403
701.5
0
701.5
1403
2104.5
2806
Sex
Both
Female
Male

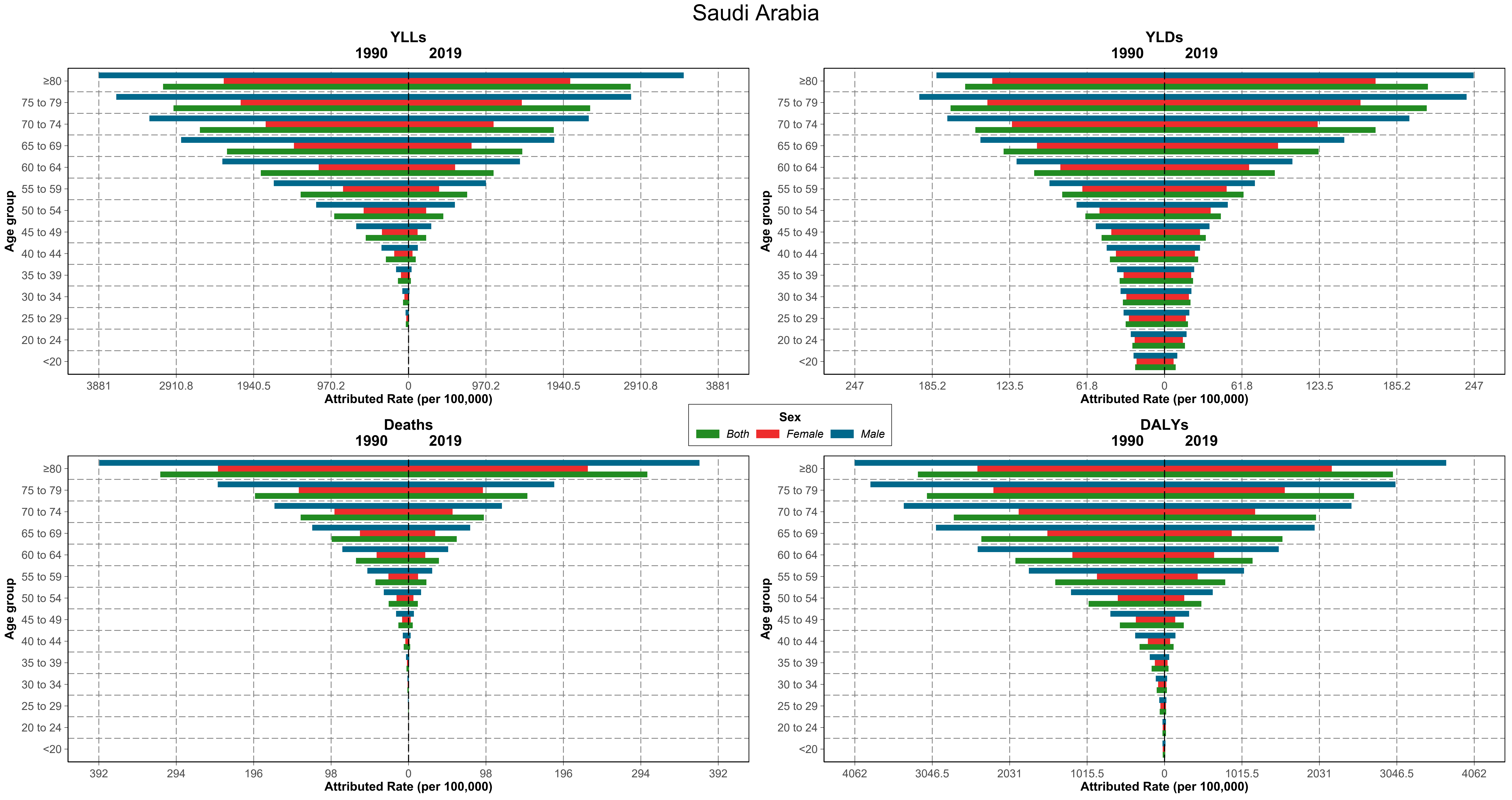

Saudi Arabia
YLLs
1990
2019
YLDs
1990
2019
Deaths
1990
2019
DALYs
1990
2019
Age group
≥80
75 to 79
70 to 74
65 to 69
60 to 64
55 to 59
50 to 54
45 to 49
40 to 44
35 to 39
30 to 34
25 to 29
20 to 24
<20
3881
2910.8
1940.5
970.2
0
247
185.2
123.5
61.8
392
294
196
98
4062
3046.5
2031
1015.5
Attributed Rate (per 100,000)
Sex
Both
Female
Male

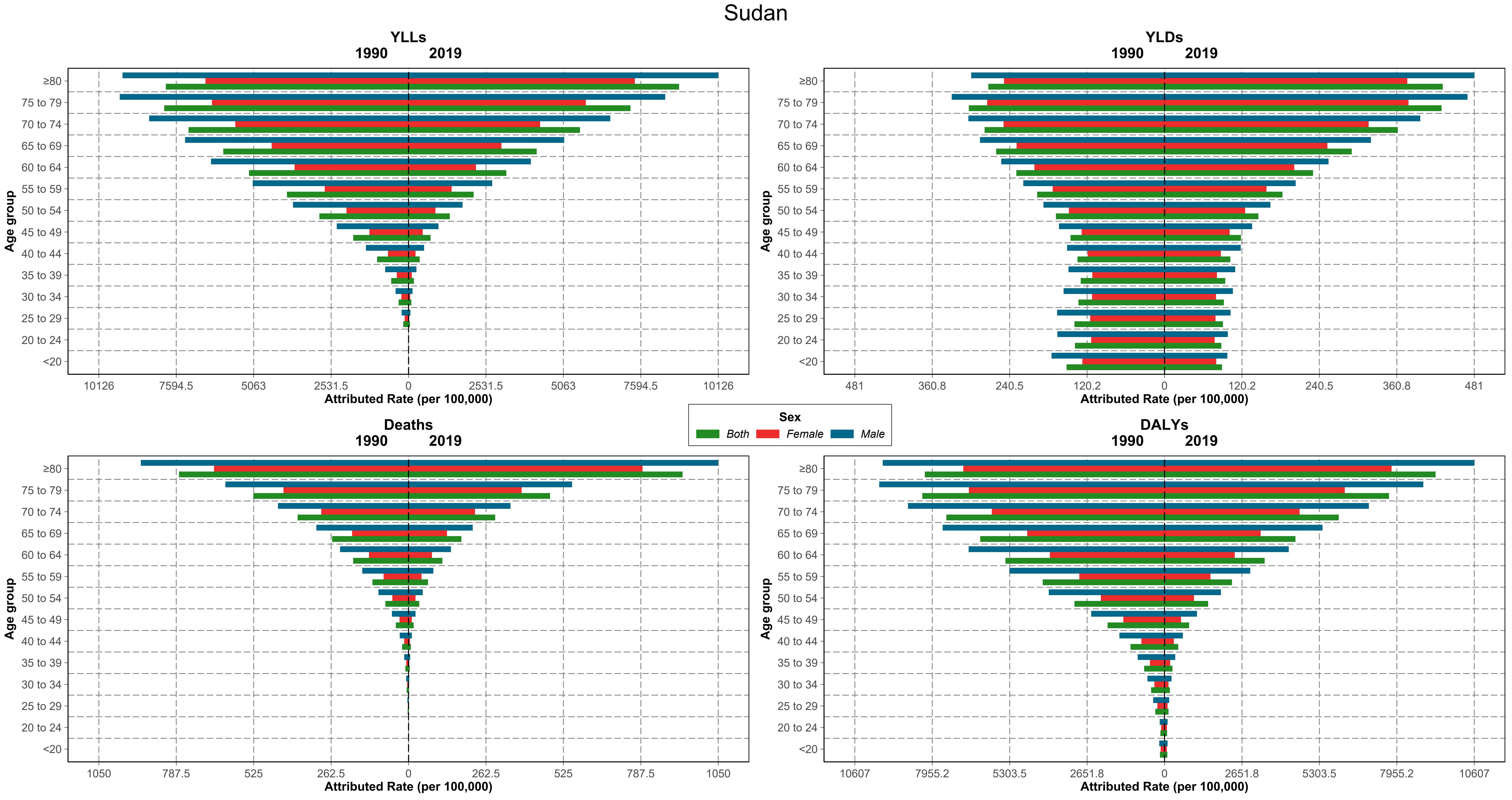

Sudan
YLLs
1990
2019
YLDs
1990
2019
Deaths
1990
2019
DALYs
1990
2019
Age group
≥80
75 to 79
70 to 74
65 to 69
60 to 64
55 to 59
50 to 54
45 to 49
40 to 44
35 to 39
30 to 34
25 to 29
20 to 24
<20
10126 7594.5 5063 2531.5 0 2531.5 5063 7594.5 10126
481 360.8 240.5 120.2 0 120.2 240.5 360.8 481
1050 787.5 525 262.5 0 262.5 525 787.5 1050
10607 7955.2 5303.5 2651.8 0 2651.8 5303.5 7955.2 10607
Attributed Rate (per 100,000)
Sex
Both
Female
Male

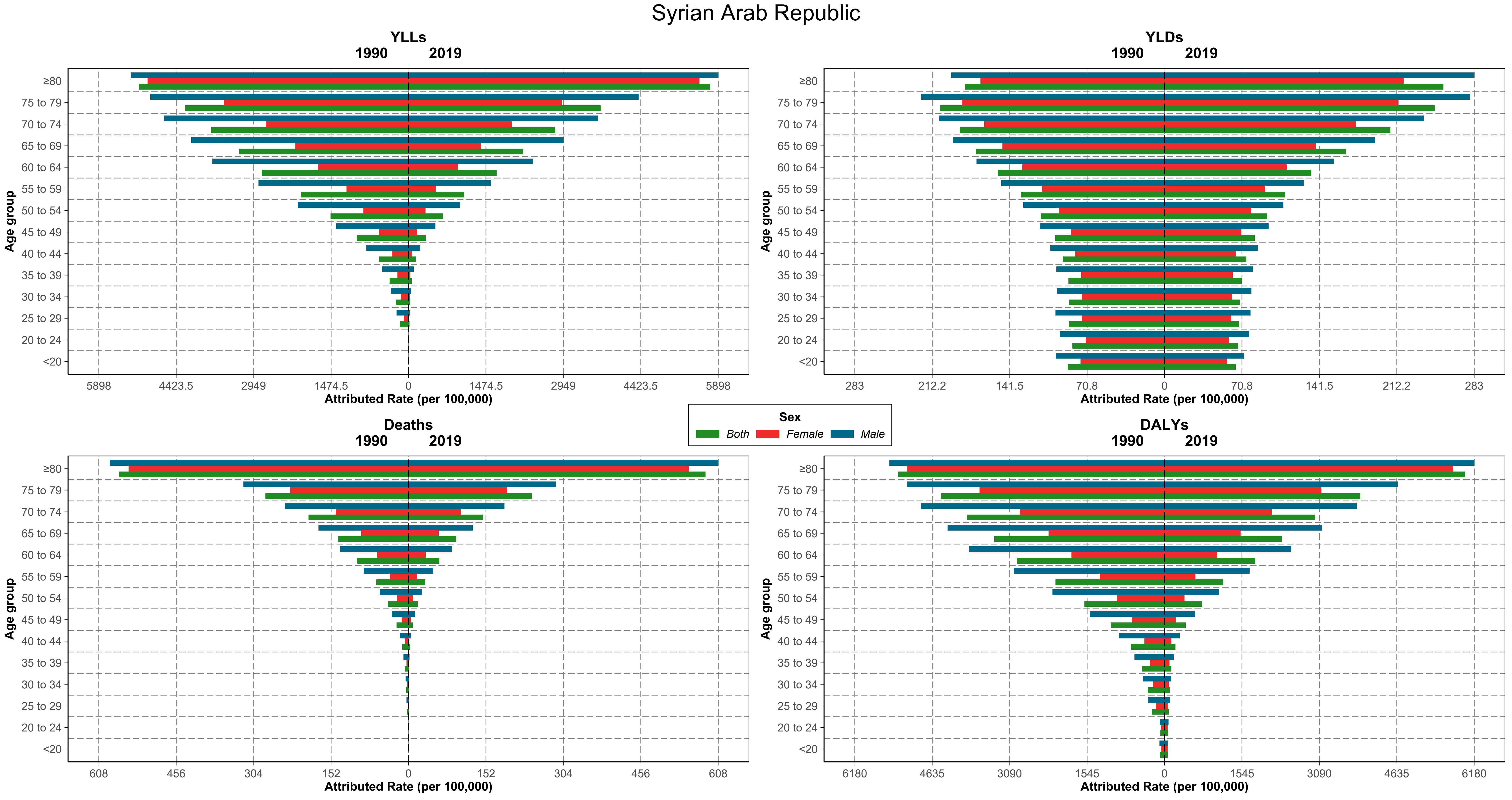

Syrian Arab Republic
YLLs
1990
2019
≥80
75 to 79
70 to 74
65 to 69
60 to 64
55 to 59
50 to 54
45 to 49
40 to 44
35 to 39
30 to 34
25 to 29
20 to 24
<20
Age group
5898
4423.5
2949
1474.5
0
1474.5
2949
4423.5
5898
Attributed Rate (per 100,000)
YLDs
1990
2019
283
212.2
141.5
70.8
0
70.8
141.5
212.2
283
Attributed Rate (per 100,000)
Sex
Both
Female
Male
Deaths
1990
2019
608
456
304
152
0
152
304
456
608
Attributed Rate (per 100,000)
DALYs
1990
2019
6180
4635
3090
1545
0
1545
3090
4635
6180
Attributed Rate (per 100,000)

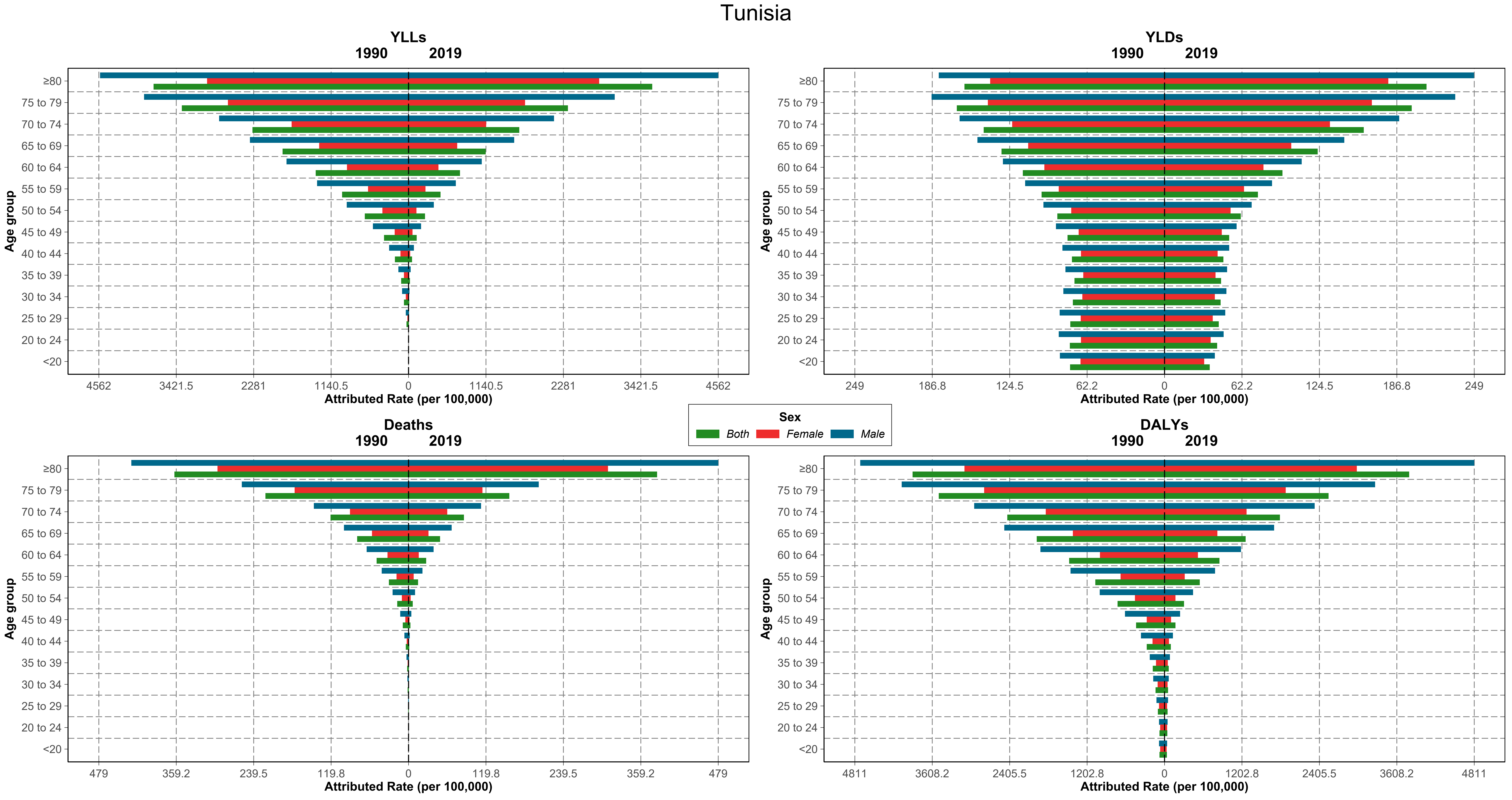

Tunisia
YLLs
1990
2019
YLDs
1990
2019
Deaths
1990
2019
DALYs
1990
2019
Age group
≥80
75 to 79
70 to 74
65 to 69
60 to 64
55 to 59
50 to 54
45 to 49
40 to 44
35 to 39
30 to 34
25 to 29
20 to 24
<20
4562 3421.5 2281 1140.5 0 1140.5 2281 3421.5 4562
249 186.8 124.5 62.2 0 62.2 124.5 186.8 249
479 359.2 239.5 119.8 0 119.8 239.5 359.2 479
4811 3608.2 2405.5 1202.8 0 1202.8 2405.5 3608.2 4811
Attributed Rate (per 100,000)
Sex
Both
Female
Male

Turkey

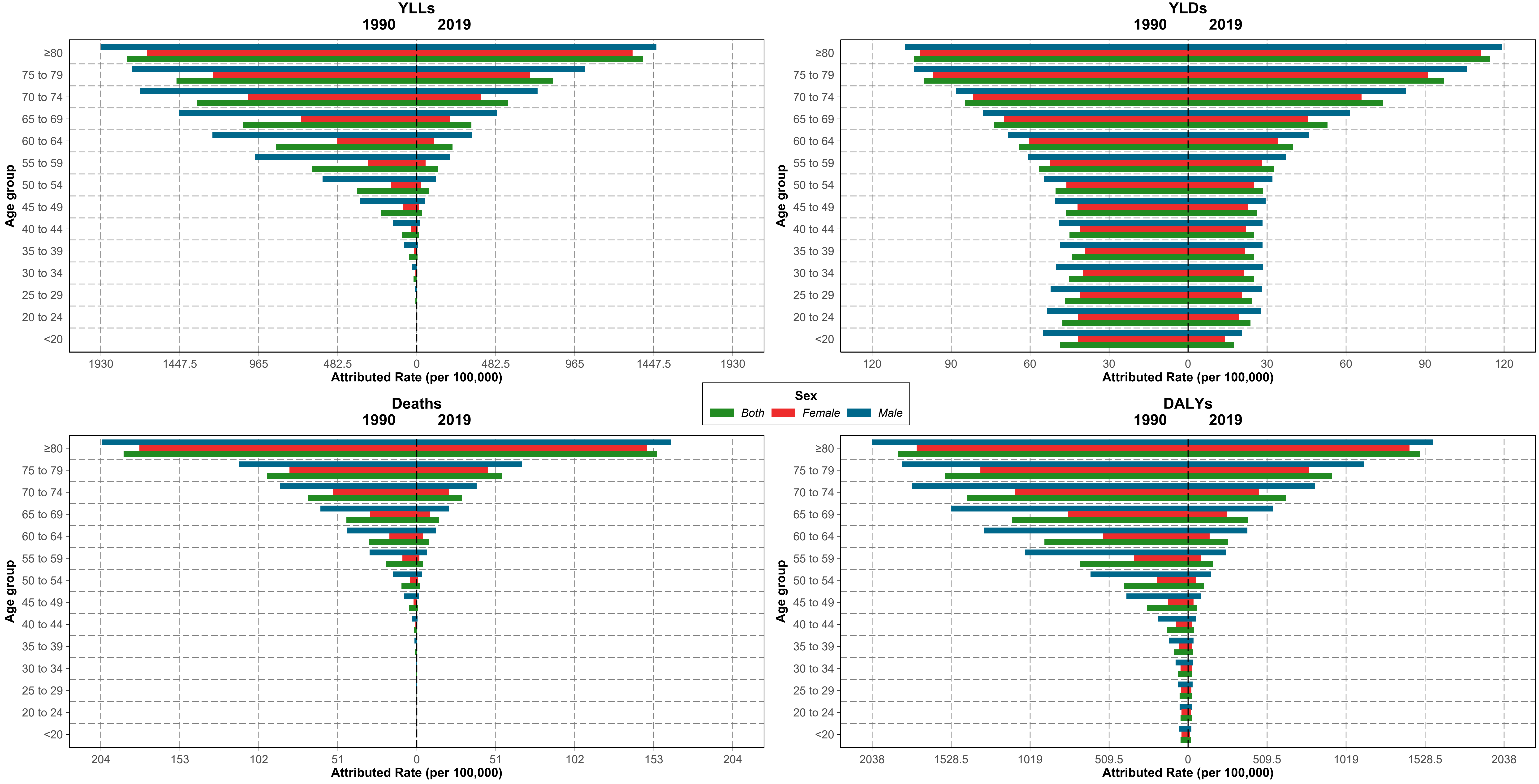
YLLs
1990
2019
YLDs
1990
2019
Deaths
1990
2019
DALYs
1990
2019
Age group
≥80
75 to 79
70 to 74
65 to 69
60 to 64
55 to 59
50 to 54
45 to 49
40 to 44
35 to 39
30 to 34
25 to 29
20 to 24
<20
1930 1447.5 965 482.5 0 482.5 965 1447.5 1930
120 90 60 30 0 30 60 90 120
204 153 102 51 0 51 102 153 204
2038 1528.5 1019 509.5 0 509.5 1019 1528.5 2038
Attributed Rate (per 100,000)
Sex
Both
Female
Male

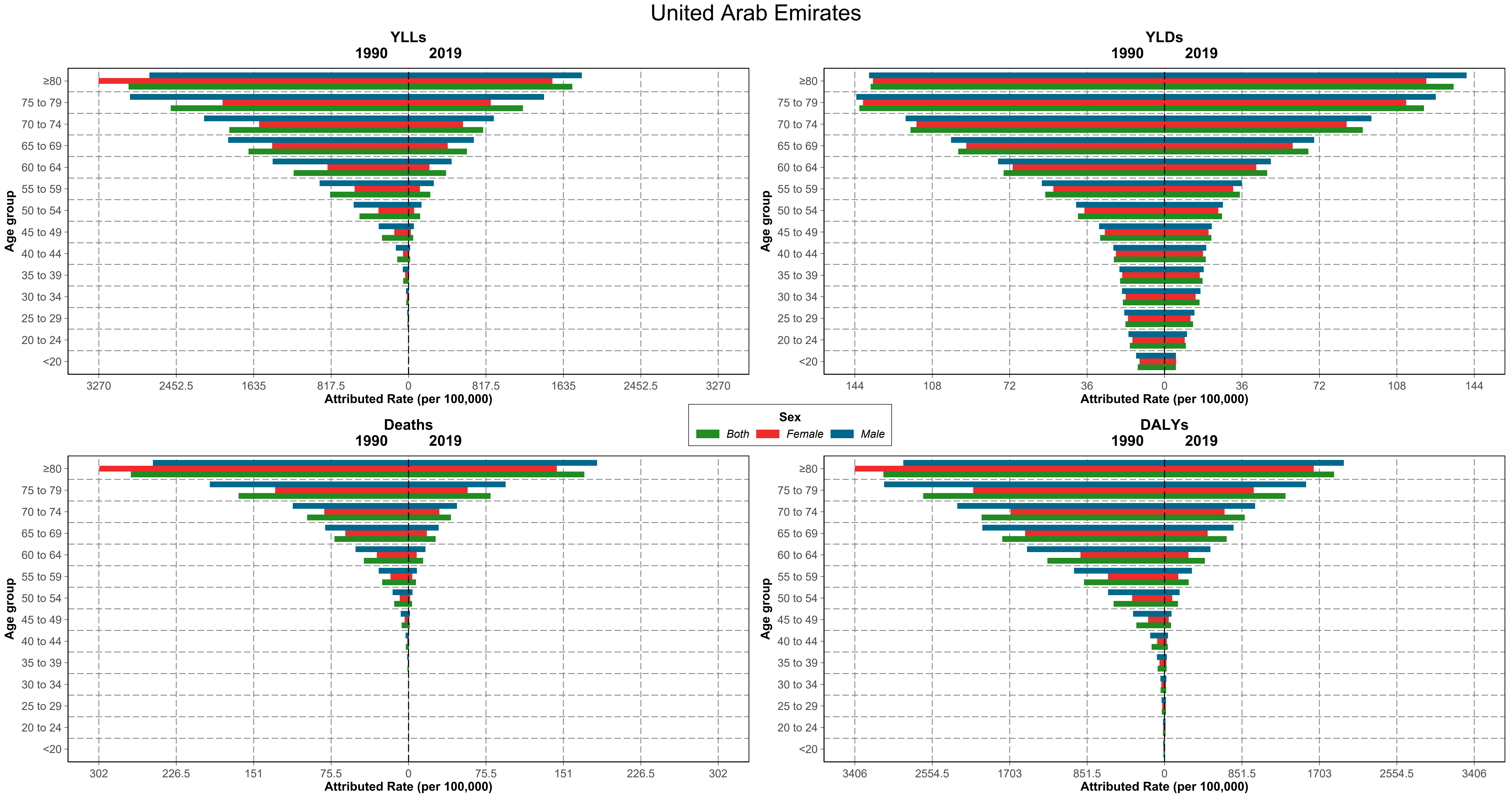

United Arab Emirates
YLLs
1990
2019
YLDs
1990
2019
Deaths
1990
2019
DALYs
1990
2019
Age group
≥80
75 to 79
70 to 74
65 to 69
60 to 64
55 to 59
50 to 54
45 to 49
40 to 44
35 to 39
30 to 34
25 to 29
20 to 24
<20
3270 2452.5 1635 817.5 0 817.5 1635 2452.5 3270
144 108 72 36 0 36 72 108 144
302 226.5 151 75.5 0 75.5 151 226.5 302
3406 2554.5 1703 851.5 0 851.5 1703 2554.5 3406
Attributed Rate (per 100,000)
Sex
Both
Female
Male

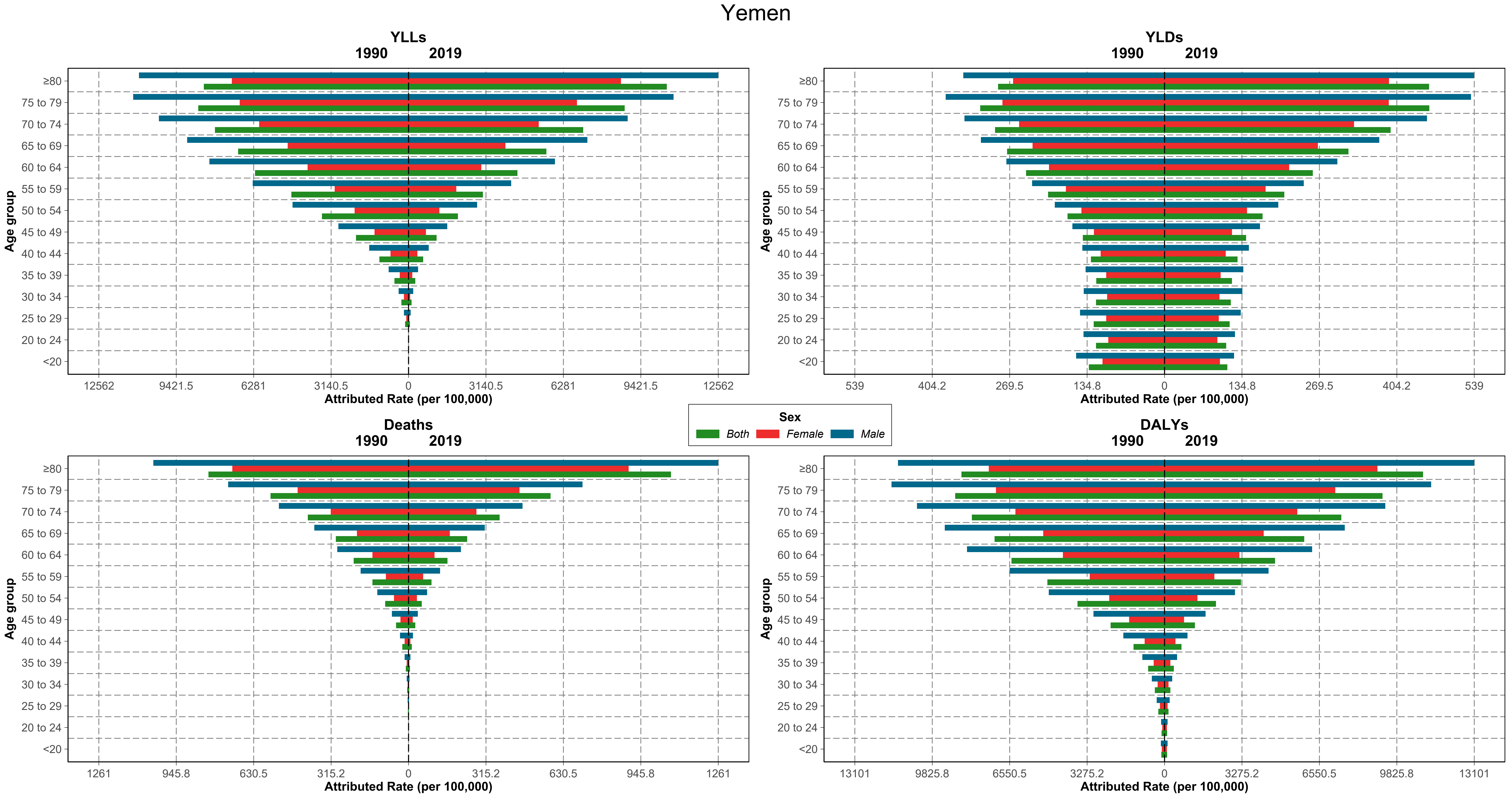

Yemen
YLLs
1990
2019
YLDs
1990
2019
Deaths
1990
2019
DALYs
1990
2019
Age group
≥80
75 to 79
70 to 74
65 to 69
60 to 64
55 to 59
50 to 54
45 to 49
40 to 44
35 to 39
30 to 34
25 to 29
20 to 24
<20
12562 9421.5 6281 3140.5 0 3140.5 6281 9421.5 12562
539 404.2 269.5 134.8 0 134.8 269.5 404.2 539
1261 945.8 630.5 315.2 0 315.2 630.5 945.8 1261
13101 9825.8 6550.5 3275.2 0 3275.2 6550.5 9825.8 13101
Attributed Rate (per 100,000)
Sex
Both
Female
Male
