# Supplementary material for: Estimating the burden of diseases attributable to lead exposure in the North Africa and Middle East region, 1990–2019: a systematic analysis for the Global Burden of Disease study 2019
Source: Environ Health. 2022 Oct 29;21:105. doi: 10.1186/s12940-022-00914-3 (PMC9617306; doi:10.1186/s12940-022-00914-3)
Supplement: Supplementary file 3 — Additional file 3: Table S1. All-age numbers and age-standardized rates of deaths, disability-adjusted-life-years (DALYs), years of life lost (YLLs), and years lived with disability (YLDs) attributable to lead exposure in 1990 and 2019 and overall percent change over 1990-2019 in North Africa and Middle East, by country. [file 12940_2022_914_MOESM3_ESM.pdf]

Table S1. All-age numbers and age-standardized rates of deaths, disability-adjusted-life-years (DALYs), years of life lost (YLLs), and years lived with disability (YLDs) attributable to lead exposure in 1990 and 2019 and overall percent change over 1990-2019 in North Africa and Middle East, by country.

Country	Measure	Age, Metric	Year						% Change (1990 to 2019)		
			1990			2019					
			Both	Female	Male	Both	Female	Male	Both	Female	Male
Afghanistan	Deaths	Attributed all ages number	5,660 (4,103 to 7,632)	2,261 (1,571 to 3,143)	3,399 (2,447 to 4,567)	8,555 (6,016 to 11,511)	3,939 (2,729 to 5,521)	4,616 (3,232 to 6,146)	51.1 (14.5 to 91.1)	74.2 (31 to 125.9)	35.8 (2.2 to 72.6)
		Attributed age-standardized rate (per 100,000)	87 (62.9 to 116.9)	72.7 (50.9 to 100.8)	99.5 (0 to 132)	82.8 (59.3 to 113.2)	74.9 (52.5 to 104.3)	91.4 (65.4 to 121.3)	-4.5 (-25 to 16.8)	3 (-19.7 to 29.6)	-8.1 (-28.8 to 13.1)
	DALYs	Attributed all ages number	165,841 (121,238 to 219,969)	68,491 (47,507 to 94,907)	97,350 (71,629 to 129,461)	283,951 (202,566 to 380,670)	127,247 (87,596 to 174,116)	156,705 (112,356 to 205,819)	71.2 (31.5 to 114.9)	85.8 (41.8 to 138.9)	61 (23.1 to 105.9)
		Attributed age-standardized rate (per 100,000)	2,176 (1579.1 to 2901.4)	1830.6 (1275.5 to 2528.9)	2502 (0 to 3346.8)	1869.9 (1349.8 to 2485.6)	1669 (1179 to 2278.3)	2089.8 (1510 to 2721.3)	-14.1 (-33.2 to 7.5)	-8.8 (-29.9 to 16.6)	-16.5 (-36.2 to 4)
	YLLs	Attributed all ages number	148,136 (105,165 to 201,091)	60,920 (41,259 to 85,600)	87,216 (61,850 to 118,308)	228,198 (156,195 to 316,597)	103,399 (68,642 to 147,873)	124,799 (84,855 to 170,149)	54 (13.6 to 102.1)	69.7 (24.1 to 125.7)	43.1 (4.7 to 88.5)
		Attributed age-standardized rate (per 100,000)	2,016 (1434.3 to 2735.7)	1692.3 (1151.5 to 2358.5)	2318.9 (0 to 3125.6)	1695.5 (1194.8 to 2290.3)	1517.6 (1051.4 to 2122.5)	1892.3 (1329.6 to 2513.6)	-15.9 (-36.3 to 7.4)	-10.3 (-32.5 to 17.3)	-18.4 (-39.2 to 3.7)
	YLDs	Attributed all ages number	17,706 (10,090 to 27,542)	7,572 (4,255 to 11,837)	10,134 (5,671 to 15,884)	55,753 (30,039 to 89,795)	23,848 (12,850 to 38,629)	31,906 (17,083 to 51,447)	214.9 (185.3 to 237.8)	215 (182 to 240.8)	214.8 (177.4 to 247.4)
		Attributed age-standardized rate (per 100,000)	161 (95.7 to 240.9)	138.3 (81.9 to 207.2)	183.2 (0 to 279.7)	174.4 (105.9 to 259.4)	151.4 (91.1 to 226.4)	197.5 (119.9 to 296.5)	8.7 (3.4 to 14.7)	9.5 (2.2 to 16.9)	7.8 (0.4 to 16)
Algeria	Deaths	Attributed all ages number	2,364 (1,226 to 3,706)	926 (400 to 1,537)	1,438 (816 to 2,202)	3,899 (1,919 to 6,212)	1,621 (685 to 2,764)	2,278 (1,216 to 3,544)	64.9 (28.1 to 109.8)	75 (35 to 121.9)	58.4 (20.4 to 103.9)
		Attributed age-standardized rate (per 100,000)	28 (14.8 to 43.1)	24.5 (11.2 to 39.9)	31.7 (0 to 47.7)	16.7 (8.7 to 26.1)	16.2 (7.4 to 26.9)	17.7 (9.8 to 27.2)	-40.4 (-51.7 to -26.8)	-34.1 (-45.7 to -19.3)	-44.2 (-56.4 to -29.9)

Country	Measure	Age, Metric	Year						% Change (1990 to 2019)		
			1990			2019					
			Both	Female	Male	Both	Female	Male	Both	Female	Male
	DALYs	Attributed all ages number	70,369 (39,832 to 103,707)	27,662 (13,712 to 42,943)	42,707 (24,937 to 61,615)	86,852 (44,691 to 136,920)	34,529 (15,969 to 57,490)	52,324 (28,012 to 80,894)	23.4 (-2.3 to 51.8)	24.8 (0 to 51.4)	22.5 (-4.9 to 53.1)
		Attributed age-standardized rate (per 100,000)	556 (303.5 to 838.6)	453.8 (217.1 to 725.2)	662.9 (0 to 973)	283.7 (152.2 to 444.9)	247.1 (118.1 to 401.6)	323.3 (179.5 to 496.6)	-48.9 (-58.8 to -37.3)	-45.5 (-55.1 to -34.6)	-51.2 (-62 to -38.6)
	YLLs	Attributed all ages number	54,711 (26,850 to 87,398)	20,768 (8,126 to 35,440)	33,943 (17,883 to 53,328)	66,980 (29,688 to 112,432)	26,002 (9,395 to 46,654)	40,978 (19,579 to 67,160)	22.4 (-9.3 to 58.6)	25.2 (-7.8 to 60.4)	20.7 (-11.9 to 59.3)
		Attributed age-standardized rate (per 100,000)	488 (247.1 to 771.1)	393.1 (166.8 to 658.1)	587.3 (0 to 906.5)	232.8 (110.5 to 376.7)	202.9 (83.1 to 349)	266.1 (134.7 to 419.8)	-52.2 (-63.5 to -39.6)	-48.4 (-59.4 to -35.9)	-54.7 (-66 to -41.2)
	YLDs	Attributed all ages number	15,658 (7,923 to 25,973)	6,894 (3,429 to 11,570)	8,764 (4,470 to 14,625)	19,872 (9,988 to 33,243)	8,527 (4,161 to 14,460)	11,345 (5,896 to 18,728)	26.9 (12.9 to 45.9)	23.7 (6.6 to 43.9)	29.5 (14.6 to 50.4)
		Attributed age-standardized rate (per 100,000)	68 (37.9 to 109.3)	60.7 (32.9 to 98)	75.6 (0 to 120.8)	50.9 (27.6 to 84.3)	44.2 (22.6 to 73.4)	57.3 (31.5 to 92.9)	-25.4 (-31.6 to -19.6)	-27.2 (-35.2 to -19.8)	-24.2 (-31.3 to -17.6)
Bahrain	Deaths	Attributed all ages number	22 (7 to 38)	7 (2 to 13)	15 (6 to 25)	32 (9 to 61)	10 (2 to 21)	21 (6 to 40)	44.7 (2.8 to 86.7)	48.9 (-0.2 to 91.6)	42.8 (-1 to 90.7)
		Attributed age-standardized rate (per 100,000)	17 (6.4 to 28.2)	12.9 (3.4 to 23.2)	20.8 (0 to 33.1)	6.9 (2.6 to 12.2)	5.8 (1.6 to 11.1)	7.9 (3.4 to 13.3)	-59.3 (-66.7 to -49)	-55.1 (-64.4 to -41.4)	-62.1 (-69.6 to -51.5)
	DALYs	Attributed all ages number	749 (308 to 1,238)	223 (78 to 385)	526 (220 to 852)	1,077 (391 to 1,953)	313 (96 to 592)	764 (290 to 1,364)	43.9 (10.9 to 77.6)	40.6 (7.8 to 71.4)	45.3 (9.2 to 83.1)
		Attributed age-standardized rate (per 100,000)	359 (140.9 to 591.8)	259.9 (76.4 to 459.4)	440.2 (0 to 698.6)	126.1 (50.8 to 217)	101.3 (31.9 to 186.9)	144.8 (65.5 to 238.2)	-64.9 (-71.4 to -56.3)	-61 (-68.5 to -48.5)	-67.1 (-73.7 to -58.3)
	YLLs	Attributed all ages number	580 (164 to 1,039)	160 (29 to 307)	420 (135 to 740)	706 (144 to 1,472)	195 (24 to 436)	511 (119 to 1,033)	21.8 (-24.5 to 63.6)	22.4 (-31.8 to 59.6)	21.6 (-25.8 to 65.8)
		Attributed age-standardized rate (per 100,000)	320 (111.6 to 540)	225 (50.2 to 414.7)	397.8 (0 to 652)	98.2 (31.6 to 181.1)	77.6 (15.9 to 155.2)	113.9 (44.3 to 198.7)	-69.3 (-76.3 to -60.9)	-65.5 (-74.6 to -54.6)	-71.4 (-78.1 to -63.1)

Country	Measure	Age, Metric	Year						% Change (1990 to 2019)		
			1990			2019					
			Both	Female	Male	Both	Female	Male	Both	Female	Male
	YLDs	Attributed all ages number	169 (82 to 295)	63 (30 to 108)	106 (51 to 184)	371 (172 to 637)	118 (52 to 204)	253 (119 to 430)	119.5 (83.5 to 162.9)	86.4 (51.9 to 124)	139.3 (97.4 to 188.7)
	YLDs	Attributed age-standardized rate (per 100,000)	39 (20.8 to 64.2)	34.9 (17.2 to 57.8)	42.4 (0 to 68.9)	27.8 (13.9 to 46.2)	23.6 (10.9 to 39.9)	30.9 (15.8 to 50.9)	-28.9 (-38.3 to -22.1)	-32.3 (-42.6 to -23.8)	-27.1 (-36.9 to -18.1)
Egypt	Deaths	Attributed all ages number	10,485 (6,940 to 14,335)	4,016 (2,396 to 5,795)	6,469 (4,356 to 8,750)	19,161 (11,627 to 28,808)	6,918 (3,917 to 10,639)	12,244 (7,587 to 18,584)	82.8 (40.5 to 130.6)	72.3 (29.3 to 120.7)	89.3 (44.4 to 144.6)
Egypt	Deaths	Attributed age-standardized rate (per 100,000)	42 (27.6 to 58.2)	33.4 (19.8 to 49.1)	50.6 (0 to 68.9)	39.3 (24.8 to 59)	37.4 (22.1 to 56.1)	42.2 (26.5 to 63.7)	-6.6 (-26.1 to 15.5)	12 (-13.1 to 42.2)	-16.6 (-34.9 to 5.4)
Egypt	DALYs	Attributed all ages number	326,119 (224,944 to 433,270)	119,514 (76,658 to 164,940)	206,605 (146,578 to 273,274)	504,964 (313,142 to 750,465)	178,953 (105,228 to 269,435)	326,011 (207,004 to 483,902)	54.8 (21 to 95.2)	49.7 (14.4 to 86.6)	57.8 (21.5 to 102.7)
Egypt	DALYs	Attributed age-standardized rate (per 100,000)	994 (675.2 to 1326.6)	750.9 (470.5 to 1054.9)	1233.6 (0 to 1644.9)	800 (500.9 to 1185.5)	678.1 (410 to 1012)	919.1 (590.6 to 1368.5)	-19.5 (-36.6 to -0.2)	-9.7 (-29.6 to 12.8)	-25.5 (-42 to -5.7)
Egypt	YLLs	Attributed all ages number	267,570 (174,867 to 364,327)	96,108 (56,345 to 138,072)	171,462 (115,778 to 231,593)	429,777 (252,569 to 661,302)	148,721 (80,203 to 237,967)	281,057 (167,736 to 431,021)	60.6 (20.4 to 108)	54.7 (11.2 to 101.3)	63.9 (20.5 to 117.8)
Egypt	YLLs	Attributed age-standardized rate (per 100,000)	881 (582.4 to 1196.8)	657.8 (393 to 948.2)	1101.6 (0 to 1491.3)	712 (427.8 to 1082.3)	603.3 (342.5 to 922.2)	819.5 (499.5 to 1252.1)	-19.2 (-38.1 to 3.1)	-8.3 (-31 to 17.3)	-25.6 (-44.3 to -3.5)
Egypt	YLDs	Attributed all ages number	58,550 (31,049 to 94,890)	23,406 (12,487 to 38,481)	35,143 (18,304 to 56,183)	75,187 (41,988 to 119,496)	30,233 (16,589 to 48,758)	44,954 (25,743 to 71,526)	28.4 (16.4 to 44.9)	29.2 (14.7 to 48.2)	27.9 (14 to 46.6)
Egypt	YLDs	Attributed age-standardized rate (per 100,000)	113 (64.3 to 174.2)	93.1 (52.5 to 146.6)	131.9 (0 to 201.7)	88 (53.3 to 133.6)	74.8 (44 to 116.3)	99.6 (60 to 150.4)	-22 (-28.9 to -13.5)	-19.6 (-28.3 to -8.7)	-24.5 (-32 to -15.6)
Iran (Islamic Rep	Deaths	Attributed all ages number	8,178 (5,888 to 10,698)	2,715 (1,789 to 3,761)	5,463 (4,053 to 7,064)	14,878 (10,366 to 20,243)	5,561 (3,598 to 7,908)	9,317 (6,717 to 12,453)	81.9 (55.8 to 107.6)	104.8 (69.3 to 138.5)	70.6 (45 to 95.4)

Country	Measure	Age, Metric	Year						% Change (1990 to 2019)		
			1990			2019					
			Both	Female	Male	Both	Female	Male	Both	Female	Male
		Attributed age-standardized rate (per 100,000)	39 (27.3 to 52.1)	29.2 (18.9 to 41.2)	47.7 (0 to 63.1)	24 (16.5 to 32.7)	19.1 (12.3 to 27.3)	28.9 (20.8 to 38.5)	-38 (-45.5 to -31.5)	-34.7 (-44.9 to -25.2)	-39.3 (-46.4 to -32.2)
	DALYs	Attributed all ages number	262,852 (194,524 to 335,512)	84,993 (58,942 to 112,815)	177,858 (134,365 to 223,336)	320,435 (227,956 to 419,539)	111,006 (74,214 to 153,768)	209,429 (152,114 to 270,695)	21.9 (6.4 to 36.3)	30.6 (10.9 to 49.3)	17.8 (3 to 32.6)
		Attributed age-standardized rate (per 100,000)	890 (650.6 to 1145.5)	620.9 (423.8 to 836.4)	1137.4 (0 to 1439.7)	454.4 (323.2 to 597.6)	329.4 (221.3 to 455.3)	580.4 (423.7 to 749.8)	-48.9 (-54.6 to -44.3)	-47 (-54 to -40.5)	-49 (-54.8 to -43.3)
	YLLs	Attributed all ages number	208,573 (150,707 to 272,274)	62,463 (40,719 to 85,884)	146,110 (108,351 to 188,042)	267,005 (184,134 to 358,695)	88,473 (55,496 to 126,385)	178,533 (126,144 to 234,941)	28 (10.7 to 43.5)	41.6 (17.5 to 63.6)	22.2 (5.1 to 38.3)
		Attributed age-standardized rate (per 100,000)	784 (561.2 to 1024.9)	529.5 (347.1 to 731.3)	1016.7 (0 to 1316)	386.6 (270.8 to 519.6)	271.7 (173.7 to 388.6)	502.6 (360 to 657.8)	-50.7 (-56.9 to -45.7)	-48.7 (-56.9 to -41.6)	-50.6 (-56.5 to -44.7)
	YLDs	Attributed all ages number	54,279 (30,269 to 85,585)	22,530 (12,236 to 35,969)	31,749 (17,813 to 49,611)	53,430 (31,730 to 82,667)	22,533 (13,015 to 35,058)	30,896 (18,411 to 47,128)	-1.6 (-12.9 to 17.5)	0 (-12.5 to 20.7)	-2.7 (-13.9 to 16.3)
		Attributed age-standardized rate (per 100,000)	106 (64.3 to 158.5)	91.4 (55 to 137.7)	120.7 (0 to 181)	67.7 (40.8 to 103.2)	57.7 (33.9 to 88.8)	77.7 (47.6 to 117.5)	-36.4 (-41.6 to -31)	-36.9 (-42.8 to -31.7)	-35.6 (-41.2 to -29.2)
Iraq	Deaths	Attributed all ages number	1,730 (940 to 2,610)	622 (282 to 1,008)	1,108 (634 to 1,650)	3,635 (1,917 to 5,508)	1,246 (564 to 2,023)	2,389 (1,327 to 3,568)	110.1 (66.5 to 157.2)	100.3 (57.4 to 149.8)	115.6 (66.4 to 175.7)
		Attributed age-standardized rate (per 100,000)	25 (13.5 to 37)	17.5 (8.1 to 28.1)	32.6 (0 to 48.4)	20.7 (11.5 to 30.8)	14 (6.8 to 22)	28.2 (16.9 to 41.2)	-16.4 (-32.2 to 0.5)	-19.9 (-35.9 to -0.1)	-13.2 (-30.6 to 8.5)
	DALYs	Attributed all ages number	49,550 (27,956 to 72,930)	17,270 (8,389 to 26,839)	32,280 (19,007 to 47,507)	95,411 (52,113 to 145,915)	31,802 (15,226 to 52,248)	63,609 (35,511 to 95,660)	92.6 (54.6 to 135.7)	84.1 (48.1 to 122.9)	97.1 (52.3 to 151.8)
		Attributed age-standardized rate (per 100,000)	572 (322.3 to 854.7)	390.4 (182.4 to 623.3)	760 (0 to 1121.4)	413.9 (227 to 617.6)	272.5 (131.6 to 437.6)	564.7 (324.6 to 826.7)	-27.7 (-41.9 to -12.2)	-30.2 (-43.7 to -14.1)	-25.7 (-42.2 to -6.1)

Country	Measure	Age, Metric	Year						% Change (1990 to 2019)		
			1990			2019					
			Both	Female	Male	Both	Female	Male	Both	Female	Male
	YLLs	Attributed all ages number	39,619 (20,336 to 61,587)	12,991 (5,283 to 21,750)	26,628 (14,377 to 41,068)	75,112 (35,065 to 121,176)	23,252 (8,756 to 40,710)	51,860 (25,867 to 82,040)	89.6 (41.2 to 142.2)	79 (31.9 to 130.7)	94.8 (43.1 to 161.1)
		Attributed age-standardized rate (per 100,000)	506 (267.5 to 778.6)	333.1 (142.9 to 546.9)	685.3 (0 to 1035.7)	356.2 (182.4 to 549.8)	223.4 (93.9 to 370)	498.3 (274.2 to 752.4)	-29.6 (-45.1 to -12.1)	-32.9 (-48.3 to -14.2)	-27.3 (-44.6 to -5.9)
	YLDs	Attributed all ages number	9,931 (5,010 to 16,567)	4,279 (2,138 to 7,231)	5,652 (2,858 to 9,357)	20,299 (10,226 to 33,338)	8,551 (4,253 to 14,426)	11,749 (5,993 to 19,262)	104.4 (89.7 to 120.5)	99.8 (80.2 to 119.7)	107.9 (89.4 to 128.1)
		Attributed age-standardized rate (per 100,000)	66 (37.2 to 104.7)	57.2 (30.8 to 92.2)	74.7 (0 to 117.1)	57.7 (31.9 to 91.3)	49.1 (25.2 to 79.1)	66.4 (37.3 to 102.8)	-12.6 (-17.9 to -7.9)	-14.3 (-20.8 to -8)	-11.2 (-18 to -4.7)
Jordan	Deaths	Attributed all ages number	195 (94 to 317)	86 (34 to 149)	109 (56 to 166)	446 (185 to 745)	169 (57 to 307)	277 (127 to 472)	129.1 (76.1 to 182.4)	96.5 (42.2 to 157.1)	154.9 (83.5 to 239.6)
		Attributed age-standardized rate (per 100,000)	19 (9.2 to 30.7)	18 (7.3 to 31.5)	19.6 (0 to 30.3)	10.2 (4.7 to 16.9)	8.9 (3.4 to 15.6)	11.4 (5.6 to 18.8)	-46 (-56.6 to -34.7)	-50.8 (-62.9 to -36.9)	-41.8 (-55.5 to -24.4)
	DALYs	Attributed all ages number	6,920 (3,859 to 10,402)	2,816 (1,366 to 4,421)	4,104 (2,404 to 5,995)	13,618 (6,682 to 21,659)	4,991 (2,272 to 8,033)	8,627 (4,358 to 14,049)	96.8 (61.5 to 135.3)	77.3 (44.9 to 117.4)	110.2 (62.6 to 164.6)
		Attributed age-standardized rate (per 100,000)	426 (225.2 to 665.7)	374.6 (170.4 to 618.5)	473.5 (0 to 701.5)	205.9 (102.2 to 331.4)	166.3 (72 to 276.5)	241.7 (125.8 to 391.8)	-51.7 (-60.4 to -42.9)	-55.6 (-64.6 to -44.8)	-49 (-60.3 to -35.8)
	YLLs	Attributed all ages number	4,562 (2,082 to 7,319)	1,818 (660 to 3,157)	2,744 (1,330 to 4,280)	8,520 (3,039 to 15,082)	2,925 (795 to 5,510)	5,595 (2,169 to 9,857)	86.8 (31 to 138.2)	60.9 (6.6 to 114.2)	103.9 (36.2 to 178)
		Attributed age-standardized rate (per 100,000)	354 (169.8 to 576.9)	309.8 (120.3 to 537.2)	393.4 (0 to 601.7)	154.8 (63 to 258.2)	122.1 (40.4 to 222.8)	184.5 (83.2 to 314.4)	-56.2 (-66.7 to -45.8)	-60.6 (-71.6 to -48.5)	-53.1 (-66.4 to -37.2)
	YLDs	Attributed all ages number	2,358 (1,167 to 3,994)	997 (472 to 1,715)	1,360 (654 to 2,290)	5,098 (2,545 to 8,859)	2,066 (999 to 3,531)	3,033 (1,506 to 5,259)	116.2 (94.1 to 142.6)	107.1 (78.8 to 138.7)	122.9 (95.8 to 153.5)

Country	Measure	Age, Metric	Year						% Change (1990 to 2019)		
			1990			2019					
			Both	Female	Male	Both	Female	Male	Both	Female	Male
		Attributed age-standardized rate (per 100,000)	73 (40.2 to 115.2)	64.8 (33.9 to 103.8)	80.1 (0 to 126.7)	51.1 (26.8 to 83.1)	44.2 (22.8 to 72.9)	57.2 (30.7 to 92.4)	-29.7 (-35.9 to -24)	-31.7 (-39 to -24.6)	-28.6 (-35.9 to -21.8)
Kuwait	Deaths	Attributed all ages number	60 (27 to 95)	17 (6 to 31)	43 (21 to 65)	132 (52 to 229)	27 (8 to 52)	106 (44 to 181)	120.5 (74.7 to 165.5)	55.4 (18.5 to 92.7)	146.8 (87.3 to 208.9)
Kuwait	Deaths	Attributed age-standardized rate (per 100,000)	12 (5.6 to 18.5)	9.3 (3.3 to 16.3)	13 (0 to 19.6)	6.7 (3 to 11)	3.7 (1.2 to 6.9)	8.7 (4.1 to 14.1)	-41.9 (-51.9 to -31.3)	-59.6 (-68.8 to -49.4)	-32.7 (-46.3 to -17.7)
Kuwait	DALYs	Attributed all ages number	2,163 (1,057 to 3,311)	566 (240 to 931)	1,597 (809 to 2,423)	3,617 (1,448 to 6,395)	804 (311 to 1,437)	2,813 (1,095 to 4,998)	67.3 (27.9 to 105.7)	42.1 (15.6 to 67.8)	76.2 (27.3 to 125.1)
Kuwait	DALYs	Attributed age-standardized rate (per 100,000)	274 (138.8 to 415.8)	200.3 (79 to 334.2)	315.3 (0 to 464.3)	136.5 (59.6 to 225.2)	73.9 (27.3 to 130.5)	179.2 (80.7 to 300.6)	-50.1 (-59.8 to -41)	-63.1 (-70.5 to -55.9)	-43.2 (-56.2 to -30.3)
Kuwait	YLLs	Attributed all ages number	1,632 (656 to 2,642)	371 (101 to 677)	1,260 (550 to 2,004)	2,638 (801 to 5,043)	431 (87 to 925)	2,207 (690 to 4,178)	61.7 (7.3 to 106.4)	16.1 (-25.6 to 49.1)	75.1 (12.8 to 131.6)
Kuwait	YLLs	Attributed age-standardized rate (per 100,000)	235 (109.8 to 367.6)	167 (54.5 to 293.3)	272.9 (0 to 410.5)	109.7 (43.2 to 189.4)	52.2 (13.9 to 100.5)	148.7 (61.3 to 257.5)	-53.3 (-64.8 to -42.9)	-68.7 (-78.1 to -60.8)	-45.5 (-60.5 to -31.1)
Kuwait	YLDs	Attributed all ages number	531 (262 to 909)	195 (89 to 340)	337 (170 to 572)	979 (449 to 1,649)	373 (163 to 645)	606 (285 to 1,003)	84.3 (54.9 to 120.4)	91.7 (59.2 to 135.3)	80 (47.7 to 118.7)
Kuwait	YLDs	Attributed age-standardized rate (per 100,000)	39 (20.9 to 61.3)	33.2 (16.4 to 52.9)	42.5 (0 to 66.9)	26.8 (13.3 to 43.2)	21.7 (9.6 to 36.7)	30.5 (15.4 to 48.5)	-30.7 (-39.8 to -22.6)	-34.7 (-44.4 to -26.4)	-28.2 (-39.4 to -18.9)
Lebanon	Deaths	Attributed all ages number	429 (228 to 660)	145 (65 to 239)	284 (160 to 424)	646 (313 to 1,013)	226 (87 to 393)	420 (220 to 640)	50.5 (9.7 to 79.7)	55.7 (5.8 to 90.7)	47.8 (9 to 83.2)
Lebanon	Deaths	Attributed age-standardized rate (per 100,000)	22 (11.9 to 34.2)	15.6 (7.2 to 25.5)	29.5 (0 to 43.5)	12.9 (6.2 to 20.1)	8.1 (3.1 to 14.1)	18.8 (9.8 to 28.7)	-42.4 (-57.6 to -32.1)	-47.8 (-65.2 to -36.6)	-36.3 (-52.1 to -23.3)
Lebanon	DALYs	Attributed all ages number	11,379 (6,502 to 16,648)	3,577 (1,812 to 5,632)	7,802 (4,657 to 11,331)	12,100 (5,937 to 19,401)	3,957 (1,643 to 6,824)	8,143 (4,205 to 12,794)	6.3 (-18.9 to 28.6)	10.6 (-19.5 to 36.4)	4.4 (-20.3 to 30.5)

Country	Measure	Age, Metric	Year						% Change (1990 to 2019)		
			1990			2019					
			Both	Female	Male	Both	Female	Male	Both	Female	Male
		Attributed age-standardized rate (per 100,000)	487 (271.5 to 732)	313.2 (155.2 to 496.1)	665.1 (0 to 981.4)	234 (114.6 to 374.3)	139.8 (58.4 to 240.2)	348.5 (179.3 to 543.2)	-52 (-62.9 to -43)	-55.4 (-67.7 to -45.7)	-47.6 (-59.7 to -35.7)
	YLLs	Attributed all ages number	9,452 (4,852 to 14,649)	2,755 (1,120 to 4,628)	6,697 (3,659 to 10,159)	10,090 (4,424 to 16,670)	3,086 (1,002 to 5,684)	7,004 (3,370 to 11,297)	6.8 (-22.8 to 32.9)	12 (-26.3 to 40.9)	4.6 (-23.4 to 33.7)
		Attributed age-standardized rate (per 100,000)	424 (219.4 to 657.3)	258.7 (110.1 to 432.9)	593.5 (0 to 892.1)	195.8 (85.8 to 322)	108.5 (35.1 to 199.5)	302.7 (145.9 to 485.4)	-53.8 (-66.6 to -43.9)	-58.1 (-73.1 to -47.2)	-49 (-62.5 to -36)
	YLDs	Attributed all ages number	1,927 (994 to 3,147)	822 (426 to 1,366)	1,105 (580 to 1,810)	2,010 (1,071 to 3,221)	871 (430 to 1,417)	1,138 (633 to 1,813)	4.3 (-10.4 to 25.9)	6 (-12.1 to 30)	3 (-11.7 to 23.1)
		Attributed age-standardized rate (per 100,000)	63 (34.8 to 101)	54.6 (29.3 to 88.2)	71.6 (0 to 114)	38.2 (20.4 to 61.5)	31.3 (15.4 to 51.1)	45.9 (25.5 to 72.6)	-39.4 (-46.3 to -31.7)	-42.6 (-50.9 to -34.4)	-36 (-43.6 to -26.8)
Libya	Deaths	Attributed all ages number	231 (114 to 366)	88 (38 to 146)	143 (77 to 221)	398 (161 to 697)	152 (48 to 283)	245 (110 to 412)	72.1 (18 to 129.4)	73.2 (12.5 to 125.4)	71.4 (19.4 to 133.3)
		Attributed age-standardized rate (per 100,000)	14 (7 to 21.8)	11.1 (5 to 18.4)	16.4 (0 to 25.4)	9.6 (4.1 to 16.2)	7.4 (2.5 to 13.3)	11.7 (5.6 to 19.1)	-31.1 (-51.1 to -10.3)	-33.8 (-55.8 to -14.4)	-28.5 (-48.9 to -5.5)
	DALYs	Attributed all ages number	6,747 (3,635 to 10,259)	2,499 (1,189 to 3,956)	4,248 (2,411 to 6,354)	9,533 (4,094 to 16,469)	3,592 (1,276 to 6,509)	5,941 (2,784 to 9,986)	41.3 (1.8 to 84.7)	43.8 (-1.1 to 90.8)	39.9 (0 to 85.6)
		Attributed age-standardized rate (per 100,000)	319 (169.7 to 490.5)	252.8 (117.2 to 408.6)	375.9 (0 to 567.6)	193.6 (85.8 to 325.7)	149 (53 to 265.1)	236.8 (114.5 to 386.2)	-39.2 (-55.9 to -21)	-41.1 (-58.8 to -24.8)	-37 (-54.6 to -17.5)
	YLLs	Attributed all ages number	5,124 (2,337 to 8,312)	1,821 (689 to 3,126)	3,303 (1,635 to 5,287)	6,944 (2,238 to 13,568)	2,521 (571 to 5,199)	4,424 (1,614 to 8,184)	35.5 (-18.8 to 90.1)	38.4 (-27.1 to 95.3)	33.9 (-18 to 91.1)
		Attributed age-standardized rate (per 100,000)	272 (132.5 to 434)	211.5 (85.1 to 354.8)	324.5 (0 to 505.6)	152.6 (55.1 to 279.2)	114 (29.6 to 221.1)	190.1 (78.2 to 330.6)	-43.9 (-64.5 to -23.2)	-46.1 (-69.3 to -26.6)	-41.4 (-62.6 to -18.2)
	YLDs	Attributed all ages number	1,623 (800 to 2,745)	677 (326 to 1,148)	945 (470 to 1,596)	2,589 (1,248 to 4,342)	1,071 (481 to 1,844)	1,518 (747 to 2,561)	59.6 (39.5 to 81.6)	58.2 (34 to 85.3)	60.5 (38.7 to 85.3)

Country	Measure	Age, Metric	Year						% Change (1990 to 2019)		
			1990			2019					
			Both	Female	Male	Both	Female	Male	Both	Female	Male
		Attributed age-standardized rate (per 100,000)	47 (26 to 74.2)	41.3 (21.4 to 65.6)	51.3 (0 to 80.8)	41 (20.6 to 67.2)	35 (16.2 to 58.9)	46.7 (24.2 to 77)	-12 (-23.3 to -2.4)	-15 (-28.6 to -3.1)	-9 (-22 to 3)
Morocco	Deaths	Attributed all ages number	2,992 (1,702 to 4,406)	1,187 (593 to 1,854)	1,805 (1,060 to 2,605)	5,512 (2,885 to 8,397)	2,097 (970 to 3,391)	3,416 (1,888 to 5,076)	84.2 (44.1 to 117.1)	76.7 (40 to 112)	89.2 (42.3 to 130.2)
		Attributed age-standardized rate (per 100,000)	26 (14.7 to 38)	20.5 (10.5 to 31.8)	31.6 (0 to 45.2)	22.3 (12 to 33.9)	17.1 (8.3 to 27.1)	27.8 (16 to 41.4)	-13.4 (-30 to 0.1)	-16.7 (-32.6 to -1.2)	-12 (-31.4 to 6.1)
	DALYs	Attributed all ages number	90,532 (54,975 to 130,403)	35,030 (19,041 to 52,945)	55,502 (34,749 to 78,859)	125,845 (66,689 to 191,108)	46,491 (21,577 to 75,556)	79,355 (44,094 to 118,196)	39 (8.2 to 69.2)	32.7 (-0.2 to 65.7)	43 (8.1 to 75.6)
		Attributed age-standardized rate (per 100,000)	599 (359.5 to 861.8)	462.8 (249.9 to 705.7)	740.1 (0 to 1042.6)	426.6 (230 to 636.6)	316.8 (154.7 to 504.3)	539.2 (304.1 to 789.9)	-28.8 (-43 to -16.5)	-31.6 (-45 to -18)	-27.1 (-43.5 to -13)
	YLLs	Attributed all ages number	69,663 (37,849 to 104,903)	25,925 (12,093 to 41,560)	43,738 (25,168 to 63,570)	105,027 (49,983 to 167,436)	37,742 (14,941 to 65,416)	67,285 (34,335 to 103,659)	50.8 (13 to 84.2)	45.6 (8.2 to 82)	53.8 (10.1 to 90.5)
		Attributed age-standardized rate (per 100,000)	511 (283.6 to 751.8)	386.6 (188.9 to 605.2)	641.2 (0 to 933.1)	365.6 (184.4 to 569.4)	265.5 (115.6 to 443.5)	468.6 (250.2 to 709)	-28.5 (-44.9 to -14.7)	-31.3 (-47 to -15.9)	-26.9 (-45.6 to -10.7)
	YLDs	Attributed all ages number	20,868 (10,757 to 34,838)	9,105 (4,471 to 15,407)	11,763 (6,035 to 19,554)	20,818 (11,034 to 34,100)	8,748 (4,455 to 14,459)	12,070 (6,412 to 19,639)	-0.2 (-11.5 to 16.9)	-3.9 (-17.6 to 14.1)	2.6 (-9.1 to 20.9)
		Attributed age-standardized rate (per 100,000)	88 (47.4 to 140.1)	76.3 (40.9 to 123.2)	98.9 (0 to 159.7)	60.9 (33.6 to 98.2)	51.3 (26.4 to 83.4)	70.6 (39.1 to 112.5)	-30.4 (-36.6 to -23.8)	-32.8 (-40.7 to -25.5)	-28.6 (-35 to -21.2)
Oman	Deaths	Attributed all ages number	154 (79 to 241)	58 (27 to 92)	96 (51 to 150)	164 (71 to 269)	63 (25 to 108)	101 (47 to 165)	6.9 (-21.9 to 40.9)	10.2 (-22.4 to 42.1)	4.9 (-26.4 to 42.5)
		Attributed age-standardized rate (per 100,000)	29 (15.5 to 45.3)	24.2 (11.5 to 38.4)	34.5 (0 to 52.4)	18.2 (9.1 to 28.3)	15.2 (6.8 to 24.7)	21.5 (11.4 to 33.4)	-37.8 (-51.2 to -22)	-37.3 (-53.1 to -20.5)	-37.6 (-51.6 to -19.6)

Country	Measure	Age, Metric	Year						% Change (1990 to 2019)		
			1990			2019					
			Both	Female	Male	Both	Female	Male	Both	Female	Male
	DALYs	Attributed all ages number	4,717 (2,576 to 7,157)	1,576 (789 to 2,459)	3,140 (1,746 to 4,809)	4,537 (2,191 to 7,491)	1,560 (664 to 2,602)	2,978 (1,455 to 4,853)	-3.8 (-28.9 to 24)	-1.1 (-27.5 to 25.4)	-5.2 (-32.8 to 27)
		Attributed age-standardized rate (per 100,000)	636 (344.4 to 972.1)	502.1 (242 to 792)	747.2 (0 to 1126.6)	305.9 (149.3 to 481.8)	254.3 (107.6 to 414.7)	354.2 (188.5 to 540.8)	-51.9 (-63 to -38.1)	-49.4 (-62.3 to -36.2)	-52.6 (-64 to -38.9)
	YLLs	Attributed all ages number	3,944 (1,921 to 6,347)	1,288 (555 to 2,099)	2,655 (1,346 to 4,261)	3,346 (1,226 to 5,911)	1,186 (393 to 2,148)	2,160 (842 to 3,801)	-15.1 (-45 to 17.2)	-7.9 (-40.5 to 22.3)	-18.7 (-49.4 to 15.8)
		Attributed age-standardized rate (per 100,000)	586 (300.8 to 916.3)	457.4 (206.4 to 738.6)	693.4 (0 to 1070.6)	271 (122.4 to 434)	222.8 (87.9 to 375.3)	316.7 (158.2 to 496.6)	-53.8 (-65.4 to -39.6)	-51.3 (-65 to -37.4)	-54.3 (-66.2 to -40)
	YLDs	Attributed all ages number	773 (393 to 1,323)	288 (142 to 497)	485 (245 to 830)	1,191 (565 to 2,047)	373 (181 to 642)	818 (381 to 1,408)	54 (32.3 to 76.2)	29.6 (7.5 to 55.2)	68.6 (42.6 to 93.2)
		Attributed age-standardized rate (per 100,000)	50 (28.3 to 79.4)	44.7 (24.4 to 71.3)	53.8 (0 to 84.4)	35 (18.3 to 55.5)	31.5 (16.2 to 50.3)	37.5 (19.8 to 59.7)	-29.9 (-38.7 to -21.3)	-29.5 (-40.3 to -19.5)	-30.2 (-40.7 to -21)
Palestine	Deaths	Attributed all ages number	226 (134 to 330)	89 (49 to 135)	137 (84 to 199)	319 (172 to 492)	134 (66 to 216)	185 (104 to 277)	41.2 (8.8 to 81)	51 (13.8 to 93.3)	34.9 (1.2 to 75.5)
		Attributed age-standardized rate (per 100,000)	30 (17.9 to 43.8)	21.8 (12.2 to 33.1)	40.6 (0 to 58.7)	19.5 (10.9 to 29.2)	15 (7.7 to 23.7)	26.5 (16.4 to 38)	-35.2 (-48.7 to -17.4)	-31.4 (-46.9 to -13.2)	-34.8 (-48.6 to -16.5)
	DALYs	Attributed all ages number	6,499 (4,124 to 9,164)	2,482 (1,498 to 3,603)	4,017 (2,587 to 5,592)	8,614 (5,037 to 12,805)	3,292 (1,804 to 4,996)	5,321 (3,155 to 7,785)	32.5 (5.1 to 61.9)	32.6 (5.4 to 62.4)	32.5 (2.7 to 64.9)
		Attributed age-standardized rate (per 100,000)	649 (400.4 to 925.5)	459.3 (266.8 to 689.5)	881.1 (0 to 1252.4)	364.5 (213.7 to 544.2)	270.4 (146.5 to 411.8)	478 (291.7 to 692.7)	-43.8 (-55.2 to -30.2)	-41.1 (-53.7 to -27.5)	-45.8 (-57.4 to -31.4)
	YLLs	Attributed all ages number	4,542 (2,585 to 6,829)	1,647 (854 to 2,605)	2,894 (1,698 to 4,270)	5,863 (2,847 to 9,454)	2,128 (935 to 3,611)	3,735 (1,864 to 5,882)	29.1 (-9.2 to 70)	29.2 (-9.4 to 69.9)	29.1 (-10.7 to 73.7)
		Attributed age-standardized rate (per 100,000)	549 (319 to 809.8)	372.8 (199.1 to 577.9)	766.9 (0 to 1117.8)	298.2 (157.8 to 463.1)	213.4 (101.2 to 349.6)	402.2 (231.3 to 602.9)	-45.7 (-58.3 to -29.5)	-42.8 (-57.9 to -25.8)	-47.6 (-60.3 to -30.7)

Country	Measure	Age, Metric	Year						% Change (1990 to 2019)		
			1990			2019					
			Both	Female	Male	Both	Female	Male	Both	Female	Male
	YLDs	Attributed all ages number	1,958 (956 to 3,309)	835 (402 to 1,423)	1,123 (557 to 1,898)	2,751 (1,336 to 4,612)	1,164 (542 to 2,009)	1,586 (764 to 2,729)	40.5 (25.4 to 56.4)	39.5 (17.5 to 62.2)	41.3 (23 to 61.3)
		Attributed age-standardized rate (per 100,000)	100 (53.8 to 162.7)	86.4 (45.1 to 141.4)	114.2 (0 to 182.1)	66.3 (35.3 to 108.1)	57 (30.1 to 93.6)	75.8 (40.9 to 121.3)	-33.7 (-39.6 to -27.3)	-34.1 (-42.5 to -25.9)	-33.6 (-40.6 to -25.5)
Qatar	Deaths	Attributed all ages number	8 (1 to 16)	2 (to 5)	6 (1 to 11)	21 (3 to 47)	5 (to 12)	16 (2 to 36)	174.6 (90.4 to 287)	125.5 (25.6 to 238.1)	194.1 (96.8 to 342.6)
		Attributed age-standardized rate (per 100,000)	11 (1.8 to 21.6)	9.1 (0.7 to 18.3)	12.8 (0 to 24)	8.1 (2.3 to 15.2)	9.4 (1.7 to 18.7)	7.7 (2.4 to 14.3)	-27.2 (-43.6 to 34.6)	3.7 (-18 to 126.4)	-39.7 (-54.4 to 1.6)
	DALYs	Attributed all ages number	308 (81 to 588)	78 (18 to 153)	230 (62 to 439)	976 (290 to 1,882)	198 (52 to 406)	778 (237 to 1,485)	217.1 (140.7 to 351.2)	155 (100.9 to 237.9)	238.1 (146.5 to 399)
		Attributed age-standardized rate (per 100,000)	223 (48 to 419)	177.2 (24.3 to 356.1)	252.5 (0 to 463.4)	123.8 (38.5 to 232.8)	133.6 (27.2 to 266.5)	121.1 (41.3 to 224)	-44.5 (-56.3 to -14.3)	-24.6 (-39.9 to 24.5)	-52 (-63.6 to -26.4)
	YLLs	Attributed all ages number	217 (18 to 462)	50 (2 to 119)	166 (16 to 349)	535 (45 to 1,284)	108 (3 to 269)	427 (40 to 1,026)	146.8 (52.6 to 244)	113.1 (18 to 258)	157.1 (47.5 to 278.1)
		Attributed age-standardized rate (per 100,000)	198 (28.9 to 387.7)	153.9 (8.4 to 322.2)	226.3 (0 to 429.2)	104.9 (24.4 to 205.7)	117.3 (16.5 to 243.9)	101.2 (26.7 to 194.6)	-47 (-59.6 to -8.3)	-23.8 (-40.7 to 92.5)	-55.3 (-66.7 to -26.2)
	YLDs	Attributed all ages number	91 (37 to 160)	27 (11 to 48)	64 (26 to 113)	441 (161 to 815)	90 (36 to 168)	350 (127 to 644)	384.7 (297.3 to 459.4)	233.2 (168.4 to 300.1)	449 (331.8 to 539.2)
		Attributed age-standardized rate (per 100,000)	25 (10.3 to 42.4)	23.3 (9.1 to 39.9)	26.2 (0 to 44.1)	18.9 (8.2 to 32.7)	16.3 (6.5 to 29.2)	19.9 (8.6 to 34.7)	-24.8 (-34.3 to -15.4)	-29.9 (-40.3 to -20.7)	-23.9 (-35.7 to -11.8)
Saudi Arabia	Deaths	Attributed all ages number	1,161 (676 to 1,716)	331 (147 to 532)	829 (510 to 1,200)	1,960 (942 to 3,146)	465 (143 to 869)	1,495 (775 to 2,301)	68.8 (14.6 to 126.2)	40.4 (-15.1 to 94.7)	80.2 (21.4 to 143)
		Attributed age-standardized rate (per 100,000)	23 (13.5 to 33.3)	15.6 (7.4 to 24.9)	28.7 (0 to 40.6)	16.2 (9.1 to 24.1)	10.2 (4.1 to 17.7)	20.5 (12.5 to 29.1)	-29.2 (-45.3 to -10.6)	-34.5 (-53.2 to -13.9)	-28.8 (-44.8 to -9.4)

Country	Measure	Age, Metric	Year						% Change (1990 to 2019)		
			1990			2019					
			Both	Female	Male	Both	Female	Male	Both	Female	Male
	DALYs	Attributed all ages number	35,138 (20,971 to 51,220)	9,906 (4,631 to 15,676)	25,232 (16,043 to 36,364)	58,724 (26,928 to 97,434)	13,999 (4,269 to 26,497)	44,724 (22,338 to 71,147)	67.1 (9.1 to 128.7)	41.3 (-15.7 to 98.5)	77.3 (15.5 to 145.2)
		Attributed age-standardized rate (per 100,000)	522 (311.3 to 756.8)	352.6 (162.9 to 560.3)	643.3 (0 to 916.3)	326.7 (172.9 to 497.9)	200.5 (74.2 to 348.8)	411.6 (237.6 to 602.7)	-37.4 (-53.8 to -19.2)	-43.1 (-60.4 to -24.9)	-36 (-52.8 to -17)
	YLLs	Attributed all ages number	29,710 (16,583 to 45,270)	7,810 (2,990 to 13,085)	21,900 (13,118 to 32,477)	49,531 (20,230 to 84,777)	10,864 (2,323 to 22,146)	38,667 (17,550 to 63,300)	66.7 (-0.2 to 138)	39.1 (-34.8 to 110.3)	76.6 (6.3 to 153)
		Attributed age-standardized rate (per 100,000)	475 (276.8 to 707.2)	311.8 (133 to 505.8)	591.6 (0 to 860.1)	291.1 (150.1 to 449.7)	171.7 (57.8 to 310.2)	371.3 (206.4 to 548.6)	-38.7 (-56.4 to -18.9)	-44.9 (-64.1 to -25.1)	-37.2 (-55.4 to -17)
	YLDs	Attributed all ages number	5,428 (2,923 to 8,873)	2,096 (1,042 to 3,493)	3,332 (1,829 to 5,344)	9,192 (4,541 to 14,774)	3,135 (1,383 to 5,236)	6,057 (3,109 to 9,662)	69.4 (41.7 to 103.8)	49.6 (17.6 to 89.9)	81.8 (50.1 to 118.3)
		Attributed age-standardized rate (per 100,000)	47 (27.5 to 70.2)	40.8 (21.8 to 63.9)	51.7 (0 to 76.2)	35.7 (19.4 to 54.2)	28.8 (13.8 to 46.7)	40.4 (23.1 to 60.3)	-24.4 (-34.7 to -14.4)	-29.5 (-42 to -17.8)	-21.9 (-33.3 to -11.4)
Sudan	Deaths	Attributed all ages number	5,296 (3,771 to 7,294)	1,913 (1,296 to 2,669)	3,383 (2,417 to 4,682)	7,701 (5,353 to 10,883)	2,666 (1,787 to 3,755)	5,035 (3,416 to 7,186)	45.4 (17.6 to 81.8)	39.4 (16.2 to 71.9)	48.8 (17.7 to 90.2)
		Attributed age-standardized rate (per 100,000)	63 (45 to 87)	48.7 (33.1 to 68.7)	77.2 (0 to 106.7)	50.3 (35.2 to 70.3)	38.8 (26.5 to 54.6)	60.1 (41.8 to 85.8)	-20.6 (-34.2 to -2.7)	-20.3 (-32.1 to -4.6)	-22.1 (-37.4 to -2.2)
	DALYs	Attributed all ages number	164,967 (122,211 to 219,066)	59,842 (41,931 to 80,578)	105,125 (76,675 to 139,744)	207,362 (144,189 to 286,542)	72,643 (49,291 to 100,934)	134,720 (93,450 to 185,045)	25.7 (2.8 to 55.6)	21.4 (1.3 to 47.2)	28.2 (2.5 to 60.7)
		Attributed age-standardized rate (per 100,000)	1,546 (1136.5 to 2077.3)	1162.7 (806.5 to 1585.5)	1902.6 (0 to 2562.7)	1041.6 (736.8 to 1419.6)	785.5 (543.1 to 1069.2)	1262.1 (886 to 1741.9)	-32.6 (-44.8 to -16.6)	-32.4 (-43 to -18)	-33.7 (-47 to -16.3)
	YLLs	Attributed all ages number	133,822 (94,146 to 185,851)	46,844 (30,745 to 66,226)	86,978 (61,106 to 120,692)	163,937 (108,842 to 237,107)	54,000 (33,586 to 78,480)	109,937 (73,016 to 158,502)	22.5 (-5.5 to 59)	15.3 (-8.4 to 46.7)	26.4 (-4.5 to 67.1)

Country	Measure	Age, Metric	Year						% Change (1990 to 2019)		
			1990			2019					
			Both	Female	Male	Both	Female	Male	Both	Female	Male
		Attributed age-standardized rate (per 100,000)	1,383 (977.1 to 1907.6)	1023.3 (685.9 to 1433.2)	1717 (0 to 2381.1)	912.7 (624.9 to 1280.3)	672.5 (450.3 to 948.7)	1118.2 (763.1 to 1591.5)	-34 (-47.5 to -16.3)	-34.3 (-46.2 to -17.6)	-34.9 (-49.7 to -15.7)
	YLDs	Attributed all ages number	31,145 (16,663 to 50,615)	12,998 (6,771 to 21,164)	18,147 (9,743 to 29,245)	43,426 (23,185 to 69,760)	18,643 (9,745 to 30,679)	24,783 (13,576 to 39,657)	39.4 (30.5 to 50.3)	43.4 (32.9 to 55.7)	36.6 (24.9 to 51.4)
		Attributed age-standardized rate (per 100,000)	163 (95 to 249.5)	139.4 (79 to 213.8)	185.6 (0 to 285.1)	129 (78.3 to 193.7)	113 (67.3 to 171.6)	143.8 (86.2 to 217.1)	-20.8 (-25.6 to -14.2)	-18.9 (-24.8 to -11.1)	-22.5 (-28.3 to -14.1)
Syrian Arab Republic	Deaths	Attributed all ages number	1,710 (1,048 to 2,435)	587 (319 to 891)	1,123 (711 to 1,575)	2,687 (1,561 to 4,063)	908 (479 to 1,421)	1,779 (1,052 to 2,654)	57.2 (15 to 113.9)	54.7 (16.2 to 108.4)	58.5 (12.5 to 119.7)
		Attributed age-standardized rate (per 100,000)	36 (22.5 to 51.8)	27.8 (15.4 to 42.1)	43.6 (0 to 61.1)	29.8 (17.9 to 44.2)	25.5 (14.2 to 39)	34.6 (21.3 to 51.2)	-17.4 (-37.4 to 9)	-8 (-28.4 to 19.6)	-20.5 (-41.5 to 7.2)
	DALYs	Attributed all ages number	54,626 (35,690 to 75,346)	18,413 (10,974 to 26,521)	36,213 (24,551 to 49,279)	67,971 (40,492 to 99,934)	22,631 (12,341 to 34,014)	45,340 (27,351 to 66,821)	24.4 (-6.4 to 68.9)	22.9 (-3.3 to 63)	25.2 (-8.5 to 71.4)
		Attributed age-standardized rate (per 100,000)	866 (558.9 to 1206.6)	614.8 (358.2 to 901.9)	1098.7 (0 to 1518.4)	577.8 (352.4 to 834.8)	435 (246.7 to 641.5)	724.1 (437.6 to 1047.9)	-33.3 (-49.5 to -11.7)	-29.2 (-44.5 to -9.1)	-34.1 (-51.4 to -10.3)
	YLLs	Attributed all ages number	42,544 (25,164 to 62,041)	13,307 (6,763 to 20,724)	29,236 (18,221 to 42,200)	55,976 (30,367 to 87,059)	17,362 (8,403 to 28,126)	38,614 (21,409 to 59,402)	31.6 (-7.1 to 87.2)	30.5 (-3.9 to 83)	32.1 (-9.5 to 89.4)
		Attributed age-standardized rate (per 100,000)	764 (462.3 to 1092.6)	525.7 (281.3 to 797.9)	983.1 (0 to 1398.3)	491.5 (281.4 to 746.8)	360.4 (188.1 to 565.5)	625.7 (355.1 to 940.3)	-35.6 (-53.7 to -11.3)	-31.4 (-48.2 to -8.1)	-36.4 (-55.6 to -10.1)
	YLDs	Attributed all ages number	12,082 (6,303 to 20,191)	5,106 (2,526 to 8,595)	6,976 (3,663 to 11,622)	11,995 (6,599 to 19,264)	5,269 (2,793 to 8,754)	6,726 (3,679 to 10,491)	-0.7 (-8.9 to 13.5)	3.2 (-6.3 to 17.7)	-3.6 (-13.2 to 11.6)
		Attributed age-standardized rate (per 100,000)	103 (58 to 161)	89.1 (50 to 139.5)	115.6 (0 to 180)	86.3 (48.2 to 135.8)	74.6 (40.7 to 121.2)	98.4 (55.1 to 153)	-16 (-21.1 to -11.1)	-16.3 (-22.9 to -10.6)	-14.9 (-21.3 to -8.8)

Country	Measure	Age, Metric	Year						% Change (1990 to 2019)		
			1990			2019					
			Both	Female	Male	Both	Female	Male	Both	Female	Male
Tunisia	Deaths	Attributed all ages number	915 (567 to 1,296)	318 (169 to 483)	597 (384 to 835)	1,802 (1,004 to 2,791)	685 (341 to 1,105)	1,117 (648 to 1,720)	96.9 (46 to 158.8)	115.1 (58.4 to 181.5)	87.1 (35.9 to 151.6)
		Attributed age-standardized rate (per 100,000)	22 (13.8 to 32.4)	16.7 (8.9 to 25.6)	28 (0 to 40.2)	16.5 (9.4 to 25.5)	11.9 (6 to 19.2)	21.9 (12.9 to 32.9)	-25.8 (-44.1 to -4)	-28.6 (-46.4 to -9.1)	-21.9 (-41.7 to 3.2)
	DALYs	Attributed all ages number	26,998 (17,391 to 36,941)	9,631 (5,572 to 13,962)	17,368 (11,603 to 23,437)	37,357 (21,342 to 57,240)	13,469 (7,010 to 21,301)	23,887 (13,869 to 36,574)	38.4 (4.3 to 81.6)	39.9 (5.1 to 80)	37.5 (2.6 to 83.5)
		Attributed age-standardized rate (per 100,000)	497 (319.8 to 692)	365.1 (211.3 to 532.6)	625.7 (0 to 857.9)	313.2 (180.6 to 477.4)	221.1 (117 to 345.8)	412.5 (248.7 to 625.8)	-37 (-51.4 to -19.3)	-39.4 (-52.8 to -23.8)	-34.1 (-49.8 to -13.5)
	YLLs	Attributed all ages number	20,173 (12,314 to 28,776)	6,666 (3,376 to 10,195)	13,507 (8,535 to 18,886)	30,622 (15,688 to 49,104)	10,509 (4,697 to 17,361)	20,113 (10,734 to 32,495)	51.8 (8.6 to 104.8)	57.6 (13 to 109.8)	48.9 (4.5 to 106.2)
		Attributed age-standardized rate (per 100,000)	412 (250.9 to 589.1)	290.5 (150.8 to 438.3)	531 (0 to 742.2)	256.5 (135.5 to 408.9)	172 (78.8 to 282.4)	348.1 (192.7 to 545.4)	-37.8 (-54.7 to -17.3)	-40.8 (-57.1 to -22.2)	-34.4 (-53.2 to -10.6)
	YLDs	Attributed all ages number	6,825 (3,491 to 11,158)	2,965 (1,506 to 4,902)	3,861 (1,978 to 6,295)	6,735 (3,678 to 10,622)	2,961 (1,555 to 4,861)	3,775 (2,164 to 5,870)	-1.3 (-14.2 to 22.1)	-0.1 (-15.6 to 23.7)	-2.2 (-15.4 to 21.3)
		Attributed age-standardized rate (per 100,000)	85 (46.5 to 135)	74.7 (40.6 to 120)	94.7 (0 to 150)	56.7 (31.1 to 90.1)	49.2 (26 to 80.8)	64.4 (36.7 to 100.9)	-33.2 (-39.6 to -25.9)	-34.2 (-42 to -25.6)	-32 (-39 to -23.7)
Turkey	Deaths	Attributed all ages number	3,647 (1,268 to 6,179)	1,414 (411 to 2,585)	2,233 (852 to 3,753)	4,678 (1,470 to 8,568)	2,138 (558 to 4,094)	2,540 (889 to 4,574)	28.3 (-2.8 to 60.8)	51.2 (11.2 to 88.7)	13.8 (-15.5 to 48.2)
		Attributed age-standardized rate (per 100,000)	12 (4.2 to 19.9)	8.7 (2.6 to 15.9)	14.8 (0 to 24.5)	5.9 (1.9 to 10.7)	4.8 (1.3 to 9.2)	7.1 (2.6 to 12.5)	-49.4 (-61.3 to -37.4)	-44.8 (-60.3 to -31.3)	-51.9 (-63.6 to -38.5)
	DALYs	Attributed all ages number	109,173 (49,043 to 173,666)	38,943 (16,451 to 65,031)	70,230 (32,538 to 112,939)	94,642 (34,266 to 171,179)	38,241 (12,928 to 70,370)	56,402 (21,025 to 102,021)	-13.3 (-36.3 to 8.9)	-1.8 (-31.9 to 23.4)	-19.7 (-41.8 to 3)

Country	Measure	Age, Metric	Year						% Change (1990 to 2019)		
			1990			2019					
			Both	Female	Male	Both	Female	Male	Both	Female	Male
		Attributed age-standardized rate (per 100,000)	275 (117.7 to 444.5)	193.2 (76.1 to 327.4)	361.6 (0 to 587.4)	112.5 (42.2 to 201.2)	85.8 (29.9 to 155.6)	142 (55.4 to 251.2)	-59.1 (-68.9 to -50.2)	-55.6 (-66.2 to -46.1)	-60.7 (-70.3 to -50.5)
	YLLs	Attributed all ages number	79,319 (25,148 to 138,851)	25,856 (6,614 to 47,781)	53,463 (18,122 to 93,449)	71,076 (16,890 to 141,441)	27,901 (5,739 to 56,005)	43,174 (11,282 to 84,019)	-10.4 (-38.8 to 16.2)	7.9 (-27.1 to 38)	-19.2 (-46.7 to 8.5)
		Attributed age-standardized rate (per 100,000)	223 (75.1 to 382.7)	146.6 (39.6 to 267.4)	304.3 (0 to 521.9)	84.7 (21.5 to 165.9)	61.8 (13 to 123.8)	110.4 (31.7 to 207.4)	-62.1 (-74 to -51.3)	-57.9 (-72.3 to -46)	-63.7 (-75.3 to -51.8)
	YLDs	Attributed all ages number	29,854 (14,031 to 51,234)	13,087 (6,142 to 22,665)	16,766 (7,812 to 28,435)	23,567 (10,432 to 39,383)	10,339 (4,466 to 17,462)	13,227 (6,057 to 22,572)	-21.1 (-37.2 to -1.1)	-21 (-39.6 to 2.5)	-21.1 (-36.5 to -0.5)
		Attributed age-standardized rate (per 100,000)	52 (26.1 to 86.4)	46.6 (23.1 to 78.4)	57.3 (0 to 93.7)	27.8 (12.6 to 46.5)	24 (10.4 to 40.4)	31.7 (14.8 to 53.8)	-46.5 (-55.9 to -39)	-48.5 (-58.3 to -39.8)	-44.7 (-54.6 to -36.2)
United Arab Emirates	Deaths	Attributed all ages number	50 (14 to 92)	13 (3 to 24)	37 (11 to 69)	125 (10 to 326)	20 (1 to 50)	105 (8 to 275)	149.4 (-39.3 to 307.5)	55.8 (-62.8 to 146.3)	181.8 (-31.7 to 377.5)
		Attributed age-standardized rate (per 100,000)	18 (6.7 to 30.8)	15.7 (4.9 to 27.8)	19 (0 to 32.8)	8.4 (2.2 to 16.9)	6.5 (1.4 to 12.8)	9.2 (2.4 to 18.5)	-52.8 (-69.7 to -39)	-58.6 (-74.6 to -45.5)	-51.7 (-70.4 to -35.3)
	DALYs	Attributed all ages number	1,892 (626 to 3,467)	439 (141 to 804)	1,453 (461 to 2,698)	5,247 (1,113 to 12,668)	878 (220 to 1,997)	4,369 (873 to 10,800)	177.4 (36.8 to 323.3)	100.1 (14.6 to 180.8)	200.8 (37.8 to 380.5)
		Attributed age-standardized rate (per 100,000)	363 (137.2 to 616.1)	296.5 (90 to 518.5)	398.2 (0 to 684.7)	150.8 (37.8 to 303.9)	112 (25.3 to 226.1)	166.4 (41.9 to 336.4)	-58.4 (-74.8 to -45)	-62.2 (-77.3 to -50.2)	-58.2 (-76.2 to -42.5)
	YLLs	Attributed all ages number	1,528 (347 to 2,953)	327 (63 to 659)	1,201 (274 to 2,345)	3,592 (150 to 10,529)	505 (17 to 1,457)	3,087 (124 to 9,096)	135.1 (-62.3 to 309.9)	54.6 (-77.2 to 159.6)	157 (-59.1 to 369.7)
		Attributed age-standardized rate (per 100,000)	332 (117.1 to 574)	267 (72.6 to 480.5)	366.3 (0 to 642.6)	128.9 (23.3 to 271.9)	91.8 (14.3 to 196.2)	143.8 (29.1 to 303)	-61.2 (-80.7 to -46.6)	-65.6 (-83.7 to -52.5)	-60.7 (-81.1 to -43.6)
	YLDs	Attributed all ages number	364 (151 to 632)	113 (44 to 195)	251 (107 to 445)	1,655 (680 to 2,946)	374 (143 to 686)	1,282 (520 to 2,272)	355 (279.7 to 443.1)	232 (167.2 to 323)	410 (315.3 to 528.3)

Country	Measure	Age, Metric	Year						% Change (1990 to 2019)		
			1990			2019					
			Both	Female	Male	Both	Female	Male	Both	Female	Male
		Attributed age-standardized rate (per 100,000)	31 (14.8 to 50.7)	29.5 (13 to 49.7)	31.9 (0 to 52.1)	21.9 (9.4 to 38.6)	20.2 (8.1 to 36.2)	22.5 (9.4 to 39.2)	-29.5 (-42 to -21.3)	-31.5 (-45.2 to -20.5)	-29.4 (-43.1 to -18.8)
Yemen	Deaths	Attributed all ages number	3,032 (2,126 to 4,141)	1,155 (786 to 1,624)	1,877 (1,319 to 2,593)	6,812 (4,785 to 9,331)	2,512 (1,670 to 3,523)	4,300 (3,016 to 5,923)	124.7 (72.3 to 200.1)	117.6 (69.9 to 188.6)	129.1 (69.9 to 211)
		Attributed age-standardized rate (per 100,000)	70 (49.7 to 96.2)	52.4 (36.1 to 73.7)	92.3 (0 to 124.7)	64 (44.3 to 85.9)	48 (31.8 to 67)	81.1 (58 to 109.8)	-8.8 (-28.1 to 18.8)	-8.5 (-27.2 to 18.3)	-12.1 (-31.7 to 16)
	DALYs	Attributed all ages number	100,753 (72,596 to 134,425)	36,011 (24,984 to 48,786)	64,742 (46,878 to 86,615)	198,144 (139,198 to 263,938)	71,659 (48,354 to 97,381)	126,485 (89,402 to 168,055)	96.7 (54.6 to 154.5)	99 (58.2 to 155.6)	95.4 (51.6 to 158)
		Attributed age-standardized rate (per 100,000)	1,714 (1225.9 to 2321.9)	1223.1 (855.4 to 1697.9)	2237.6 (0 to 3009.5)	1362.8 (971.8 to 1816.5)	987.6 (681.3 to 1339.4)	1750.8 (1263.4 to 2347.9)	-20.5 (-37.5 to 3.7)	-19.3 (-36.1 to 5)	-21.8 (-39.6 to 3.4)
	YLLs	Attributed all ages number	81,983 (56,959 to 113,322)	28,358 (18,837 to 40,785)	53,626 (36,529 to 74,883)	158,300 (108,684 to 221,803)	54,697 (35,068 to 79,192)	103,604 (71,213 to 145,341)	93.1 (42.5 to 166.6)	92.9 (44.2 to 163.5)	93.2 (40.9 to 174)
		Attributed age-standardized rate (per 100,000)	1,562 (1091.2 to 2137.5)	1095.8 (742.2 to 1556.1)	2061.3 (0 to 2823.7)	1211.3 (843.2 to 1671.1)	858.7 (568.6 to 1218.1)	1576.6 (1121.4 to 2181.4)	-22.5 (-41.3 to 4)	-21.6 (-40 to 5.4)	-23.5 (-42.8 to 3.6)
	YLDs	Attributed all ages number	18,770 (10,131 to 30,261)	7,653 (4,031 to 12,669)	11,116 (6,024 to 17,908)	39,844 (21,558 to 64,684)	16,962 (8,969 to 28,194)	22,881 (12,318 to 36,576)	112.3 (98.1 to 126.1)	121.6 (102.5 to 140)	105.8 (88 to 125.5)
		Attributed age-standardized rate (per 100,000)	152 (91.6 to 228.3)	127.3 (75.4 to 190.3)	176.3 (0 to 267.2)	151.5 (91.5 to 227.9)	128.9 (75.8 to 195.8)	174.2 (105.5 to 260.7)	0 (-4.9 to 5)	1.3 (-4.9 to 7.7)	-1.2 (-7.3 to 5.7)

Data in parentheses are 95% Uncertainty Intervals (95% UIs)

Abbreviations: DALYs: disability-adjusted life years; YLLs: years of life lost; YLDs: years lived with disability
