# Supplementary figures and images for: Estimating the burden of diseases attributable to lead exposure in the North Africa and Middle East region, 1990–2019: a systematic analysis for the Global Burden of Disease study 2019
Source: Environ Health. 2022 Oct 29;21:105. doi: 10.1186/s12940-022-00914-3 (PMC9617306; doi:10.1186/s12940-022-00914-3)

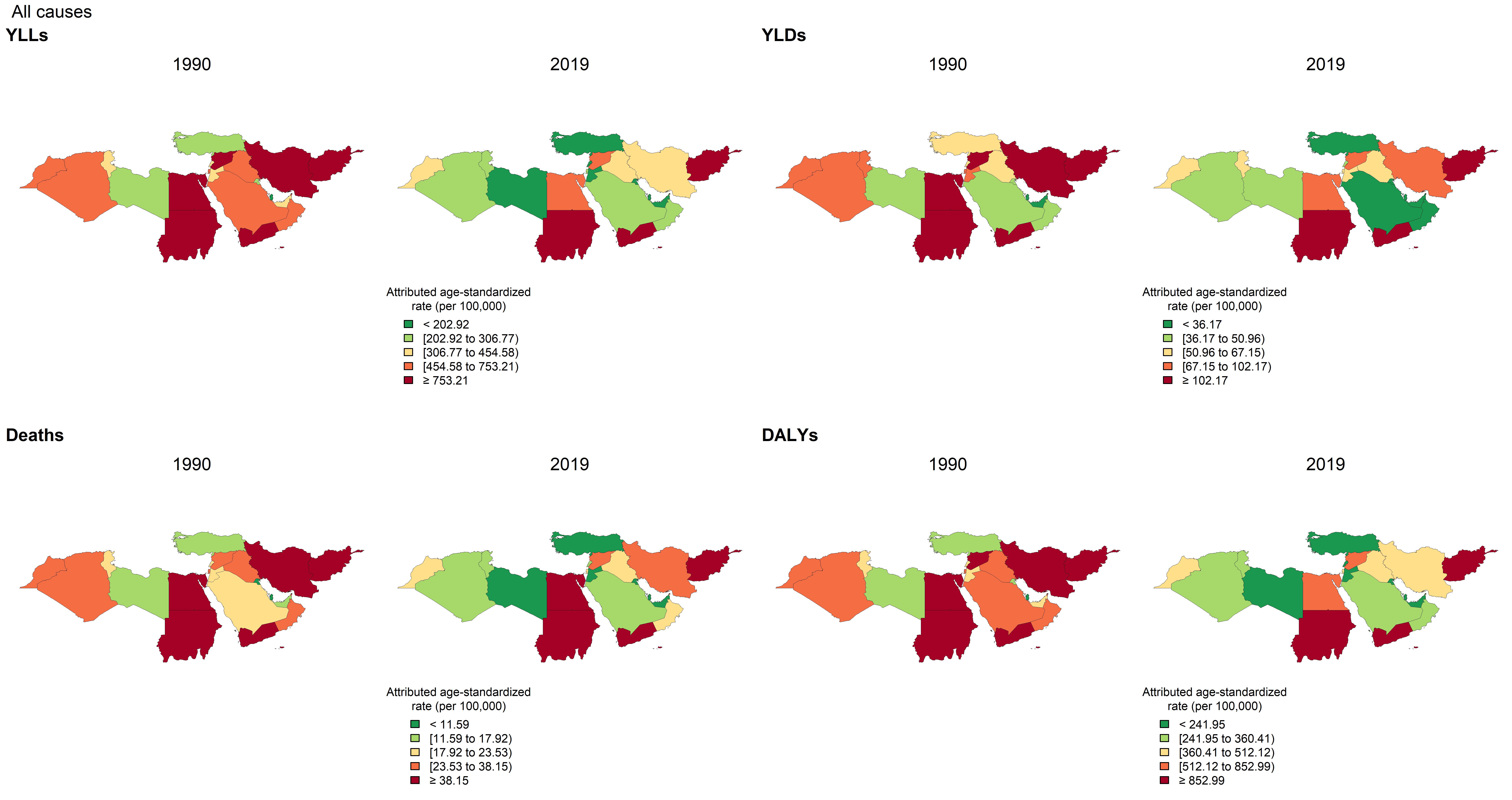

Supplement: Supplementary file 6 — Additional file 6: Figure S3. Geographical distribution of age-standardized rate of deaths, disability-adjusted life years (DALYs), years of life lost (YLLs), and years lived with disability (YLDs) attributable to lead exposure among both sexes in North Africa and Middle East countries in 1990 and 2019. [file 12940_2022_914_MOESM6_ESM.jpeg]

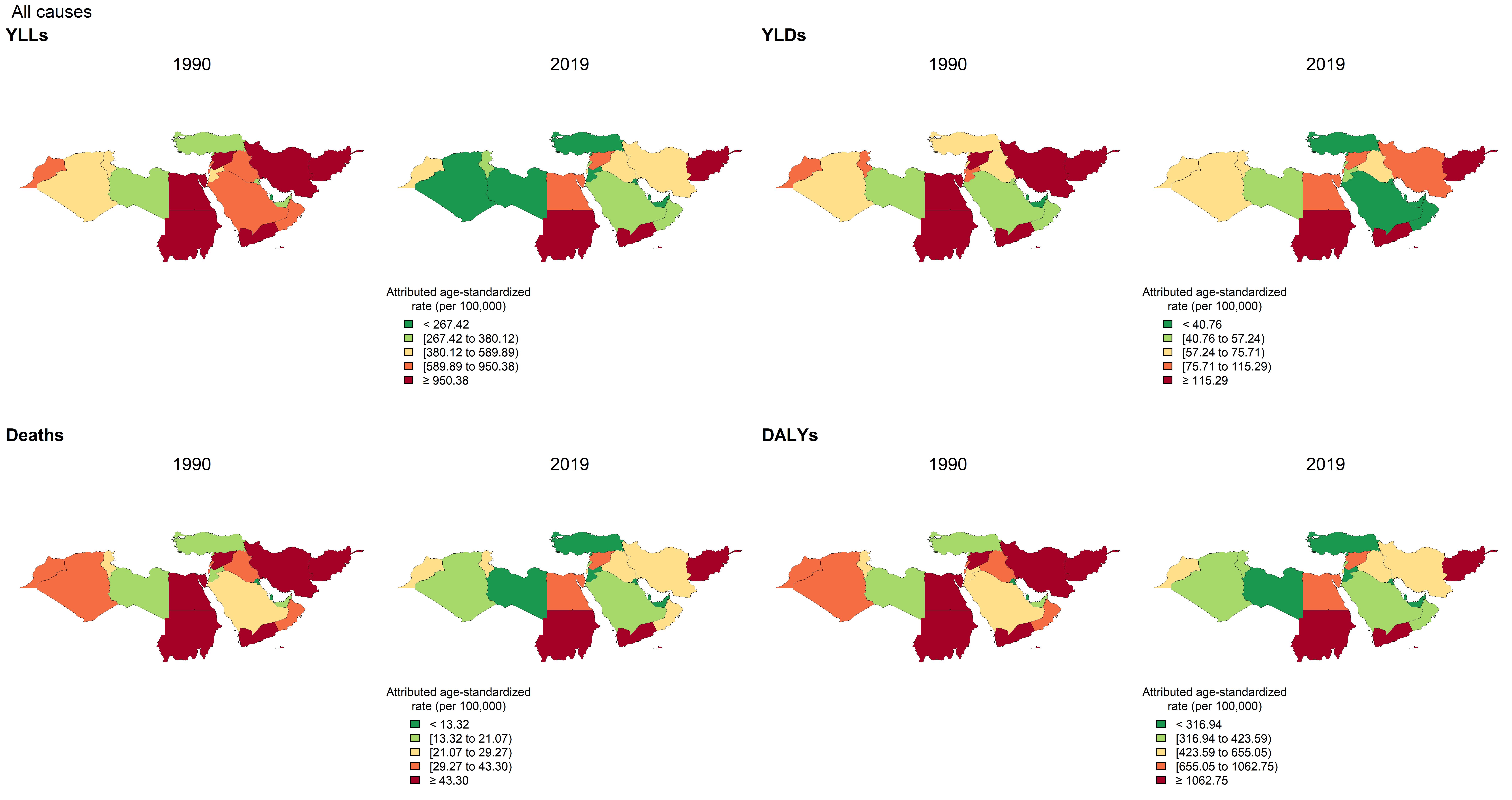

Supplement: Supplementary file 7 — Additional file 7: Figure S4. Geographical distribution of age-standardized rate of deaths, disability-adjusted life years (DALYs), years of life lost (YLLs), and years lived with disability (YLDs) attributable to lead exposure among men in North Africa and Middle East countries in 1990 and 2019. [file 12940_2022_914_MOESM7_ESM.jpeg]

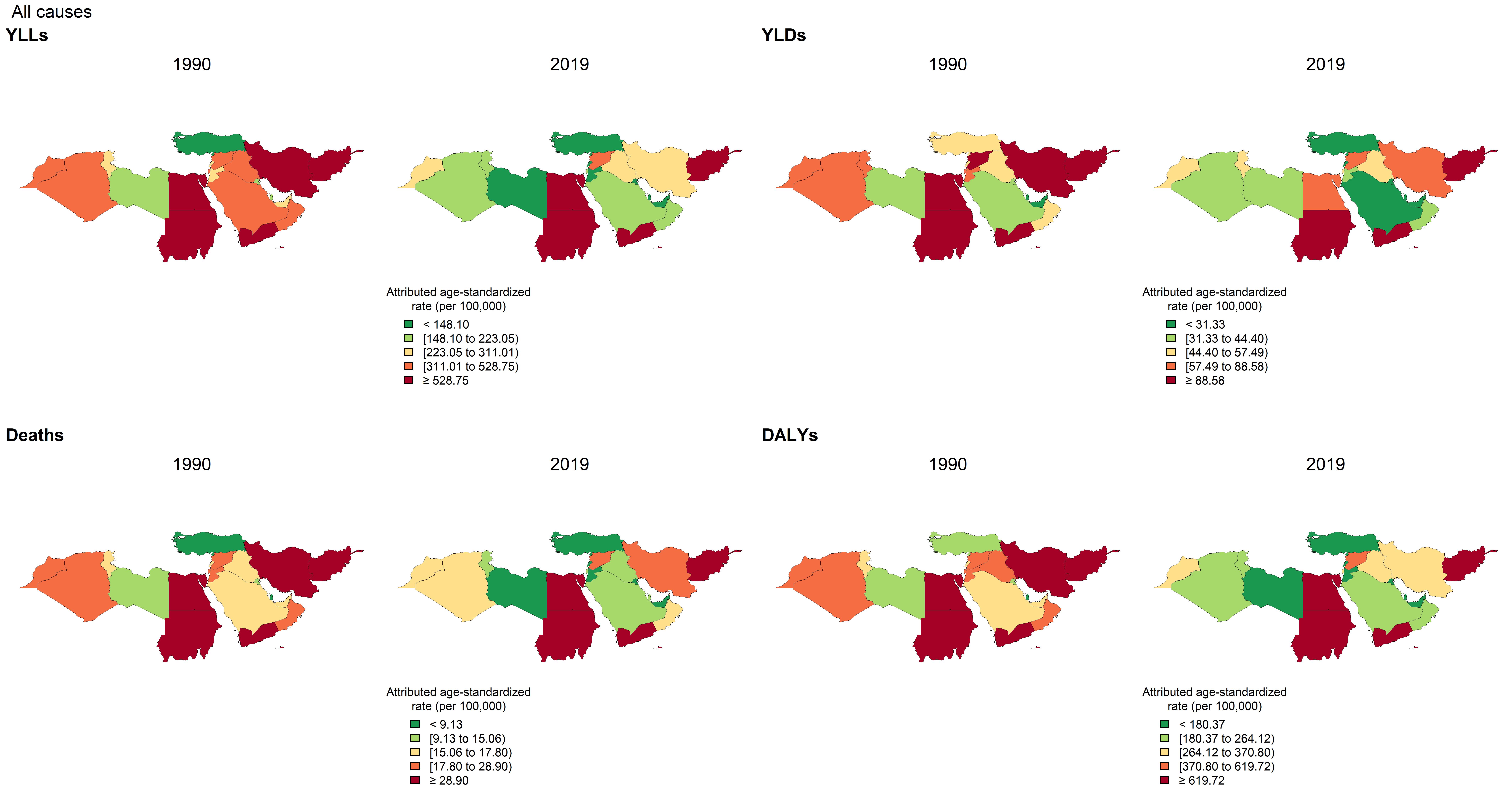

Supplement: Supplementary file 8 — Additional file 8: Figure S5. Geographical distribution of age-standardized rate of deaths, disability-adjusted life years (DALYs), years of life lost (YLLs), and years lived with disability (YLDs) attributable to lead exposure among women in North Africa and Middle East countries in 1990 and 2019. [file 12940_2022_914_MOESM8_ESM.jpeg]

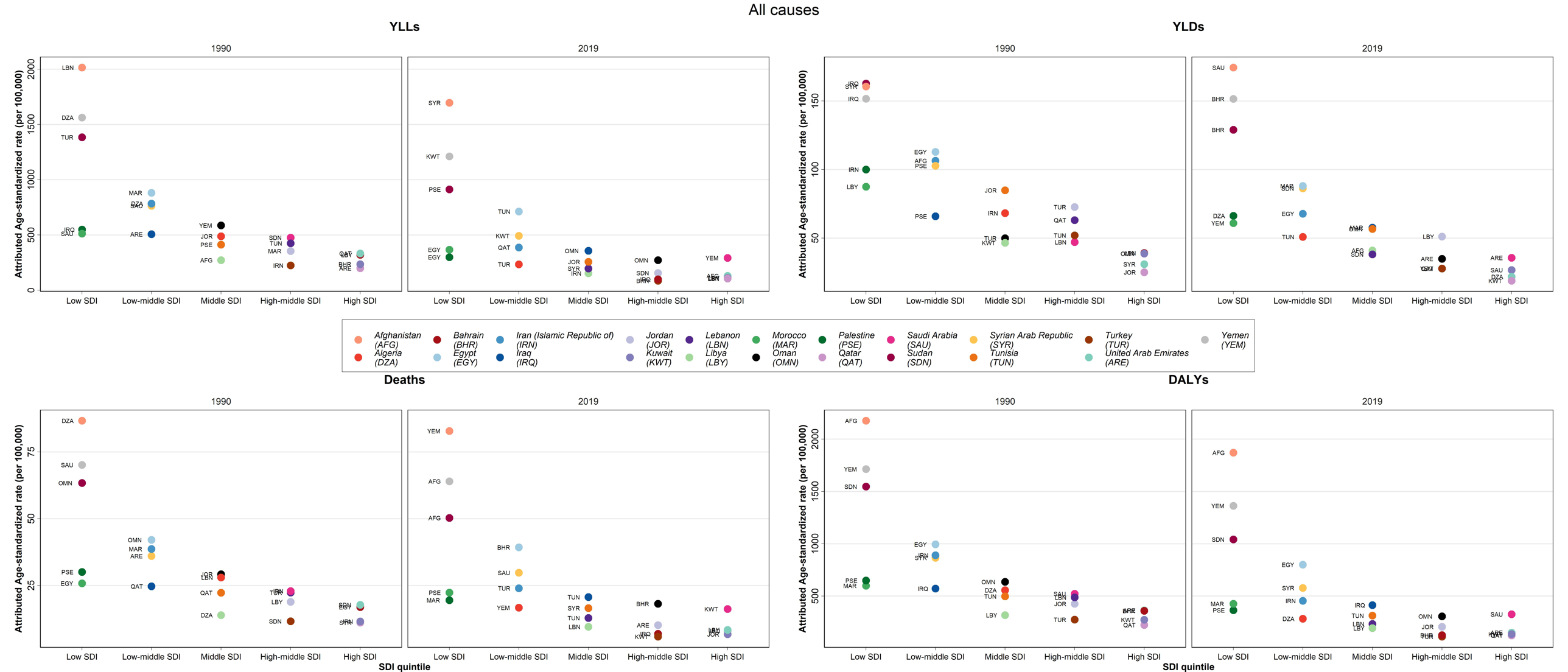

Supplement: Supplementary file 10 — Additional file 10: Figure S7. Age-standardized rate of deaths, disability-adjusted life years (DALYs), years of life lost (YLLs), and years lived with disability (YLDs) attributable to lead exposure in 21 countries of North Africa and Middle East region in 1990 and 2019, by sociodemographic index (SDI) quintiles. [file 12940_2022_914_MOESM10_ESM.jpeg]
